# Rational Design of Natural Xanthones Against Gram‐negative Bacteria

**DOI:** 10.1002/advs.202411923

**Published:** 2025-01-30

**Authors:** Xiaojia Liu, Meirong Song, Ying Liu, Shuyu Yang, Shang Chen, Jijun Kang, Jianzhong Shen, Kui Zhu

**Affiliations:** ^1^ National Key Laboratory of Veterinary Public Health and Safety College of Veterinary Medicine China Agricultural University No.2 Yuanmingyuan West Road Beijing 100193 China

**Keywords:** bacterial membrane, Gram‐negative bacterium, molecular design, natural product

## Abstract

Most antibiotics are ineffective against Gram‐negative bacteria owing to the existence of the outer membrane (OM) barrier. The rational design of compounds to expand their antibacterial spectra of antibiotics solely targeting Gram‐positive pathogens remains challenging. Here, the design of skeletons from natural products to penetrate the OM are deciphered. Structure‐activity relationship analysis shows the optimization of the model of natural xanthones α‐mangostin endows the broad‐spectrum antibacterial activity. Mechanistic studies demonstrate the lead compound A20 penetrates the OM in a self‐promoted pathway through electronic and hydrophobic interactions with lipopolysaccharides and phospholipids in OM. A20 displays rapid bactericidal activity by targeting the cofactor heme in the respiratory complex. The therapeutic efficacy of A20 is demonstrated in two animal models infected with multidrug‐resistant Gram‐negative bacterial pathogens. The findings elucidate the structural property and self‐promoted transportation of a class of antibacterial compounds, to facilitate the design and discovery of antibacterial agents against increasingly prevalent Gram‐negative pathogens associated with infections.

## Graphical abstract

The skeletons are screened and obtained from natural xanthones to penetrate the outer membrane (OM) in Gram‐negative bacteria. The analog of α‐mangostin functionalized with amine groups crosses the OM through a self‐promoted pathway. The design and elucidation of antibacterial compounds crossing the OM facilitates the discovery of novel compounds against Gram‐negative bacterial pathogens.

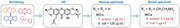

## 1 Introduction

Gram‐negative bacteria dominate the critical priority pathogens updated by WHO in 2024. Compared to Gram‐positive pathogens, the outer membrane (OM) barrier in Gram‐negative pathogens prevents the permeation of most antibacterial agents.^[^ ^1^ ^]^ In particular, the emergence of mobile colistin resistance gene *mcr*,^[^ ^2^ ^]^ encoding phosphoethanolamine transferase to modify the OM, paralyzes the last‐resort antibiotic colistin against Gram‐negative bacteria. New antibiotics are urgently needed to treat Gram‐negative bacteriaassociated infections in the clinic. The OM is predominantly composed of lipopolysaccharide (LPS), which not only stabilizes the shape and rigidity of bacteria,^[^ ^3^ ^]^ but also hinders nutrient transport and antibiotic accumulation such as hydrophobic erythromycin and rifampicin.^[^ ^4^ ^]^ Intensive attempts have been focused on targeting the biosynthesis and transportation of LPS to combat Gram‐negative pathogens.^[^ ^5^, ^6^, ^7^ ^]^ For example, the LPS translocase enzyme MsbA and LPS transport protein complex (Lpt) have emerged as innovative targets for antibiotic discovery.^[^ ^6^, ^7^ ^]^ Interestingly, such targets are structurally located either within the OM or in the periplasm space. The ability of antibacterial compounds to penetrate the OM is the prerequisite for the design of effective antibiotics against Gram‐negative pathogens.

Natural products have obtained significant attention for their accessibility, structural diversity, robust activity, and distinct modes of action.^[^ ^8^, ^9^ ^]^ The extensive chemo‐diversity of natural products provides versatile scaffolds for the discovery of antibacterial agents against resistant bacterial pathogens.^[^ ^10^ ^]^ Nevertheless, the antibacterial efficacy is predominantly restricted to Gram‐positive bacteria, owing to the formidable OM barrier in Gram‐negative bacteria impeding their permeation. With recent advances in the biological basis of bacterial OM and physicochemical characteristics of compounds against Gram‐negative bacteria, natural products‐based mimetics have been proposed to extend their antibacterial spectra.^[^ ^11^, ^12^ ^]^ The expansion of the antibacterial spectrum of natural products represents a strategic alternative in the ongoing battle against Gram‐negative pathogens, offering promising prospects for the development of effective therapies.

In this study, we first integrated a strategy combining virtual screening with phenotypic tests, to obtain natural hits. Then, we synthesized and optimized the hit to gain a lead compound (A20) through medicinal chemistry efforts. A20 penetrates the OM in a self‐promoted pathway and shows robust antibacterial activity against Gram‐negative bacteria by targeting the respiration‐related cofactor heme. The expansion and elucidation of antibacterial compounds crossing the OM facilitates the discovery of novel compounds against Gram‐negative bacterial pathogens.

## 2 Results

### 2.1 Screening of Antibacterial Hits

**Figure 1:**
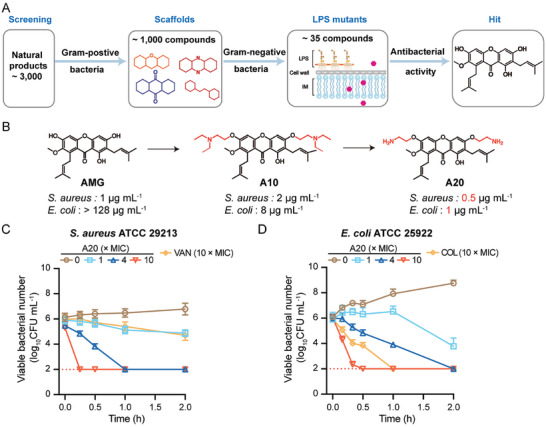
Screening of broad‐spectrum antibacterial hits. A) Scheme of the screening of antibacterial scaffolds. *S. aureus* ATCC 29213, LPS deficient *A. baumannii* 176, and LPS deficient *A. baumannii* 7‐2 were used as model strains to screen broad‐spectrum antibacterial natural products. B) Property‐guided design of antibacterial substances against Gram‐negative bacteria. Functional groups were highlighted in red. *S. aureus* ATCC 29213 and *E. coli* ATCC 2922 were used as model strains. (C,D) Time‐killing curves of A20 against C) *S. aureus* ATCC 29213 and D) *E. coli* ATCC 25922. Vancomycin (VAN) and colistin (COL) were used as controls. Experiments in B, C, and D were performed as three biologically independent experiments (*n =* 3), and the mean ± SD is shown.

Screening a broad‐spectrum antimicrobial scaffold is vital for subsequent structural optimization to cross the OM. To identify potential scaffolds, we assembled a virtual database of 3000 natural products and assessed the antibacterial activity against *Staphylococcus aureus* ATCC 29213 and *Escherichia coli* ATCC 25922 (**Figure** **1**A, Table S1, Supporting Information). Approximately 33.3% of the compounds exhibited antibacterial activities against *S. aureus*, with minimum inhibitory concentrations (MICs) lower than 32 µg mL^−1^, while no one showed activity against *E. coli* (MIC > 128 µg mL^−1^). It consists of the fact that natural products show preferred activities against Gram‐positive bacteria than Gram‐negative ones, due to the presence of intrinsic OM.^[^ ^13^, ^14^ ^]^ To test whether these compounds target the common components of bacteria, representative LPS‐deficient (ΔLPS) *Acinetobacter baumannii* was constructed.^[^ ^9^ ^]^ Encouragingly, 18 natural products displayed promising activities against ΔLPS mutants with MICs ranging from 0.125 to 32 µg mL^−1^ (Tables S2 and S3, Supporting Information), indicating their potentials as broad‐spectrum antibacterial compounds. Although these compounds display no direct antibacterial activity against *E. coli* and wild‐type *A. baumannii*, α‐mangostin (AMG) performed robust antibacterial activity with MICs of 0.125–1 µg mL^−1^ against both *S. aureus* and *A. baumannii* ΔLPS mutants. These results suggest that AMG can be used as a hit for further optimization, to enhance OM penetration.

### 2.2 Structural Optimization

Enhancing the OM penetration capacity of compounds targeting Gram‐positive bacteria through structural optimization is a critical step to expand the antibacterial spectra of AMG. Previous studies show that the hydroxyl groups at the 3rd and 6th positions of AMG are dispensable for antibacterial activity,^[^ ^9^ ^]^ and proper chemical modifications can potentiate its activity.^[^ ^15^ ^]^ Meanwhile, the LPS in OM is negatively charged at physiological pH,^[^ ^16^ ^]^ suggesting that positively charged compounds may facilitate the interaction. Given the weak charge of AMG (pKa 6.52) (Table S4, Supporting Information), we synthesized a series of AMG derivatives at the 3rd and 6th positions with the modification of hydroxyl groups (Figures S1–S28, Supporting Information), particularly the positively charged amine groups.^[^ ^17^, ^18^ ^]^ The activity of these analogs against Gram‐negative bacteria was dramatically improved by the increased alkalinity with the addition of diethylamine (A10), showing a medium MIC of 8 µg mL^−1^ against *E. coli* (**Table** **1**). The increased carbon chain length of amine‐modified analogs (A10–A12) shows constant activity against *S. aureus*, however, we observed decreased activity against *E. coli* and *Klebsiella pneumoniae*. Interestingly, the analogs with either *N*‐methyl‐n‐propylamine (A7–A9) or ethylamine (A13–A15) lost the activity against Gram‐negative bacteria, although the activities against Gram‐positive bacteria were maintained for A13–A15. These results suggest that the symmetrical structure of amine is crucial for the analogs across the OM. Hence, analogs with low steric amine groups including primary‐, secondary‐, and tertiary ones were synthesized, with diverse lengths of the carbon chain. All these analogs (A16‐A22) showed potent broad‐spectrum activities against both Gram‐positive and Gram‐negative bacteria, indicating the vital role of positive charge and steric hindrance. Remarkably, A20 displays the best broad‐spectrum activity, with the MIC ranging from 0.5–2 µg mL^−1^. Similar antibacterial phenotypes were also observed after the primary amine was replaced with cationic guanidine (A24) and amine ethylaminoformyl (A32) (Table 1).

**Table 1:** Antibacterial activities of AMG analogs.

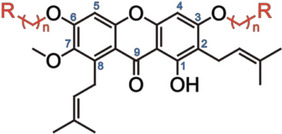						
Com.	R	n	Gram‐positive bacteria [µg mL^−1^]		Gram‐negative bacteria [µg mL^−1^]	
			*S. aureus*	*E. faecalis*	*E. coli*	*K. pneumoniae*
**AMG**	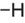	0	1	1	> 128	> 128
**A6**	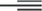	1	> 128	> 128	> 128	> 128
**A7**	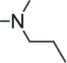	2	16	64	> 128	> 128
**A8**		3	16	32	> 128	> 128
**A9**		4	32	32	> 128	> 128
**A10**	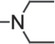	2	2	4	128	16
**A11**		3	2	1	16	32
**A12**		4	1	1	128	> 128
**A13**	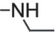	2	1	1	> 128	> 128
**A14**		3	2	1	> 128	> 128
**A15**		4	1	1	> 128	> 128
**A16**	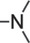	2	**2**	**1**	**2**	**4**
**A17**		3	**0.5**	**1**	**4**	**4**
**A18**		4	0.5	1	16	32
**A19**	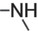	2	**0.5**	**0.5**	**1**	**4**
**A20**	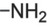	2	**0.5**	**0.5**	**1**	**2**
**A21**		3	1	0.5	2	2
**A22**		4	1	1	8	8
**A23**	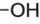	2	> 128	> 128	> 128	> 128
**A24**	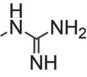	2	**0.5**	**0.5**	**2**	**4**
**A32**	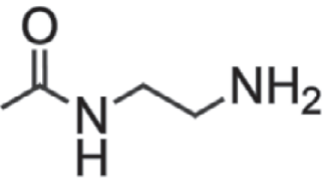	1	**2**	**1**	**2**	**2**
**GEN**			0.5	1	1	4
**DOX**			0.25	1	2	8

*S. aureus*, *Staphylococcus aureus* ATCC 29213; *E. faecalis*, *Enterococcus faecalis* ATCC 29212; *E. coli*, *Escherichia coli* ATCC 25922; *K. pneumoniae*, *Klebsiella pneumoniae* ATCC 43816. GEN, gentamicin; DOX, doxycycline.

To further assess the generality of our structural optimization for other natural products, scaffolds that share a tricyclic skeleton similar to the xanthone like AMG, were tested for the structure‐activity relationship analysis to obtain more broad‐spectrum antibiotics. Unfortunately, no broad‐spectrum antibacterial derivates of phenazine‐1‐carboxylic acid (PCA) (Table S5, Supporting Information) and rhein (RHE) (Table S6, Supporting Information) were found, indicating that the modification with amine groups to broaden the antibacterial spectrum is scaffold unique, and the activity against Gram‐positive bacteria is a prerequisite for the propose of interest. Taken together, the transmembrane ability of AMG analogs is synergistically modulated by positive charges and steric hindrance at the 3rd and 6th positions of the proper core skeleton.

### 2.3 A20 is a Potent Antibacterial Candidate

Considering the potent broad‐spectrum activity of the six analogs A16, A17, A19, A20, A24, and A32 (Table 1), the antibacterial activities of these compounds were further determined against a large panel of clinical isolates (*n =* 160). The antibacterial potency of these analogs was consistent against Gram‐positive bacteria, with MIC_50_ of 1–2 µg mL^−1^ (Table S7, Supporting Information). Notably, A19 and A20 exhibited exceptional activities against methicillin‐resistant *S. aureus* (MRSA), with MICs ranging from 0.5 to 2 µg mL^−1^, superior to gentamicin and doxycycline (Table S8, Supporting Information). The susceptibility of these analogs against 120 clinical Gram‐negative pathogens, including *A. baumannii, Aeromonas spp*., *E. coli*, *Haemophilus parasuis*, *K. pneumoniae*, *Pseudomonas aeruginosa, Pasteurella multocid*, and *Salmonella spp*. was conducted, with MIC_50_ of 2–4 µg mL^−1^ (Table S7, Supporting Information). Among these analogs, A20 displayed comparable activity with colistin. Remarkably, A20 showed considerable activities against MDR *E. coli* harboring *mcr* and *bla*_NDM_, with MICs of 2–4 µg mL^−1^, suggesting a promising resilience against the emerging drug resistance. In addition, we evaluated the safety of A20 and five analogs using non‐tumor cell lines including Vero, HaCaT, RAW264.7, IEC6, and HepG2 cell lines. A20 showed half‐maximal inhibitory concentrations (IC_50_) of 4.10 to 13.01 µg mL^−1^ (Table S9, Supporting Information), with appropriate therapeutic indexes (TI = IC_50_/MIC_50_ > 3), superior to other analogs (Table S10, Supporting Information). These results indicate that A20 has therapeutic potential. Taken together, our results indicate that A20 is a potent broad‐spectrum antibacterial compound to combat MDR bacteria, practically against Gram‐negative pathogens.

To comprehensively assess the antimicrobial potency of A20, the time‐killing dynamics were explored. Interestingly, A20 revealed rapid bactericidal activities against both *S. aureus* (Figure 1C) and *E* *. coli* (Figure 1D) in a dose‐dependent manner. A20 could promptly reduce the viable bacteria below the limit of detection in less than 20 min at high levels (10 × MIC), surpassing vancomycin and colistin. In addition, no de novo resistance to A20 was observed during a 60‐day serial passage of *S. aureus* ATCC 29213 (Figure S29A, Supporting Information) and *E. coli* ATCC 25922 (Figure S29B, Supporting Information). These results suggest that A20 is a promising broad‐spectrum antibiotic candidate.

### 2.4 Self‐Promoted Uptake Pathway

**Figure 2:**
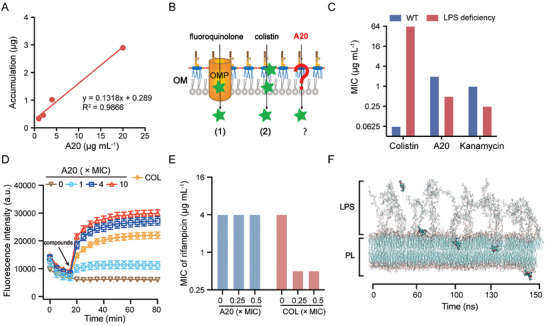
Self‐promoted uptake of A20 for transmembrane transportation. A) Intracellular accumulation kinetics of A20 in *E. coli*. B) Scheme of antibacterial agents crosses the OM. 1) OMP‐depedent fluoquinolones and 2) LPS‐dependent colistin. C) MICs of antibacterial compounds against ΔLPS‐ mutants. WT, wild‐type. D) Competitive binding assay between A20 and the fluorescent probe BODIPY‐TR‐cadaverine in the presence of lipid A. E) Synergy of A20 and colistin with hydrophobic rifampicin. F) Simulation of A20 interacting with OM in *E. coli*. Experiments in A, C, D, and E were performed as three biologically independent experiments (*n =* 3), and mean ± SD is shown.

Given the broad‐spectrum bactericidal efficacy of A20 particularly for Gram‐negative bacteria, we proposed that A20 should effectively penetrate the OM. To quantify the intracellular accumulation of A20, we explored the penetration dynamics by assessing the whole‐cell accumulation of A20 in *E. coli* ATCC 25922. First, we established a liquid chromatography‐mass spectrometry (LC‐MS) method and found the accumulation curve of A20 followed by a linear regression (*R* ^2^ = 0.9866) (**Figure** **2**A). A20 exhibited both time‐ and dose‐dependent accumulation, with a penetration rate of ≈ 0.011 pmol 2 × 10^9^ cells^−1^ min^−1^ (Figure S30A, Supporting Information). This process aligns with the conventional non‐specific uptake pathway (Figure 2B), where passive diffusion via OM proteins (OMPs) is a classic route for the transportation of certain antibiotics,^[^ ^19^, ^20^ ^]^ particularly for hydrophobic fluoroquinolones and cephalosporins.^[^ ^21^, ^22^ ^]^ Considering the addition of amines into A20 enhanced its hydrophilicity, with the decreased cLogD (pH = 7.4) from 3.30 (AMG) to 2.79 (A20), we then investigated whether A20 penetrates through OMPs. OmpF is a major porin in Gram‐negative bacteria for the transportation of hydrophilic and cationic molecules.^[^ ^20^ ^]^ Thus, we compared the antibacterial activity of A20 against wild‐type *E. coli* and OmpF knock‐out mutants^[^ ^23^ ^]^ to explore whether A20 relies on OMPs. A20 showed comparable activity (MIC = 4 µg mL^−1^) against both the wild‐type and mutants (Table S11, Supporting Information). In contrast, the activity of typical porin‐dependent antibiotics such as ciprofloxacin, an example of fluoroquinolones, and cefoxitin, an example of β‐lactams, against the mutant was four‐fold lower than the wild‐type. The MICs increased from 0.125 to 0.5 µg mL^−1^ for cefoxitin, and from 0.0078 to 0.03125 µg mL^−1^ for ciprofloxacin against the wild‐type and mutants, respectively. These results indicate that A20 acts like porin‐independent antibiotics.

Direct interaction with LPS, the major component of OM, has been considered an effective strategy to overcome the OM barrier.^[^ ^24^, ^25^ ^]^ Given that the additional positively charged amines in A20 potentiated the interaction with anionic LPS through the electrostatic effect, we evaluated the role of LPS in the transportation of A20. We determined the antibacterial activity of A20 against LPS‐deficient strains. Compared to the reduced activity of colistin against *A. baumannii* ΔLPS mutants, A20 displayed mildly enhanced activity against these mutants (Figure 2C), with the MICs decreasing from 2–4 µg mL^−1^ of the wild‐type to 0.5–1 µg mL^−1^ of the mutants. These results are similar to the “self‐promoted” uptake aminoglycosides (e.g. kanamycin), which penetrate the OM by electrostatic binding to LPS via amine groups.^[^ ^26^ ^]^ Consistently, there was no statistically significant difference in the accumulation of A20 between the wild‐type and mutants (Figure S30B, Supporting Information). These results indicate that A20 interacts with LPS across the OM. Hence, we characterized the interaction between A20 and LPS. Exogenous addition of purified LPS (64 µg mL^−1^) showed slight inhibition on the antibacterial activity of A20 against *E. coli* (Figure S30C, Supporting Information). Meanwhile, the exogenous addition of Mg^2+^, competing for the negatively charged phosphate (PO_4_^3−^) of lipid A in LPS, suppressed the activity of A20 with the increased MIC of eightfold (Figure S30D, Supporting Information). These results suggest the critical role of electrostatic interaction between A20 and LPS. Furthermore, a lipid A displacement assay was performed between A20 and the fluorescent probe BODIPY‐TR‐cadaverine (BC), which binds to free lipid A resulting in fluorescence quenching. Expectedly, the fluorescence of BC increased instantly in the presence of A20 (Figure 2D), demonstrating that A20 competitively binds to lipid A. Then, we evaluate the subsequent event of OM in *E. coli* treated with A20. Compared to the typical OM disruptor colistin, A20 induced weak disturbance of OM permeability (Figure S31A, Supporting Information). Consequently, no synergies were found between A20 and hydrophobic antibiotics such as rifampicin and erythromycin (Figure 2E; Figure S31B,C, Supporting Information). Nevertheless, the antibacterial activity of A20 was improved under either colistin (Figure S31D, Supporting Information) or its analog polymyxin B nonapeptide (PMBN) (Figure S31E, Supporting Information). These results demonstrate that A20 penetrates OM through a “self‐promoted” pathway. To visualize the transmembrane dynamics, a molecular dynamics simulation was performed. It showed that A20 first attached the OM by electrostatic interaction between the amine groups of A20 and hydrophilic heads of LPS (Figure 2F), then anchored into the membrane interior via hydrophobic interaction between the isopentenyl groups of A20 and hydrophobic fatty acid tails of LPS. After 150 ns, A20 went across the OM thoroughly. Collectively, A20 is prone to enter the OM by a self‐promoted uptake pathway through the electrostatic and hydrophobic interactions with LPS.

### 2.5 Phospholipid‐Dependent Intracellular Accumulation

**Figure 3:**
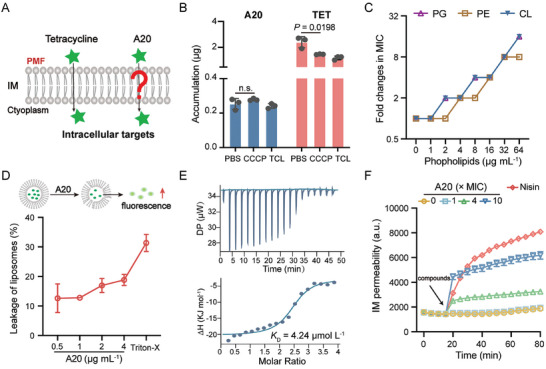
A20 binds to phospholipids to cross the inner membrane. A) Schematic diagram of chemicals crossing the inner membrane. B) Intracellular accumulation of A20 under the treatment of PMF disruptors including 3‐chlorophenylhydrazone (CCCP) and triclosan (TCL). C) Fold changes in MICs of A20 in the presence of bacterial phospholipids including phosphatidylethanolamine (PE), phosphatidylglycerol (PG), and cardiolipin (CL). D) Leakage of PG liposomes under the treatment of A20 for 10 min. E) The affinity between A20 and PG based on the isothermal titration calorimetry test. F) The permeability of the inner membrane probed with propidium iodide (PI) for *E. coli* under the treatment of A20. Experiments in A, C, D, and E were performed as three biologically independent experiments (*n =* 3), and mean ± SD is shown. *P* values were determined using the One‐way ANOVA test, and *P* < 0.05 was considered statistically significant.

After crossing the OM, antibacterial substances need to overcome the inner membrane (IM) to access intracellular targets (**Figure** **3**A). Proton motive force (PMF) plays a pivotal role in IM penetration for antibiotics, including tetracyclines and aminoglycosides.^[^ ^27^ ^]^ Notably, unlike tetracyclines that target bacterial intracellular ribosomes, the intracellular level of A20 was much lower (≈ 0.24 µg) than tetracyclines (≈ 2.36 µg) (Figure 3B), suggesting that A20 probably binds to membrane components to cause bacterial death. To assess the effect of PMF on intracellular accumulation of A20, we evaluated the antibacterial activity of A20 under the treatment of carbonyl cyanide 3‐chlorophenylhydrazone (CCCP), a classic PMF disruptor.^[^ ^28^ ^]^ CCCP had no effects on the antibacterial activity of A20 against *E. coli* (Figure S32A, Supporting Information), and the accumulation of A20 was parallel to the untreated (Figure 3B). However, the intracellular tetracycline was significantly decreased (*P* = 0.0198) following CCCP treatment. Similar trends were also observed in the presence of triclosan (TCL) (Figure 3B), another PMF disruptor.^[^ ^29^ ^]^ To further clarify the role of PMF, intracellular accumulation of A20 was quantified in other Gram‐negative bacteria. The intracellular accumulation of A20 was comparable with or without the treatment of CCCP in both *K. pneumoniae* and *A. baumannii* (Figure S32B, Supporting Information), demonstrating the accumulation of A20 was not associated with PMF. Intracellular drug accumulation is related not only to the entry but also to the efflux mechanism.^[^ ^30^ ^]^ To further exclude the effect of PMF on intracellular accumulation, we evaluated the efflux of A20 in Gram‐negative bacteria. The residual A20 in bacteria was quantified using our established LC‐MS methods after incubation in PBS (Figure S33A, Supporting Information). No efflux of A20 was detected in *E. coli*, *A. baumannii*, and *K. pneumoniae* (Figure S33B,C, Supporting Information). Taken together, these findings demonstrate that the intracellular accumulation of A20 is independent of PMF.

Bacterial IM is composed of phospholipids, similar to the inner leaflet of the OM.^[^ ^31^ ^]^ Considering that A20 penetrates OM thoroughly (Figure 1F), we hypothesized that A20 interacts with phospholipids to cross bacterial IM (Figure 3A). First, we compared the antibacterial activity of A20 in the presence of three typical kinds of phospholipids derived from the membrane components of Gram‐negative bacteria, including phosphatidylethanolamine (PE), phosphatidylglycerol (PG), and cardiolipin (CL). All these phospholipids inhibited the antibacterial activity of A20 in a dose‐dependent manner (Figure 3C), especially anionic PG and CL. To further evaluate the interaction between A20 and phospholipids, we synthesized PG‐containing liposomes loading 5‐carboxyfluorescein (CF) as an indicator. CF in PG liposomes leaked quickly in a dose‐dependent manner after the treatment of A20 for 10 min (Figure 3D), demonstrating the direct interaction between A20 and PG. Additionally, specific binding between A20 and PG was confirmed based on isothermal titration calorimetry (ITC) tests. The equilibrium dissociation constant (*K*_D_) between A20 and PG was 4.24 µmol L^−1^ (Figure 3E). Interestingly, both Δ*H* (−17.6 KJ mol^−1^) and ‐*T*Δ*S* (−13.2 KJ mol^−1^) dominate A20 binding to PG, suggesting that electrostatic and hydrophobic effects together drive the interactions. The phospholipids such as PG in the IM are important to maintain the physical structure and physiological functions of bacteria.^[^ ^32^ ^]^ Last, we determined the biophysical properties of IM in the presence of A20. IM permeability increased after *E. coli* treated with A20 at high levels (4–10 × MIC) (Figure 3F), while the membrane fluidity was invariable (Figure S34, Supporting Information), indicating that A20 penetrates the IM without causing obvious physical disruption. Taken together, A20 is a promising lead that targets Gram‐negative bacteria through interacting with membrane phospholipids.

### 2.6 Antibacterial Target

**Figure 4:**
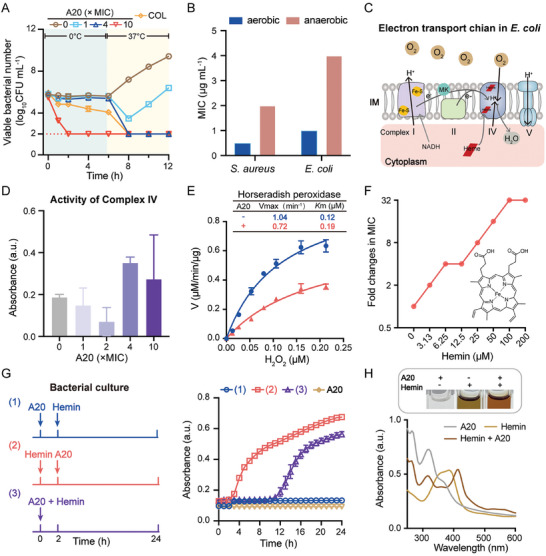
A20 disrupts bacterial respiration. A) Metabolism‐dependent bactericidal activities of A20 against *E. coli*. B) Antibacterial activities of A20 under aerobic and anaerobic conditions. C) Scheme of the respiratory chain in *E. coli*. D) Activity of respiratory complex IV in *E. coli* treated with A20. E) Enzyme activity of horseradish catalase in the presence of A20. F) MICs of A20 with exogenous hemin. G) Scheme of the experimental design and growth curves of *E. coli* under the treatment of A20 and hemin. H) The representative color and the UV−Vis spectra of hemin (20 µM) with A20 in HEPES (200 mM, pH 7.0). Experiments were performed as three biologically independent experiments (*n =* 3). Data are presented as mean ± SD.

To dissect the underlying mechanism of A20, we first explored the time‐kill dynamics of A20 against metabolically active and inactive bacteria. A20 revealed rapid bactericidal activities in a dose‐dependent manner against metabolically active *E. coli* at 37 °C (**Figure** **4**A), while displaying no or less bactericidal activities against metabolism‐restricted *E. coli* at 0 °C. These results indicate that the mode of action (MOA) of A20 is closely related to bacterial metabolism. Nucleotide biosynthesis such as DNA replication is the fundamental metabolism in bacteria.^[^ ^33^ ^]^ We found A20 at high concentration (10 × MIC, 10 µg mL^−1^) led to low migration or tailed bands of DNA extracted from *E. coli* ATCC 25922 in the agarose gel (Figure S35A, Supporting Information) and a slight decrease of fluorescence intensity of propidium‐iodide (PI) after incubation for 1 h (Figure S35B, Supporting Information). These results suggest that A20 is not prone to bind to DNA. In addition, bacterial respiration, especially aerobic respiration is a metabolic activity for bacterial growth and survival.^[^ ^34^ ^]^ To test whether A20 interferes with bacterial respiration, we assessed the antibacterial activity of A20 under aerobic and anaerobic conditions. The antibacterial activity of A20 against both *E. coli* and *S. aureus* was two‐ to four‐fold lower under anaerobic conditions than under aerobic conditions, with decreased MICs from 0.5–1 to 2–4 µg mL^−1^ (Figure 4B). These observations suggest that A20 may effectively inhibit aerobic respiration. Bacterial aerobic respiration produces protons and transfers them to the electron acceptor oxygen (Figure 4C). The reduction of oxygen is mainly catalyzed by the respiratory complex IV in *E. coli*.^[^ ^34^ ^]^ Consistently, the activity of complex IV was disrupted in the presence of A20 at high levels (Figure 4D). One product ΔpH, a main contributor of PMF, dissipated in a dose‐dependent manner under the treatment of A20 (Figure S36, Supporting Information), indicating that A20 disrupts bacterial redox balance. To mimic the redox function of complex IV,^[^ ^35^ ^]^ the activity of peroxidase horseradish was tested based on the Michaelis‐Menten equation.^[^ ^36^ ^]^ Compared to the enzymatic activity of maximum reaction rate (*V*_max_ = 1.04 min^−1^) and Michaelis constant (*K*_M_ = 0.12 µM) in the absence of A20, whereas *V*_max_ was reduced to 0.72 min^−1^ and the *K*_M_ kept at 0.19 µM in the presence of A20 (Figure 4E). These parameters indicate that A20 is a noncompetitive inhibitor of peroxidases, by inhibiting the activity of enzymes while maintaining the binding affinity.^[^ ^37^ ^]^

The activity of classic peroxidases such as respiratory complex IV and horseradish peroxidase need hemes as cofactors.^[^ ^38^ ^]^ To assess the role of hemes on the MOA of A20, the oxidation state of heme, hemin, was used. Exogenous hemin resulted in a 32‐fold reduction of the antibacterial activity of A20 (Figure 4F), with the MIC increasing from 1 to 32 µg mL^−1^. Then, we tested the bacterial growth curves under the treatment of A20, hemin, and both thereof (Figure 4G). Compared to the pretreatment of A20, the simultaneous addition of both, pre‐treatment of hemin endowed bacteria with the possibility to tolerate A20 (Figure S37, Supporting Information), resulting in bacterial survival under the treatment of A20 at bactericidal level (2 × MIC). These results demonstrate that hemin antagonizes the antibacterial activity of A20. To validate the findings, we further examined whether A20 directly interacts with hemin based on UV–Vis spectrum analysis. After the addition of A20, the Soret absorption band of hemin with the typical peak at 395 nm was blue‐shifted to 380 nm, and displayed a new peak at 415 nm (Figure 4H), with a color change from yellow‐green to reddish‐brown. These observations are similar to previously reported hemin‐binding substances,^[^ ^38^ ^]^ demonstrating the presence of low‐spin hemin and the formation of A20‐hemin complexes. Given that hemin is an iron‐containing porphyrin,^[^ ^39^ ^]^ iron‐chelated and iron‐free hemin precursors were further used to dissect the interaction between A20 and hemin. No changes in the spectra were observed for protoporphyrin IX, the iron‐free hemin precursor, in the presence of A20 (Figure S38A, Supporting Information). Moreover, either the reduced or oxidized forms of iron showed slight inhibition on the activity of A20 (Figure S38B, Supporting Information). These results confirm that A20 binds to hemin. This observation is consistent with the result that A20 tends to accumulate in the membrane not the cytoplasm of bacteria (Figure 3B). Last, we quantified the intracellular hemin intermediates in *E. coli* using fluorescent probes. The porphyrins increased under the treatment of A20 (Figure S38C, Supporting Information), indicating that A20 causes the disruption of hemin. Taken together, the lead A20 entries Gram‐negative bacteria through a self‐promoted uptake pathway and kills bacteria by targeting the cofactor heme of complex IV, to inhibit bacterial respiratory.

### 2.7 Efficacy of A20 In Vivo

**Figure 5:**
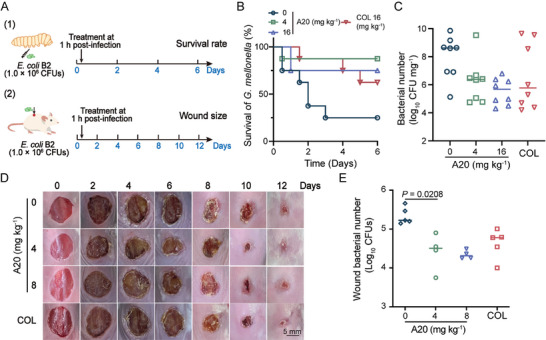
Therapeutic efficacy of A20 in vivo. A) Scheme of the experimental protocol for the *G alleria mellonella* infection model and mouse skin wound infection model. B) Time‐survival curves of *G. mellonella* larva during six days after infection (*n =* 8). C) Bacterial loads in *G. mellonella* at 6 days post‐infection (*n =* 8). Data are representative of eight independent experiments and values are expressed in mean ± SEM. D) Representative pictures of wound healing records. E) Bacterial loads in wounds on day 12. Data are presented as mean ± SD. *P* values were determined using an unpaired, two tailed Stuent's *t*‐test.

Inspired by the promising antibacterial activity of A20 in vitro, we further assessed its therapeutic efficacy in vivo. First, two animal models infected with MDR *E. coli* B2 were established (**Figure** **5**A). *Galleria mellonella* were infected with a lethal dose of *E. coli* B2. After 1 h post‐infection, A20 was administrated at a range of doses from 0 to 16 mg kg^−1^. At 6 days after infection, the larva treated with A20 showed strikingly increased survival rates (Figure 5B). A20 at a dose of 4 mg kg^−1^ effectively protected *G. mellonella* from infection, with survival increased from 25% to 87.5%. Meanwhile, the bacterial loads in larvae were significantly decreased after the treatment of A20 (Figure 5C). In the mouse wound infection model, the morphology and size of the wound displayed better cure trends under the treatment of 4 mg kg^−1^ A20 (Figure 5D). Consistently, the bacterial burden in the wounds was reduced significantly under the treatment of A20, with more than ten‐fold reduction of bacterial numbers after 12 days (Figure 5E). These results indicate the potential of A20 as an antibiotic candidate to combat clinical pathogens in vivo.

## 3 Discussion

The OM of Gram‐negative bacteria paralyzes most hydrophobic antibiotics that are effective against Gram‐positive bacteria.^[^ ^40^, ^41^ ^]^ Optimizing the structure of these hydrophobic antibiotics to facilitate OM penetration is an alternative strategy to develop broad‐spectrum antibacterial agents. In this study, we screened potential broad‐spectrum antibacterial scaffolds from natural products and optimized the structure of hits such as AMG to facilitate OM transportation. Based on the SAR analysis, amine modification with low steric hindrance at the 3rd and 6th positions of AMG expands its activity against Gram‐negative bacteria. Among the active compounds, six analogs show superior activity to the broad‐spectrum antibiotics, including aminoglycosides and tetracyclines. Notably, candidate A20 exerts robust antibacterial activity against MDR bacteria with MIC_50_ of 2 µg mL^−1^, comparable to lolamicin^[^ ^42^ ^]^ and MCP,^[^ ^1^ ^]^ novel agents targeting Gram‐negative bacteria. These results demonstrate that the structural optimization of natural products is a promising strategy for discovering broad‐spectrum antibacterial agents.

Targeting LPS, the major component in the OM, is an effective way to kill bacteria.^[^ ^25^ ^]^ For example, the routine antibiotic colistin binds to LPS.^[^ ^43^ ^]^ The antimicrobial peptide thanatin shows competitive replacement of divalent cations that consolidate LPS,^[^ ^44^ ^]^ leading to OM disruption. In our study, candidate A20 crosses the OM via a self‐promoted pathway. Similar to aminoglycosides with polyamines such as kanamycin, A20 containing two amine groups compete with divalent cations to bind to the phosphate groups of lipid A in LPS, facilitating transmembrane transport across the OM. Although A20 could not increase the OM permeability and enhance the activity of other hydrophobic antibiotics, such transmembrane pathway has less potential to develop antibiotic resistance, as it induces minimal selective pressure on bacterial OM. The underlying mechanism of the self‐promoted pathway remains elusive, and more works are needed to decipher the interaction between A20 and LPS, particularly the dynamics of the self‐promoted pathway, to facilitate the discovery of novel antibacterial agents. Notably, compared with the well‐known self‐promoted antibiotics, such as kanamycin, A20 exerts rapid bactericidal activity against MDR bacteria. Consequently, these unique properties endow A20 with a compelling candidate for further exploration and development as a novel antibiotic, particularly against MDR Gram‐negative bacterial pathogens.

Although we have demonstrated the role of amine groups in facilitating OM penetration in AMG, such modification is scaffold‐restricted, enervating the generality to other tricyclic substances such as PCA and RHE (Tables S2 and S3, Supporting Information). Additionally, the modification may not be robust in overcoming the OM barrier in *P. aeruginosa* (Table S7, Supporting Information), a notorious Gram‐negative pathogen expressing diverse efflux pumps and lacking non‐specific porins. These limitations underscore the necessity for a more extensive structural optimization to enhance the versatility and success rate for the discovery of broad‐spectrum antimicrobial agents.

In summary, we show that the modification of amine groups to natural products facilitates OM transportation in a self‐promoted pathway, enabling broad‐spectrum antibacterial activity. A20 is a promising candidate for the development of novel antibiotic agents to combat MDR bacterial pathogens‐associated infections. The distinctive transmembrane pathway not only enhances its antibacterial efficacy but also mitigates potential risks associated with the development of drug resistance.

## 4 Experimental Section

### Bacterial Strains and Culture Media

The bacterial strains used in this study are listed in Table S1, Supporting Information. Routine propagation of bacteria was conducted in Brain Heart Infusion (BHI) broth at 37 °C aerobically.

### Cell Lines and Cell Culture

The cell lines used in this study included Vero, HaCaT, RAW264.7, IEC6, and HepG2. These cells were cultured in Dulbecco's Modified Eagle Medium (DMEM, Gibco, Thermo Fisher Scientific) supplemented with 10% fetal bovine serum (FBS, Gibco, Life Technologies) and 1% penicillin‐streptomycin (Gibco, Life Technologies). Cells were maintained under standard incubation conditions of 5% CO_2_ and 37 °C. Passaging was performed every 12 to 24 h, and cells in the exponential growth phase were used for further experiments.

### Mice

Female BALB/c aged 6–8 weeks were obtained from the SPF (Beijing) Biotechnology Co. Ltd. Mice were adapted to standardized environmental conditions (temperature = 23 ± 2 °C; humidity = 55 ± 10%) for 1 week before infection. The animal study protocols were performed in accordance with the relevant guidelines and regulations (ID: SKLAB‐B‐2010‐003). The laboratory animal usage license (SYXK‐2021‐0012) was certified by the Beijing Association for Science and Technology. The experimental protocols were approved by the Laboratory Animal Ethics Committee of China Agricultural University (AW81703202‐2‐1).

### Synthetic Compounds

Reagents for chemical synthesis were purchased from Shanghai Titan Scientific Co., Ltd unless otherwise indicated. Details on the synthesis and characterization of AMG derivatives in this work were provided in the Supporting Information.

### Antibacterial Assay

The antibacterial assay was performed by the micro‐broth dilution method according to Clinical and Laboratory Standards Institute (CLSI) 2022 guidelines.^[^ ^45^ ^]^ Bacteria were normalized to an optical density of 10^6^ colony‐forming units (CFUs) mL^−1^ and the antibacterial activities of the synthesized compound were determined in cation‐adjusted Mueller‐Hinton broth (MHB‐II) (Shanghai Canspec, Cat No. BD‐212322). After incubation at 37 °C for 16–18 h, the minimum inhibitory concentrations (MICs) were defined as the lowest concentrations of compounds with no visible growth of bacteria.

### Chequerboard Study

Fractional inhibitory concentrations (FICs) were determined using chequerboard assays based on previous studies.^[^ ^46^, ^47^, ^48^ ^]^ Various compounds or antibiotics were added along the abscissa and A20/colistin was diluted along the ordinate. Overnight bacterial culture normalized to an optical density of 10^6^ CFUs mL^−1^ were then added. FIC indices were calculated according to FICI = (C_A_/MIC_A_) *+* (C_B_/MIC_B_)*, in* which C_A_ was the MIC of compound A in combination with compound B and C_B_ was the MIC of compound B in combination with compound A; MIC_A_ and MIC_B_ were the MIC of compound A and compound B alone. Synergy was defined as FICI ≤ 0.5, and antagonism was defined as FICI > 4.0. MgCl_2_ (Shanghai Titan, Cat No. 01115966), CaCl_2_ (Shanghai Titan, Cat No. 011487970), FeCl_3_ (Sigma‐Aldrich, Cat No. 157740), FeSO_4_ (Sigma‐Aldrich, Cat No. F7002), LPS (Sigma‐Aldrich, Cat No. L2880) and hemin (MCE, HY‐19424) were added to the broth medium to test the effect on the activity of A20.

### Growth Curve

The growth curves of *E. coli* were determined by measuring the optical density (OD_600_ _nm_). Overnight cultures of *E. coli* were diluted to 10^6^ CFUs mL^−1^ into MHB‐II medium and were mixed with an equal volume of A20 in a 96‐well microplate. The OD_600_ _nm_ values of the culture were measured with an interval of one hour incubated at 37 °C, using a microplate reader (Tecan Infinite 200 pro, Tecan, Switzerland).

### Time‐Killing Assay

Bacteria at exponential phase (10^6^–10^7^ CFUs mL^−1^) were challenged with A20 (1 ×, 4 ×, 10 × MIC) at 37 °C with shaking at 200 rpm. 100 µL aliquots of bacterial suspensions were serially diluted and plated on MH agar plates. The CFUs were counted after incubation at 37 °C for 16 h. The bactericidal kinetics 0 °C was performed as above with minor modification.^[^ ^49^ ^]^ The bacterial suspension was precooled in an ice bath and the samples were incubated on ice with shaking throughout the experiment.

### Cytotoxicity Assay

The cells were seeded into 96‐well plates at a density of 8,000 cells per well and allowed to adhere overnight in DMEM supplemented with 10% FBS. The cells were treated with A20, ranging from 0.25 to 128 µg mL^−1^ (*n =* 4 per group). DMEM with 10% FBS but no A20 treatment was used as a negative control group, and DMEM with 10% FBS but without cells were used as a blank control group. After 24 h of incubation, morphological changes in the cells were observed using an inverted microscope (Leica, Germany). The CCK‐8 assay was then carried out, and the OD of each well was measured at a wavelength of 450 nm. Cell viability was calculated using the following equation:

(1) $$\begin{array}{cc} \mathrm{Cell}\;\mathrm{viability}\left(\%\right) & =\left(OD_{experiment}-OD_{blank}\right)/\left(OD_{negative\;control}-OD_{blank}\right)\\ & \;\times 100 \end{array}$$

### Antibiotic Accumulation Assay

The accumulation of intracellular antibiotics in bacteria was determined based on the established antibiotics extraction method.^[^ ^29^ ^]^ The quantification of intracellular A20 was determined by liquid chromatography‐mass spectrometry (LC‐MS) analysis (Shimadzu LC‐MS 8045). An extracted ion chromatogram of A20 was obtained in positive ESI mode at a retention time of 4.45 min. Elucidation of A20 based on the product ions of m/z 497.35 > 355.05, m/z 497.35 > 398.15, and m/z 497.35 > 381.20.

### Outer Membrane Permeability Assay

1‐*N*‐phenylnaphthylamine (NPN, Sigma‐Aldrich, Cat No. 104043) (10 µM) with an excitation wavelength of 350 nm and emission wavelength of 420 nm was used to evaluate the outer membrane permeability.^[^ ^50^, ^51^ ^]^

### Inner Membrane Permeability Assay

According to previous studies, bacterial inner membrane permeability was determined using the fluorescent probe propidium iodide (PI, Sigma‐Aldrich, Cat No. 537059).^[^ ^52^ ^]^ The measurement was recorded at the excitation wavelength of 540 nm and emission wavelength of 610 nm.

### Membrane Fluidity Assay

Laurdan (MCE, Cat No. HY‐D0080) determined the membrane fluidity as previously described.^[^ ^9^, ^53^ ^]^ Benzyl alcohol (50 mM) was used as a positive control. The fluorescence intensities of Laurdan were measured with excitation wavelength at 460 nm and emission wavelength at 500 nm. Laurdan generalized polarization (GP) was calculated using the following formula:

(2) $$\mathrm{GP}=\left(I_{460-}I_{500}\right)/\left(I_{460}+I_{500}\right)$$

### ΔpH Measurement

The ΔpH of the membrane was assessed using pH‐sensitive fluorescence probe BCECF‐AM (MCE, Cat No. HY‐101883) (10 µM) with an excitation wavelength of 488 nm and emission wavelength of 535 nm.^[^ ^9^ ^]^

### Lipid A Competition Assay

Lipid A competition assay was investigated using the fluorescent probe BODIPY‐TR‐cadaverine (BC, MCE, Cat No. HY‐D1594), which exhibits fluorescence dequenching upon binding.^[^ ^54^, ^55^ ^]^ Briefly, BC (final concentration of 5 µM) and lipid A (Sigma‐Aldrich, Cat No. L5399) (final concentration of 3.5 µg mL^−1^) were mixed and incubated for 15 min in the dark at room temperature. Subsequently, an equal volume of tested compounds was added to compete with BC for binding to lipid A. The fluorescence intensities were measured at the excitation wavelength of 580 nm and emission wavelength of 620 nm, using the Infinite M200 Microplate reader (Tecan).

### Isothermal Titration Calorimetry (ITC) Assay

The interaction between A20 and PG (Sigma–Aldrich, Cat No. 63371) was performed using ITC analysis at 25 °C by Microcal high‐sensitivity ITC calorimeter (Malvern). PG (2 mM) was titrated into the sample cell containing A20 (0.1 mM) in HEPES (20 mM, pH 7.0) buffer. The obtained data were processed using the software to calculate the equilibrium dissociation constant (*K_D_*), stoichiometry (*n*), and the change of enthalpy (Δ*H*) and entropy (Δ*S*).

### Liposome Leakage Assay

The liposomes were prepared using the thin‐film hydration method followed by extrusion.^[^ ^56^, ^57^ ^]^ Briefly, PG (10 mg) in chloroform‐methyl alcohol (V/V, 1:1) was evaporated under a gentle stream of argon. The residual film was hydrated with a 70 mM 5‐carboxyfluorescein (CF) solution. The suspension was then freeze‐thawed for five cycles and successively extruded through polycarbonate filters. CF‐entrapped large unilamellar vesicles (LUVs) were separated from free CF on a Sephadex G‐50 column. The LUVs were diluted with PBS and mixed with A20 to evaluate the liposome leakage ability for A20. The release of CF from LUVs was monitored at an excitation wavelength of 492 nm and an emission wavelength of 518 nm. The relative leakage percentage was calculated according to the following formula:

(3) $$\mathrm{Leakage}\left(\%\right)=\left[I_{\left(test\right)}-I_{\left(control\right)}\right]/I_{\left(control\right)}\times 100\%$$

### All‐Atom Molecular Dynamics (MD) Simulations

All‐atom MD simulations were performed in the GROMACS (version 2020.6) simulation package using the CHARMM 36 force field and the TIP3P water model.^[^ ^58^ ^]^ The simulated *E. coli* membrane contained 136 PE and 5 LPS lipids. Following equilibration, the MD simulation was conducted for 150 ns under the NPT ensemble conditions. An integration time step of 1 fs was used and the temperature was coupled to 298 K using the Nose‐Hoover method. A cutoff scheme of 1.2 nm was used for the non‐bond interactions, and the Particle Mesh Ewald method with a Fourier spacing of 0.1 nm was applied for the long‐range electrostatic interactions. All covalent bonds with hydrogen atoms were constrained using the LINCS algorithm.

### Gel Retardation Assay

A 1% agarose gel containing DoodView dye was prepared, and genomic DNA samples of *E. coli* ATCC 25922 (40 µg mL^−1^, extracted using spin‐column method) were mixed with A20 at 0 ×, 1 ×, 2 ×, 4 ×, and 10 × MIC (MIC = 1 µg mL^−1^), as well as kanamycin, followed by incubation at 37 °C for half an hour. After adding the loading buffer, the samples and marker were loaded into the gel, and electrophoresis was performed at 130 V for 40 min. DNA band shifts were visualized under UV light and image.

### PI Displacement Assay

The extracted genomic DNA (10 ng µL^−1^) was mixed with PI (1 µg mL^−1^) in PBS buffer (pH 7.4) and incubated at 37 °C for half an hour. After incubation, the DNA‐dye mixture was aliquoted into 2 mL EP tubes, and varying concentrations of A20 at 0 ×, 1 ×, 2 ×, 4 ×, and 10 × MIC (MIC = 1 µg mL^−1^) were added. The mixtures were further incubated at 37 °C for another half an hour to reach equilibrium. The fluorescence intensity was monitored using the Tecan Infinite 200 microplate reader. All experiments were conducted in triplicate.

### UV–Vis Spectrum Assay

UV‐Vis spectra between A20 and hemin assay were performed in a quartz 96‐well flat bottom plate according to a previous study.^[^ ^38^ ^]^ Hemin (20 µM of final concentration) was incubated with various concentrations of A20 in buffer (200 mM HEPES, pH 7.0) for one hour at room temperature in the dark without shaking. Afterward, UV–Vis spectra were recorded with an Infinite M200 Microplate reader.

### Porphyrin Quantification

The porphyrin extraction was performed as previously described with sight modification.^[^ ^38^, ^59^ ^]^ *E. coli* cultured overnight was diluted 1:100 in BHI medium and incubated at 37 °C at 200 rpm until an OD_600_ _nm_ of 0.4 was achieved. The bacterial suspension containing 1 µM A20 was then prepared and incubated for one hour (37 °C, 200 rpm). The bacterial pellets were harvested (7000 g, 30 min, 4 °C), resuspended in 1 mL EtOAc/acetic acid (3:1, V/V), and lysed by sonication (output setting of 80 W, 15 s on and 15 s off with 4 sonication cycles) with colling breaks in ice. Cell debris was removed by centrifugation (18,000 g, 20 min, 4 °C), and the supernatant was collected. The organic phase was washed twice with water (1 mL), transferred to a new tube, and 3 M HCl (100 µL) was added to water‐solubilize porphyrins. To quantify porphyrins, 100 µL of the aqueous phase were transferred into a black flat bottom 96‐well plate, and the fluorescence spectrum (λ_ex_ = 406 nm, λ_em_ = 550–750 nm) was recorded with a Tecan Infinite 200 microplate reader.

### Respiratory Complex IV Activity

The activity of respiratory complex IV in *E. coli* was determined by the single‐wavelength spectrophotometric assay using the assay kit (Beijing Solarbio Science Technology, Cat No. BC0945).^[^ ^60^ ^]^ Briefly, *E. coli* grown overnight were washed and resuspended to obtain an OD_600_ _nm_ of 1.0. After treatment with A20 for one hour, bacterial cultures were collected and lysed by sonication. After two steps of centrifugation (6000 g for 4 min and then 10,000 g for 10 min) at 4 °C, the activity of complex IV was evaluated through the enzymatic reaction. The absorbance values at a wavelength of 550 nm were detected using the Tecan Infinite 200 microplate reader.

### Horseradish Peroxidase Activity

Horseradish peroxidase (MCE, HY‐125859) (5 ng mL^−1^) in PBS was mixed with an equal volume of A20 (125 µM). After one hour of incubation, a range of hydrogen peroxide (H_2_O_2_, 0–0.21 µM) and 3, 3′, 5, 5′‐Tetramethylbenzidine substrate solution was added. After incubating at 37 °C for 30 min, the optical density was measured at OD_620_ _nm_ using the Tecan Infinite 200 microplate reader. The quantity of reacted H_2_O_2_ was determined by comparing the absorbance values to a pre‐established standard curve. Kinetic parameters were obtained by plotting the H_2_O_2_ concentration against rate and using non‐linear regression to determine *V*_max_ and *K*_M_ by fitting the data to the Michaelis‐Menten equation in GraphPad Prism.

### *Galleria Mellonella* Infection Model

*Galleria mellonella* larvae (purchased from Tianjin Huiyude Biotech Company) were randomly divided into four groups (*n =* 8) and infected with *E. coli* B2 (1.0 × 10^6^ CFUs) at the lower left proleg. After one hour post‐infection, the larvae were treated with 10 µL of A20 (0, 4, 16 mg kg^−1^) and colistin (16 mg kg^−1^) at the right proleg. The survival rates of *G. mellonella* larvae were recorded for six days.

### Mouse Wound Infection Model

A total of 24 female BALB/c mice were randomly divided into four groups and were anesthetized with an intraperitoneal injection of tribromoethanol (200 mg kg^−1^). Subsequently, the dorsal hair was shaved and 10 mm diameter wounds in the backs were made using surgical punches. The wounds were infected with *E. coli* B2 suspension (10^6^ CFUs) and A20 (0, 4, 8 mg kg^−1^), and colistin was topically administered to each wound after 1 h post‐infection. The gross and microscopic appearance of the skin surface was monitored for 12 days and the wound skin was aseptically excised, and homogenized to count bacterial CFU in the skin.

### Statistical Analysis

Results were presented as mean ± standard deviation (SD) of at least three independent biological replicates (*n* ≥ 3). Statistical significance for comparisons between two groups was determined using the unpaired Student's *t*‐test, while multiple group comparisons were performed using one‐way analysis of variance (ANOVA). *P* values were indicated in the figures, with *P* < 0.05 considered significant and non‐significant values (*P* > 0.05) labeled as “n.s.”. Statistical analysis was conducted using GraphPad Prism 9.0 software. Data and materials availability: All data were available in the main text or the supplementary materials.

## Conflict of Interest

The authors declare no conflict of interest.

## Supporting information



Supporting Information

## Data Availability

The data that support the findings of this study are available in the supplementary material of this article.
