# Supplementary material for: Rational Design of Natural Xanthones Against Gram‐negative Bacteria
Source: Adv Sci (Weinh). 2025 Jan 30;12(14):2411923. doi: 10.1002/advs.202411923 (PMC11984908; doi:10.1002/advs.202411923)
Supplement: Supplementary file 1 — Supporting Information [file ADVS-12-2411923-s001.pdf]

ADVANCED SCIENCE
Open Access

Supporting Information

for *Adv. Sci.*, DOI 10.1002/advs.202411923

Rational Design of Natural Xanthones Against Gram-negative Bacteria

Xiaojia Liu, Meirong Song, Ying Liu, Shuyu Yang, Shang Chen, Jijun Kang, Jianzhong Shen and Kui Zhu**

Supplemental Information

Rational Design of Natural Xanthones against Gram-negative Bacteria

Xiaojia Liu†, Meirong Song†, Ying Liu, Shuyu Yang, Shang Chen, Jijun Kang, Jianzhong Shen*, Kui Zhu*

National Key Laboratory of Veterinary Public Health and Safety, College of Veterinary Medicine, China Agricultural University, No.2 Yuanmingyuan West Road, Beijing 100193, China

†These authors contributed equally to this work.

*Corresponding author: Prof. Kui Zhu; Prof. Jianzhong Shen.

Email: zhuk@cau.edu.cn; sjz@cau.edu.cn.

Content list

Methods

Chemical synthesis……...4

Figures

Figure S1……16

Figure S2……17

Figure S3……18

Figure S4……19

Figure S5……20

Figure S6……21

Figure S7……22

Figure S8……23

Figure S9……24

Figure S10……..25

Figure S11……..26

Figure S12……..27

Figure S13……..28

Figure S14……..29

Figure S15……..30

Figure S16……..31

Figure S17……..32

Figure S18……..33

Figure S19……..34

Figure S20……..35

Figure S21……..36

Figure S22……..37

Figure S23……..38

Figure S24……..39

Figure S25……………………………………………………………………..40
Figure S26……………………………………………………………………..41
Figure S27……………………………………………………………………..42
Figure S28……………………………………………………………………..43
Figure S29……………………………………………………………………..44
Figure S30……………………………………………………………………..45
Figure S31……………………………………………………………………..46
Figure S32……………………………………………………………………..47
Figure S33……………………………………………………………………..48
Figure S34……………………………………………………………………..49
Figure S35……………………………………………………………………..50
Figure S36……………………………………………………………………..51
Figure S37……………………………………………………………………..52
Figure S38……………………………………………………………………..53

Tables

Table S1……………………………………………………………………….54
Table S2……………………………………………………………………...…..55
Table S3…………………………………………………………………..……...56
Table S4……………………………………………………………..…………...58
Table S5……………………………………………………………..…………...60
Table S6…………………………………………………………..……………...63
Table S7…………………………………………………………..……………...66
Table S8…………………………………………………………..……………...78
Table S9…………………………………………………………..……….……..81
Table S10…………………………………………………………………….……..82
Table S11…………………………………………………………………….……..83

Methods

Chemical synthesis

Thin-layer chromatography was performed on HSGF 254 plates (Yantai Jiangyou Silicone Development Co., Ltd) and spots were visualized with UV light. Flash column chromatography was performed using 100-200 mesh silica gels (Qingdao Ocean Chemical Industry Co., Ltd). Nuclear magnetic resonance (NMR) spectra were recorded in deuterated dimethyl sulfoxide (DMSO-*d6*), deuterated methanol (CD_3OD), and deuterated chloroform ($CDCl_3$) solution on a Bruker Avance spectrometer operating at 400, 500 or 600 MHz for ^1H NMR and 100 or 150 MHz for ^{13}C NMR. In every case, trimethylsilane (TMS) was used as an internal standard. Chemical shifts are reported in parts per million (ppm). The coupling constant (J) is reported in Hertz (Hz). Analytical reversed-phase high-performed liquid chromatography (HPLC) was performed on a Shimadzu LC-20A equipped with a manual sampler and a photodiode array detector. The chromatographic separation was accomplished on a Shim-pack GIST C18 column (5 µm, 10 × 250 mm).

Synthesis of A1 (Scheme 1)

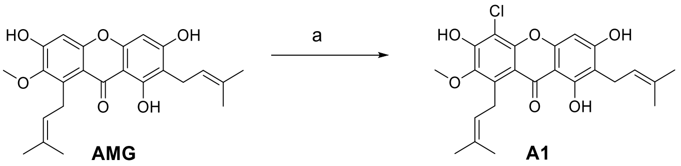

Sheme 1: Reagents and conditions: (a) NCS, DCM, r.t., 30 min.

Following the literature procedures, the chlorinated product **A1** was synthesized by the reaction between the corresponding NCS reagent and AMG which in turn was easily generated.

Compound A1: AMG (100 mg, 0.24 mmol) was dissolved in 2 mL dichloromethane, and NCS (70 mg, 0.53 mmol) was added for stirring. The reaction was carried out at room temperature under the protection of N_2 for 30 min. The reaction solution was diluted with saturated sodium thiosulfate aqueous solution and then extracted with dichloromethane. The organic phase was washed with NaCl solution, dried with Na_2SO_4, and concentrated by rotary evaporator. The crude product was separated and purified by a silica gel column, and eluted by PE-EA (9:1, V/V) to give A1 (24%) as a bright yellow solid. ^1H NMR (400 MHz, DMSO-d_6, δ): 13.72 (s, 1H, -OH), 11.01 (s, 1H, -OH), 10.82 (s, 1H, -OH), 6.80 (s, 1H, Ar-H), 6.34 (s, 1H, Ar-H), 5.16 (m, 2H, 2×-CH=), 4.01 (d, 2H, $-CH_2-$), 3.70 (s, 3H, $-OCH_3$), 3.20 (d, 2H, $-CH_2-$), 1.77 (s, 3H, CH_3), 1.72 (s, 3H, CH_3), 1.62 (s, 6H, 2×CH_3); ^{13}C NMR (150 MHz, DMSO-d_6, δ): 181.3, 158.0, 157.6, 157.4, 154.5, 149.4, 143.8, 136.6, 131.2, 130.6, 123.4, 121.9, 111.2, 109.7, 102.7, 101.9, 97.0, 60.2, 25.8, 25.6, 25.5, 21.6, 18.0, 17.8.

Synthesis of A6-A23 (Scheme 2)

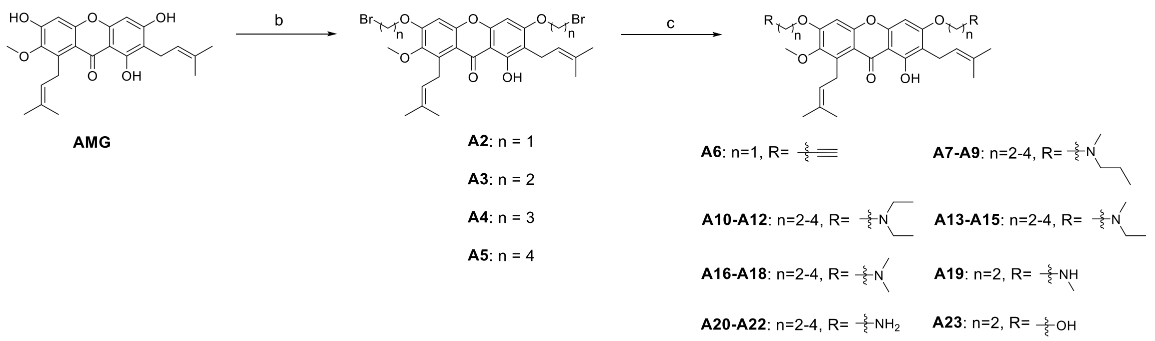

Scheme 2: reagents and conditions: (b) acetone, K_3CO_3, alkanes bromide, reflux 8-12 h; (c) DMSO, corresponding amines, r.t./50 °C, 5-24 h.

Following the literature procedures, the brominated intermediates A2-A5 were obtained by the reaction of substrates with alkanes bromide under alkaline conditions. The intermediates were subjected to nucleophilic reaction with different amines at room temperature to obtain the final products A3-A23.

Compound A3: The title compound was obtained from AMG and 1,2-dibromoethane following the procedure for compound A2 in 68% yield after flash-chromatography (20:1, V/V, PE-EA) as a faint yellow solid. ^{1}H NMR (400 MHz, $CDCl_3$, δ): 13.46 (s, 1H, -OH), 6.69 (s, 1H, Ar-H), 6.26 (s, 1H, Ar-H), 5.27 (m, 1H, CH=), 5.24 (m, 1H, CH=), 4.41 (t, 2H, *J* = 5.9 Hz, $-CH_2-$), 4.37 (t, 2H, *J* = 6.2 Hz, $-CH_2-$), 4.14 (d, 2H, *J* = 6.6 Hz, $-CH_2-$), 3.86 (s, 3H, $-OCH_3$), 3.75 (t, 2H, *J* = 6.0 Hz, $-CH_2-$), 3.69 (t, 2H, *J* = 6.2 Hz, $-CH_2-$), 3.39 (d, 2H, *J* = 7.2 Hz, $-CH_2-$), 1.85 (s, 3H, CH_3), 1.81 (s, 3H, CH_3), 1.68 (s, 6H, 2×CH_3). ^{13}C NMR (100 MHz, $CDCl_3$, δ): 182.1, 161.9, 160.3, 156.6, 155.2, 155.1, 144.3, 138.0, 132.1, 131.8, 123.2, 122.4, 112.8, 112.1, 104.5, 99.1, 89.5, 68.6, 68.3, 61.4, 28.6, 28.5, 26.4, 26.1, 26.0, 21.6, 18.3, 18.0.

Compound A4: The title compound was obtained from AMG and 1,3-dibromopropane following procedure for compound A2 in 70% yield after flash-chromatography (20:1, V/V, PE-EA) as a light yellow amorphous solid. ^{1}H NMR (400 MHz, $CDCl_3$, δ): 13.48 (s, 1H, -OH), 6.77 (s, 1H, Ar-H), 6.33 (s, 1H, Ar-H), 5.24 (m, H, -CH=), 5.21 (m, H, -CH=), 4.24 (t, 2H, *J* = 5.8 Hz, $-CH_2-$), 4.20 (t, 2H, *J* = 5.8 Hz, $-CH_2-$), 4.14 (d, 2H, *J* = 6.5 Hz, $-CH_2-$), 3.80 (s, 3H, $-OCH_3$), 3.66 (t, 2H, *J* = 6.3 Hz, $-CH_2-$), 3.63 (t, 2H, *J* = 6.4 Hz, $-CH_2-$), 3.36 (d, 2H, *J* = 7.0 Hz, $-CH_2-$), 2.44 (m, 2H, $-CH_2-$), 2.38 (m, 2H, $-CH_2-$), 1.85 (s, 3H, CH_3), 1.79 (s, 3H, CH_3), 1.68 (s, 6H, 2×CH_3). ^{13}C NMR (100 MHz, $CDCl_3$, δ): 182.2, 162.5, 160.1, 157.2, 155.4, 155.3, 144.2, 137.7, 132.0, 131.7, 123.3, 122.6, 112.5, 111.8, 104.3, 99.1, 89.5, 66.3, 66.0, 61.1, 32.3, 32.0, 29.8, 29.7, 26.4, 26.0, 25.9, 21.6, 18.3, 18.0.

Compound A5: The title compound was obtained from AMG and 1,4-dibromobutane following procedure for compound A2 in 67% yield after flash-chromatography (20:1, V/V, PE-EA) as a light yellow amorphous solid. ^{1}H NMR

(600 MHz, $CDCl_3$, δ): 13.48 (s, 1H, -OH), 6.70 (s, 1H, Ar-H), 6.28 (s, 1H, Ar-H), 5.24 (t, 1H, *J* = 6.1 Hz, -CH=), 5.21 (t, 1H, *J* = 7.0 Hz, -CH=), 4.13 (m, 2H, $-CH_2-$), 4.12 (m, 2H, $-CH_2-$), 4.08 (t, 2H, *J* = 5.9 Hz, $-CH_2-$), 3.80 (s, 3H, $-OCH_3$), 3.53 (t, 2H, *J* = 6.2 Hz, $-CH_2-$), 3.50 (t, 2H, *J* = 6.5 Hz, $-CH_2-$), 3.35 (d, 2H, *J* = 7.0 Hz, $-CH_2-$), 2.13 (m, 4H, 2×$-CH_2-$), 2.08 (m, 2H, $-CH_2-$), 2.02 (m, 2H, $-CH_2-$), 1.85 (s, 3H, CH_3), 1.79 (s, 3H, CH_3), 1.68 (s, 6H, 2×CH_3); ^{13}C NMR (150 MHz, $CDCl_3$, δ): 182.1, 162.7, 160.0, 157.3, 155.4, 155.2, 144.2, 137.5, 131.9, 131.6, 123.3, 122.6, 112.2, 111.6, 104.1, 98.9, 89.3, 67.9, 67.4, 61.1, 33.4, 33.3, 29.6, 29.5, 27.9, 27.7, 26.3, 26.1, 26.0, 21.6, 18.3, 18.0.

Compound A6: To a solution of intermediate A2 (100 mg, 0.17 mmol) in 5 mL of acetone, cuprous cyanide (67 mg, 0.75 mmol) was added. Then the mixture was stirred at 130 °C for 24 h. Reaction mixture was cooled to room temperature, diluted with water, extracted with EA, washed with NaCl solution, dried over Na_2SO_4, and concentrated. The crude residue was chromatographed on silica gel with PE-EA (3:1, V/V) to give 26 mg (91%) of A6 as a colorless solid. ^1H NMR(400 MHz, DMSO-d_6, δ): 13.47 (s, 1H, -OH), 7.34 (s, 1H, Ar-H), 6.83 (s, 1H, Ar-H), 5.44 (s, 2H, $-CH_2-$), 5.39 (s, 2H, $-CH_2-$), 5.15 (m, 2H, 2×-CH=), 4.05 (t, 2H, *J* = 6.4 Hz, $-CH_2-$), 3.74 (s, 3H, $-OCH_3$), 3.27 (d, 2H, *J* = 7.4 Hz, $-CH_2-$), 1.79 (s, 3H, CH_3), 1.75 (s, 3H, CH_3), 1.63 (s, 6H, 2×CH_3). ^{13}C NMR (150 MHz, $CDCl_3$, δ): 181.8, 160.2, 159.2, 155.2, 154.5, 143.9, 136.9, 131.4, 131.1, 123.0, 121.5, 116.1, 115.9, 112.4, 111.4, 104.1, 100.6, 90.7, 60.9, 54.3, 54.2, 25.6, 25.5, 20.9, 18.0, 17.7.

Compound A7: To a solution of A3 (88mg, 0.24 mmol) in anhydrous THF (3 mL), *N*-methylpropylamine (10 eq.) was added and stirred for 24 h at room temperature. The solvent was removed in vacuo, and crude residue was chromatographed on silica gel with DCM-MeOH (5%-13%) to give of A7 (62%) as a yellow solid. ^1H NMR (400 MHz, CD_3OD, δ): 6.86 (s, 1H, Ar-H), 6.43 (s, 1H, Ar-H), 5.22 (t, 1H, *J* = 6.6 Hz, -CH=), 5.15 (t, 1H, *J* = 6.9 Hz, -CH=), 4.39 (t, 2H, *J* = 5.0 Hz, $-CH_2-$), 4.35 (t, 2H, *J* = 5.2 Hz, $-CH_2-$), 4.05 (d, 2H, *J* = 6.5 Hz, $-CH_2-$), 3.78 (s, 3H, -OCH_3), 3.37 (t, 2H, *J* = 5.0 Hz, $-CH_2-$), 3.31 (m, 4H, 2×$-CH_2-$), 2.92 (m, 2H, $-CH_2-$), 2.88 (m, 2H, $-CH_2-$), 2.75 (s, 3H, CH_3), 2.71 (s, 3H, CH_3), 1.83 (s, 3H, CH_3), 1.78 (s, 3H, CH_3), 1.74 (m, 2H, $-CH_2-$), 1.71 (m, 2H, $-CH_2-$), 1.68 (s, 3H, CH_3), 1.66 (s, 3H, CH_3), 1.01 (m, 3H, CH_3), 0.99 (m, 3H, CH_3); ^{13}C NMR (150 MHz, CD_3OD, δ): 183.3, 163.9, 160.8, 158.7, 156.8, 156.7, 145.6, 138.3, 130.4, 130.0, 124.7, 123.7, 113.2, 112.7, 104.9, 100.5, 90.8, 62.7, 2×61.5, 61.0, 60.9, 56.9, 56.6, 56.5, 54.7, 26.0, 25.9, 25.8, 25.1, 23.3, 22.0, 18.3, 18.1, 11.9, 11.8.

Compound A8: The title compound was obtained from A4 and *N*-methylpropylamine following procedure for compound A7 in 43% yield after flash-chromatography (5%-13%, DCM-MeOH) as a yellow solid. ^1H NMR (400 MHz, CD_3OD, δ): 6.39 (s, 1H, Ar-H), 6.03 (s, 1H, Ar-H), 5.25 (t, 1H, *J* = 5.3 Hz, -CH=),

5.13 (t, 1H, J = 5.6 Hz, -CH=), 3.96 (m, 2H, $-CH_2-$), 3.94 (m, 4H, 2×$-CH_2-$), 3.72 (s, 3H, $-OCH_3$), 3.14 (d, 2H, J = 5.5 Hz, $-CH_2-$), 2.64 (m, 4H, 2×$-CH_2-$), 2.44 (m, 2H, $-CH_2-$), 2.41 (m, 2H, $-CH_2-$), 2.33 (s, 3H, CH_3), 2.31 (s, 3H, CH_3), 2.03 (m, 2H, $-CH_2-$), 1.98 (m, 2H, $-CH_2-$), 1.82 (s, 3H, CH_3), 1.76 (s, 3H, CH_3), 1.70 (s, 3H, CH_3), 1.65 (s, 3H, CH_3), 1.57 (m, 2H, $-CH_2-$), 1.54 (m, 2H, $-CH_2-$), 0.94 (m, 3H, CH_3), 0.92 (m, 3H, CH_3); ^{13}C NMR (150 MHz, CD_3OD, δ): 183.0, 163.9, 160.4, 158.7, 156.4, 156.2, 145.2, 137.6, 131.8, 131.7, 125.1, 124.1, 112.6, 112.2, 104.5, 100.1, 90.5, 68.2, 67.8, 61.3, 60.8, 60.7, 55.3, 55.2, 42.4, 42.3, 27.5, 27.4, 27.1, 26.2, 26.0, 22.4, 20.9, 20.8, 18.5, 18.3, 2×12.2.

Compound A9: The title compound was obtained from A5 with *N*-methylpropylamine as described for A7. Crude reaction was passed through a silica gel column (5%-13%, DCM-MeOH) and provided as a yellow solid (52%). ^{1}H NMR (600 MHz, CD_3OD, δ): 6.92 (s, 1H, Ar-H), 6.47 (s, 1H, Ar-H), 5.22 (t, 1H, J = 5.8 Hz, -CH=), 5.19 (t, 1H, J = 7.3 Hz, -CH=), 4.22 (t, 2H, J = 5.4 Hz, $-CH_2-$), 4.16 (t, 2H, J = 5.9 Hz, $-CH_2-$), 4.10 (d, 2H, J = 6.5 Hz, $-CH_2-$), 3.80 (s, 3H, $-OCH_3$), 3.33 (d, 2H, J = 6.9 Hz, $-CH_2-$), 3.23 (m, 2H, $-CH_2-$), 3.21 (m, 2H, $-CH_2-$), 2.91 (s, 3H, CH_3), 2.90 (s, 3H, CH_3), 2.01 (m, 8H, 4×$-CH_2-$), 1.98 (m, 4H, 2×$-CH_2-$), 1.84 (s, 3H, CH_3), 1.80 (br s, 7H, CH_3 and 2×$-CH_2-$), 1.68 (s, 3H, CH_3), 1.67 (s, 3H, CH_3), 1.32 (t, 6H, J = 7.3 Hz, 2×CH_3); ^{13}C NMR (150 MHz, CD_3OD, δ): 183.2, 164.0, 160.7, 158.8, 156.8, 156.6, 145.4, 138.1, 132.1, 132.0, 124.8, 123.7, 112.9, 112.4, 104.7, 100.4, 90.8, 69.4, 68.9, 61.5, 58.9, 58.8, 57.1, 57.0, 2×47.9, 2×40.5, 27.4, 27.1, 27.0, 26.1, 26.0, 22.4, 22.3, 22.2, 18.7, 18.6, 18.4, 18.1.

Compound A10: To a mixture of compound A3 (88mg, 0.24 mmol) and diethylamine (10 eq.) in 3 mL anhydrous THF was stirred at room temperature for 18 h. After completion of the reaction, the solvent was removed and the residue was purified by silica gel column chromatography (5%-13%, methanol in dichloromethane) and afforded compound A10 (43 mg, 58%) as a yellow solid. ^{1}H NMR (400 MHz, CD_3OD, δ): 6.85 (s, 1H, Ar-H), 6.43 (s, 1H, Ar-H), 5.21 (t, 1H, J = 6.6 Hz, -CH=), 5.12 (t, 1H, J = 6.7 Hz, -CH=), 4.39 (m, 4H, 2×$-CH_2-$), 4.03 (d, 2H, J = 6.6 Hz, $-CH_2-$), 3.76 (s, 3H, $-OCH_3$), 3.50 (t, 2H, J = 4.9 Hz, $-CH_2-$), 3.45 (t, 2H, J = 4.9 Hz, $-CH_2-$), 3.45 (m, 2H, $-CH_2-$), 3.26 (d, 2H, J = 6.5 Hz, $-CH_2-$), 3.19 (m, 4H, 2×$-CH_2-$), 3.16 (m, 4H, 2×$-CH_2-$), 1.83 (s, 3H, CH_3), 1.77 (s, 3H, CH_3), 1.68 (s, 3H, CH_3), 1.66 (s, 3H, CH_3), 1.32 (m, 6H, 2×CH_3), 1.30 (m, 6H, 2×CH_3); ^{13}C NMR (150 MHz, CD_3OD, δ): 180.0, 162.4, 159.4, 157.2, 155.4, 155.2, 144.1, 136.9, 130.7, 129.0, 128.6, 123.3, 122.4, 111.8, 111.3, 103.5, 99.2, 61.2, 60.1, 55.2, 53.3, 51.0, 50.8, 50.4, 45.6, 40.4, 23.7, 21.9, 20.9, 20.6, 16.9, 16.7, 2×9.7, 2×9.6.

Compound A11: The title compound was obtained from A4 and diethylamine as described for A16 in 61% yield after flash-chromatography (5%-13%, methanol in dichloromethane) as an amorphous yellow powder. ^{1}H NMR (400 MHz, CD_3OD, δ):

6.61 (s, 1H, Ar-H), 6.22 (s, 1H, Ar-H), 5.25 (t, 1H, J = 5.3 Hz, -CH=), 5.16 (t, 1H, J = 5.5 Hz, -CH=), 4.05 (m, 6H, 3×-CH_2-), 3.76 (s, 3H, -OCH_3), 3.22 (d, 2H, J = 5.3 Hz, -CH_2-), 2.85 (m, 2H, -CH_2-), 2.82 (m, 2H, -CH_2-), 2.75 (m, 4H, 2×-CH_2-), 2.72 (m, 4H, 2×-CH_2-), 2.07 (m, 2H, -CH_2-), 2.03 (m, 2H, -CH_2-), 1.84 (s, 3H, CH_3), 1.77 (s, 3H, CH_3), 1.70 (s, 3H, CH_3), 1.66 (s, 3H, CH_3), 1.15 (m, 6H, 2×CH_3), 1.13 (m, 6H, 2×CH_3); ^{13}C NMR (150 MHz, CD_3OD, δ): 183.2, 164.0, 160.5, 158.8, 156.6, 156.5, 145.4, 137.9, 132.0, 131.9, 130.9, 124.9, 123.9, 112.8, 112.4, 104.6, 100.2, 90.6, 68.2, 67.7, 61.4, 53.6, 50.4, 50.3, 48.1, 48.0, 27.0, 26.8, 26.5, 26.1, 26.0, 22.3, 18.4, 18.2, 2×11.1, 2×11.0.

Compound A12: The title compound was obtained from A5 and diethylamine as described for A16 in 53% yield after flash-chromatography (5%-13%, methanol in dichloromethane) as an amorphous yellow powder. ^{1}H NMR (600 MHz, CD_3OD, δ): 6.35 (s, 1H, Ar-H), 5.96 (s, 1H, Ar-H), 5.25 (t, 1H, J = 6.8 Hz, -CH=), 5.14 (t, 1H, J = 7.3 Hz, -CH=), 3.95 (d, 2H, J = 6.4 Hz, -CH_2-), 3.89 (m, 4H, 2×-CH_2-), 3.71 (s, 3H, -OCH_3), 3.14 (d, 2H, J = 7.0 Hz, -CH_2-), 2.59 (m, 4H, 2×-CH_2-), 2.57 (m, 4H, 2×-CH_2-), 2.54 (m, 4H, 2×-CH_2-), 1.82 (s, 3H, CH_3), 1.78 (m, 4H, 2×-CH_2-), 1.75 (s, 3H, CH_3), 1.69 (s, 3H, CH_3), 1.67 (m, 4H, 2×-CH_2-), 1.65 (s, 3H, CH_3), 1.07 (m, 6H, 2×CH_3), 1.05 (m, 6H, 2×CH_3); ^{13}C NMR (150 MHz, CD_3OD, δ): 182.8, 163.9, 160.3, 158.6, 156.1, 156.0, 145.1, 137.3, 131.6, 131.4, 125.3, 124.1, 112.4, 112.0, 104.3, 99.9, 90.4, 69.7, 69.3, 61.2, 2×53.5, 4×47.7, 28.6, 28.4, 27.1, 26.3, 26.2, 23.8, 23.7, 22.4, 18.6, 18.3, 2×11.5, 2×11.4.

Compound A13: To the solution of A3 (88mg, 0.24 mmol) in anhydrous DMSO (3 mL) was added *N*-methylethylamine (10 eq.). The reaction mixture was stirred for approximately 24 h at room temperature. The solution was then diluted with two times volume of water, and extracted with *n*-butanol. The organic layer wad concentrated in vacuo. The crude residue was purified by silica gel column chromatography (5%-13%, DCM-MeOH) and provided the desired product (35 mg, 67%) as an amorphous powder. ^{1}H NMR (400 MHz, CD_3OD, δ): 6.56 (s, 1H, Ar-H), 6.17 (s, 1H, Ar-H), 5.23 (t, 1H, J = 6.4 Hz, -CH=), 5.13 (t, 1H, J = 6.9 Hz, -CH=), 4.14 (m, 4H, 2×-CH_2-), 3.96 (d, 2H, J = 6.4 Hz, -CH_2-), 3.72 (s, 3H, -OCH_3), 3.18 (d, 2H, J = 6.9 Hz, -CH_2-), 3.01 (t, 2H, J = 5.5 Hz, -CH_2-), 2.97 (d, 2H, J = 5.5 Hz, -CH_2-), 2.70 (m, 4H, 2×CH_2), 2.48 (s, 3H, CH_3), 2.45 (s, 3H, CH_3), 1.81 (s, 3H, CH_3), 1.75 (s, 3H, CH_3), 1.69 (s, 3H, CH_3), 1.65 (s, 3H, CH_3), 1.17 (m, 6H, 2×CH_3). ^{13}C NMR (150 MHz, CD_3OD, δ): 182.9, 163.5, 160.5, 158.3, 156.2, 156.1, 145.2, 137.8, 2×131.8, 125.0, 124.0, 112.8, 112.3, 104.6, 100.2, 90.6, 67.6, 67.4, 61.4, 56.4, 56.2, 2×52.9, 42.4, 42.3, 27.1, 26.2, 26.0, 22.4, 18.5, 18.3, 2×12.0.

Compound A14: The title compound was obtained from A4 and *N*-methylethylamine as described for A13 in 68% yield after flash-chromatography (5%-13%, methanol in dichloromethane) as an amorphous yellow powder. ^{1}H NMR (400

MHz, CD_3OD, δ): 6.41 (s, 1H, Ar-H), 6.03 (s, 1H, Ar-H), 5.24 (t, 1H, J = 5.6 Hz, -CH=), 5.12 (t, 1H, J = 6.0 Hz, -CH=), 3.94 (m, 6H, 3×-CH_2-), 3.71 (s, 3H, -OCH_3), 3.13 (d, 2H, J = 6.6 Hz, -CH_2-), 2.70 (m, 4H, 2×-CH_2-), 2.60 (m, 4H, 2×-CH_2-), 2.37 (s, 3H, CH_3), 2.34 (s, 3H, CH_3), 2.03 (m, 4H, 2×CH_2), 1.81 (s, 3H, CH_3), 1.75 (s, 3H, CH_3), 1.69 (s, 3H, CH_3), 1.65 (s, 3H, CH_3), 1.14 (m, 6H, 2×CH_3). ^{13}C NMR (150 MHz, CD_3OD, δ): 182.8, 163.7, 160.2, 158.5, 156.1, 156.0, 145.1, 137.4, 131.7, 131.6, 125.1, 124.1, 112.5, 112.1, 104.4, 100.0, 90.4, 68.0, 67.7, 61.3, 54.8, 54.7, 52.4, 52.3, 41.6, 41.5, 27.4, 27.2, 27.1, 26.2, 26.0, 22.4, 18.5, 18.3, 2×11.8.

Compound A15: The title compound was obtained from A5 and *N*-methylethylamine as described for A13 in 75% yield after flash-chromatography (5%-13%, methanol in dichloromethane) as an amorphous yellow powder. ^{1}H NMR (400 MHz, CD_3OD, δ): 6.90 (s, 1H, Ar-H), 6.45 (s, 1H, Ar-H), 5.19 (m, 2H, 2×-CH=), 4.21 (m, 2H, -CH_2-), 4.15 (m, 2H, -CH_2-), 4.01 (d, 2H, J = 6.5 Hz, -CH_2-), 3.80 (s, 3H, -OCH_3), 3.26 (m, 6H, 2×-CH_2-), 3.20 (m, 4H, 2×-CH_2-), 2.87 (s, 3H, CH_3), 2.86 (s, 3H, CH_3), 2.01 (m, 4H, 2×-CH_2-), 1.97 (m, 4H, 2×-CH_2-), 1.36 (m, 6H, 2×CH_3). ^{13}C NMR (100 MHz, CD_3OD, δ): 183.2, 164.0, 160.6, 158.8, 156.8, 156.6, 145.4, 138.0, 2×132.1, 124.8, 123.7, 112.9, 112.5, 104.7, 100.4, 90.8, 69.5, 68.9, 61.5, 2×56.5, 52.5, 52.4, 39.9, 39.8, 27.4, 27.1, 27.0, 26.1, 26.0, 22.5, 2×22.4, 18.4, 18.4, 2×9.6.

Compound A16: To the solution of A3 (88mg, 0.24 mmol) in anhydrous THF (3 mL) was added dimethylamine (10 eq.). The reaction mixture was stirred for approximately 24 h at room temperature. The solution was then concentrated in vacuo. Crude residue was purified by silica gel column chromatography (5%-13%, DCM-MeOH) and provided the desired product (35 mg, 58%) as an amorphous powder. ^{1}H NMR (400 MHz, CD_3OD, δ): 6.68 (s, 1H, Ar-H), 6.27 (s, 1H, Ar-H), 5.23 (t, 1H, J = 6.6 Hz, -CH=), 5.14 (t, 1H, J = 6.9 Hz, -CH=), 4.17 (m, 4H, 2×-CH_2-), 4.00 (d, 2H, J = 6.6 Hz, -CH_2-), 3.75 (s, 3H, -OCH_3), 3.22 (d, 2H, J = 6.9 Hz, -CH_2-), 2.96 (d, 2H, J = 5.5 Hz, -CH_2-), 2.91 (d, 2H, J = 5.5 Hz, -CH_2-), 2.47 (s, 6H, 2×CH_3), 2.45 (s, 6H, 2×CH_3), 1.82 (s, 3H, CH_3), 1.77 (s, 3H, CH_3), 1.69 (s, 3H, CH_3), 1.65 (s, 3H, CH_3).

Compound A17: The title compound was obtained from A4 as described for A16 in 65% yield after flash-chromatography (5%-13%, DCM-MeOH) as an amorphous yellow solid. ^{1}H NMR (400 MHz, CD_3OD, δ): 6.55 (s, 1H, Ar-H), 6.17 (s, 1H, Ar-H), 5.25 (t, 1H, J = 5.3 Hz, -CH=), 5.14 (t, 1H, J = 5.6 Hz, -CH=), 4.03 (m, 4H, 2×-CH_2-), 4.00 (d, 2H, J = 5.6 Hz, -CH_2-), 3.75 (s, 3H, -OCH_3), 3.19 (d, 2H, J = 5.3 Hz, -CH_2-), 2.74 (m, 2H, -CH_2-), 2.70 (m, 2H, -CH_2-), 2.45 (s, 6H, 2×CH_3), 2.42 (s, 6H, 2×CH_3), 2.11 (m, 2H, -CH_2-), 2.06 (m, 2H, -CH_2-), 1.84 (s, 3H, CH_3), 1.77 (s, 3H, CH_3), 1.70 (s, 3H, CH_3), 1.66 (s, 3H, CH_3); ^{13}C NMR (150 MHz, CD_3OD, δ): 183.1, 164.0, 160.5, 158.7, 156.5, 156.4, 145.3, 137.8, 131.9, 131.8, 124.9, 124.0, 112.8, 112.4,

104.6, 100.2, 90.6, 67.9, 67.5, 61.4, 57.3, 57.2, 2×45.2, 2×45.1, 27.6, 27.5, 27.0, 26.1, 26.0, 22.3, 18.4, 18.2.

Compound A18: The title compound was obtained from A5 as described for A16 in 69% yield after flash-chromatography (5%-13%, DCM-MeOH) as an amorphous yellow solid. ^{1}H NMR (600 MHz, CD_3OD, δ): 6.55 (s, 1H, Ar-H), 6.15 (s, 1H, Ar-H), 5.25 (t, 1H, J = 6.7 Hz, -CH=), 5.16 (t, 1H, J = 7.3 Hz, -CH=), 4.01 (m, 4H, 2×-CH_2-), 3.98 (t, 2H, J = 6.1 Hz, -CH_2-), 3.75 (s, 3H, -OCH_3), 3.20 (d, 2H, J = 7.1 Hz, -CH_2-), 2.47 (m, 2H, -CH_2-), 2.45 (m, 2H, -CH_2-), 2.30 (s, 6H, 2×CH_3), 2.29 (s, 6H, 2×CH_3), 1.87 (m, 4H, 2×-CH_2-), 1.83 (s, 3H, CH_3), 1.77 (s, 3H, CH_3), 1.73 (m, 4H, 2×-CH_2-), 1.69 (s, 3H, CH_3), 1.65 (s, 3H, CH_3); ^{13}C NMR (150 MHz, CD_3OD, δ): 183.0, 164.0, 160.4, 158.7, 156.4, 156.2, 145.2, 137.5, 131.7, 131.6, 125.1, 124.0, 112.6, 112.2, 104.4, 100.0, 90.5, 69.7, 69.3, 61.3, 60.4, 60.3, 4×45.3, 28.2, 28.1, 27.0, 26.2, 26.0, 2×24.9, 22.4, 18.5, 18.2.

Compound A19: To a solution of A3 (187 mg，0.3 mmol) in methylamine solution (2.0 M in THF, 5 mL) was stirred at room temperature for 96 h. After completion of the reaction, the solvent was removed and the residue was purified by silica gel column chromatography (5%-13%, methanol in dichloromethane) and afforded compound A19 (113 mg, 72%) as a yellow light powder. ^{1}H NMR (400 MHz, DMSO-d_6, δ): 7.05 (s, 1H, Ar-H), 6.55 (s, 1H, Ar-H), 5.15 (m, 2H, 2×-CH=), 4.20 (t, 2H, J = 4.1 Hz, -CH_2-), 4.14 (t, 2H, J = 3.9 Hz, -CH_2-), 4.02 (d, 2H, J = 5.0 Hz, -CH_2-), 3.73 (s, 3H, -OCH_3), 3.25 (d, 2H, J = 5.6 Hz, -CH_2-), 2.92 (t, 2H, J = 3.9 Hz, -CH_2-), 2.88 (t, 2H, J = 4.6 Hz, -CH_2-), 2.37 (s, 3H, CH_3), 2.36 (s, 3H, CH_3), 1.77 (s, 3H, CH_3), 1.73 (s, 3H, CH_3), 1.62 (s, 6H, 2×CH_3).

Compound A20: Compound A3 (0.3 mmol) was dissolved in 5 mL of ammonia solution (7.0 M in MeOH) at room temperature. The reaction mixture was stirred at room temperature for 96 h and then concentrated in vacuo. The crude reaction mixture was passed through a silica gel column with DCM-MeOH (50:1-30:1, containing 0.1% TEA) to give 113 mg (72%) of A20 as a yellow powder. ^{1}H NMR (400 MHz, DMSO-d_6, δ): 7.02 (s, 1H, Ar-H), 6.51 (s, 1H, Ar-H), 5.15 (m, 2H, 2×-CH=), 4.10 (t, 2H, J = 5.6 Hz, -CH_2-), 4.04 (t, 2H, J = 5.5 Hz, -CH_2-), 4.01 (d, 2H, J = 6.5 Hz, -CH_2-), 3.73 (s, 3H, -OCH_3), 3.25 (d, 2H, J = 7.1 Hz, -CH_2-), 2.96 (t, 2H, J = 5.6 Hz, -CH_2-), 2.92 (t, 2H, J = 5.5 Hz, -CH_2-), 1.77 (s, 3H, CH_3), 1.73 (s, 3H, CH_3), 1.62 (s, 6H, 2×CH_3). ^{13}C NMR (150 MHz, DMSO-d_6, δ): 181.5, 162.7, 158.8, 157.6, 154.9, 154.7, 143.9, 135.7, 130.7, 130.6, 123.4, 122.3, 110.8, 110.6, 102.9, 99.5, 89.9, 79.2, 71.4, 71.1, 60.4, 40.8, 40.6, 25.6, 25.5, 21.0, 18.0, 17.7.

Compound A21: The title compound was obtained from A4 as described for A20. Crude reaction mixture was passed through a silica gel column with DCM-MeOH (50:1-30:1, containing 0.1% TEA) to give 113 mg (65%) of A21 as a yellow

powder. ^{1}H NMR (400 MHz, DMSO-d_6, δ): 13.50 (s, 1H, -OH), 6.73 (s, 1H, Ar-H), 6.30 (s, 1H, Ar-H), 5.25 (m, 2H, 2×-CH=), 4.13 (d, 2H, -CH_2-), 4.09 (t, 2H, -CH_2-), 4.07 (t, 2H, -CH_2-), 3.79 (s, 3H, -OCH_3), 3.36 (d, 2H, -CH_2-), 2.58 (m, 8H, 4× -CH_2-), 2.45 (m, 2H, -CH_2-), 2.37 (m, 2H, -CH_2-), 1.78 (s, 3H, CH_3), 1.74 (s, 3H, CH_3), 1.62 (s, 6H, 2×CH_3).

Compound A22: The title compound was obtained from A5 following procedure for compound A7 in 65% yield after flash-chromatography (50:1-30:1, containing 0.1% TEA, DCM-MeOH) as a yellow light powder. ^{1}H NMR(400 MHz, CD_3OD, δ): 6.98 (s, 1H, Ar-H), 6.51 (s, 1H, Ar-H), 5.21 (m, 2H, 2×-CH=), 4.22 (d, 2H, J = 5.5 Hz, -CH_2-), 4.16 (t, 2H, J = 5.4 Hz, -CH_2-), 4.13 (d, 2H, J = 6.7 Hz, -CH_2-), 3.80 (s, 3H, -OCH_3), 3.36 (d, 2H, J = 6.2 Hz, -CH_2-), 3.06 (m, 4H, 2×-CH_2-), 2.01 (m, 4H, 2×-CH_2-), 1.93 (m, 4H, 2×-CH_2-), 1.84 (s, 3H, CH_3), 1.80 (s, 3H, CH_3), 1.67 (s, 3H, CH_3), 1.66 (s, 3H, CH_3).

Compound A23: AMG (100 mg, 0.24 mmol) was dissolved in DMF (5 mL) followed by the addition of K2CO3 (168 mg, 1.22 mmol) and 2-Bromoethanol (304 mg, 2.44 mmol). The reaction mixture was refluxed for about 5 h at 90 °C. The reaction mixture was diluted with water, extracted with ethyl acetate, dried over Na_2SO_4, and concentrated. The crude residue was chromatographed on silica gel with DCM-MeOH (5%-13%) to give 79 mg (65%) of A23 as a yellow solid. ^{1}H NMR (400 MHz, DMSO-d_6, δ): 13.53 (s, 1H, -OH), 7.07 (s, 1H, Ar-H), 6.56 (s, 1H, Ar-H), 5.19 (t, 1H, J = 7.4 Hz, -CH=), 5.16 (t, 1H, J = 6.7 Hz, -CH=), 4.98 (t, 1H, J = 4.2 Hz, -OH), 4.92 (t, 1H, J = 4.3 Hz, -OH), 4.20 (t, 2H, J = 3.6 Hz, -CH_2-), 4.13 (t, 1H, J = 3.8 Hz, -CH_2-), 4.03 (d, 2H, J = 6.7 Hz, -CH_2-), 3.81 (m, 2H, -CH_2-), 3.78 (m, 2H, -CH_2-), 3.75 (s, 3H, -OCH_3), 3.27 (d, 2H, J = 7.4 Hz, -CH_2-), 1.78 (s, 3H, CH_3), 1.74 (s, 3H, CH_3), 1.62 (s, 6H, 2×CH_3); ^{13}C NMR (150 MHz, DMSO-d_6, δ): 181.5, 162.7, 158.8, 157.7, 154.8, 154.6, 143.9, 135.6, 130.6, 130.5, 123.4, 122.1, 110.7, 102.8, 99.6, 90.0, 70.8, 70.5, 60.3, 59.5, 59.3, 25.5, 25.5, 21.0, 18.0, 17.6.

Synthesis of A24-A26 (Scheme 3)

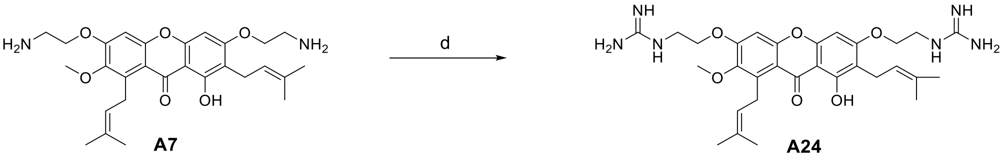

Scheme 3: reagents and conditions: (d) anhydrous DMF, 1*H*-pyrazole-1-carboxamidine hydrochloride, DIPEA, r.t., overnight.

Compound A24: To a solution of A7 (37 mg, 0.08 mmol) in DMF (1 mL) was added 1*H*-pyrazole-1-carboxamidine hydrochloride (3 eq.) and diisopropylethylamine trihydrofluoride (3 eq.). The reaction mixture was stirred overnight at room temperature, after completing, ether was added and then filtered and the insoluble

solid was washed with ether twice. The crude residue was recrystallized by methanol-methyl tert-butyl ether and provided the desired product A24 (27 mg, 62%). ^{1}H NMR (400 MHz, DMSO-d_6) δ: ^{1}H NMR (400 MHz, DMSO-d_6, δ): 13.54 (s, 1H, -OH), 7.78 (m, 2H, 2×-NH), 7.11 (s, 1H, Ar-H), 6.61 (s, 1H, Ar-H), 5.18 (m, 2H, 2×-CH=), 4.27 (t, 2H, J = 4.8 Hz, -CH$_2$-), 4.23 (t, 2H, J = 5.0 Hz, -CH$_2$-), 4.04 (d, 2H, J = 5.7 Hz, -CH$_2$-), 3.74 (s, 3H, -OCH$_3$), 3.66 (m, 2H, -CH$_2$-), 3.61 (m, 2H, -CH$_2$-), 3.29 (d, 2H, J = 7.2 Hz, -CH$_2$-), 1.78 (s, 3H, CH$_3$), 1.74 (s, 3H, CH$_3$), 1.63 (s, 6H, 2×CH$_3$); ^{13}C NMR (150 MHz, DMSO-d_6, δ): 181.6, 162.2, 159.0, 157.3, 157.2, 157.1, 154.8, 154.7, 143.9, 136.1, 130.9, 130.8, 123.3, 122.1, 111.2, 110.9, 103.2, 99.8, 90.2, 67.3, 66.8, 60.7, 40.4, 40.2, 25.7, 2×25.6, 21.1, 18.1, 17.7.

Synthesis of A25 (Scheme 4)

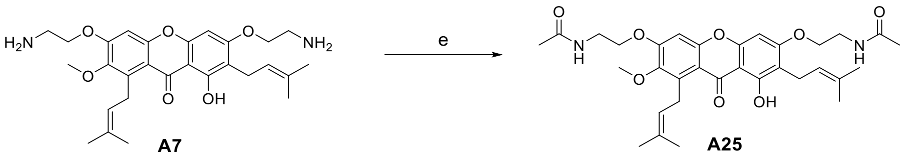

Scheme 4: reagents and conditions: (e) AcOH, Ac$_2$O, MSA, 70 °C, 30 min.

Compound A25: To a solution of A7 (37 mg，0.08 mmol) in acetic acid solution (1 mL) was added acetic anhydride (25 mg, 0.24 mmol) and methane-sulfonic acid (23 mg, 0.24 mmol). The reaction mixture was stirred for approximately 30 min at 70 °C. The solution was then cooled to room temperature and diluted with water, and extracted with EA. The organic layer was washed with NaHCO$_3$ and NaCl solution and dried over Na$_2$SO$_4$, and concentrated in vacuo. The crude residue was purified by silica gel column chromatography (10:1, PE-EA) and provided the desired product A25 (23 mg, 52%). ^{1}H NMR (400 MHz, CDCl$_3$, δ): 13.49 (s, 1H, -OH), 6.74 (s, 1H, Ar-H), 6.30 (s, 1H, Ar-H), 6.05 (t, 1H, J = 6.4 Hz, -NH), 5.89 (t, 1H, J = 5.7 Hz, -NH), 5.22 (m, 2H, 2×-CH=), 4.17 (t, 2H, J = 5.2 Hz, -CH$_2$-), 4.13 (m, 4H, 2×-CH$_2$-), 3.80 (s, 3H, -OCH$_3$), 3.76 (m, 2H, -CH$_2$-), 3.73 (m, 2H, -CH$_2$-), 3.37 (d, 2H, J = 6.8 Hz, -CH$_2$-), 2.03 (s, 3H, CH$_3$), 2.01 (s, 3H, CH$_3$), 1.85 (s, 3H, CH$_3$), 1.81 (s, 3H, CH$_3$), 1.70 (s, 3H, CH$_3$), 1.69 (s, 3H, CH$_3$); ^{13}C NMR (150 MHz, CDCl$_3$, δ): 182.1, 170.6, 170.4, 162.3, 160.0, 157.0, 155.3, 155.2, 144.0, 137.6, 132.1, 131.7, 123.2, 122.8, 112.5, 111.5, 104.2, 99.4, 89.6, 67.9, 67.5, 61.2, 39.1, 39.0, 26.3, 26.1, 26.0, 23.4, 23.3, 21.6, 18.3, 18.0.

Synthesis of A30-A33 (Scheme 5)

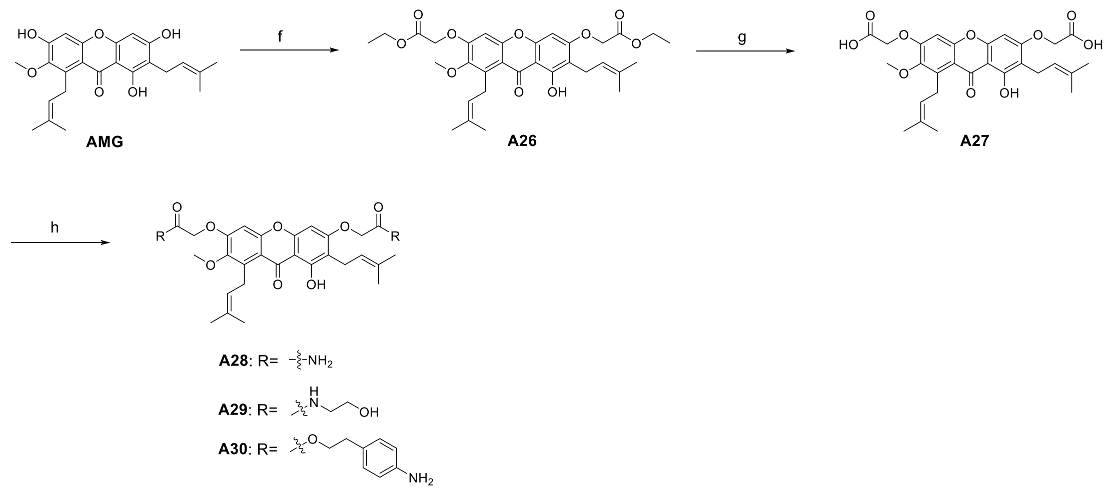

Scheme 5: reagents and conditions: (f) ACN, K_3CO_3, ethyl chloroacetate, reflux 12 h; (g) anhydrous THF, LiOH (5%), r.t., 2 h; (h) anhydrous DMF, NH_4Cl or MEA or Fomc-4-(2-chloroethyl) aniline, EDCl, DMAP, r.t., 12 h.

Compound A28: To a solution of AMG (1.0 g, 7.29 mmol) in acetonitrile (20 mL), K_2CO_3 (1.0 g, 7.29 mmol) and ethyl chloroacetate (0.89 g, 7.29 mmol) were added. The mixture was stirred at reflux for 12 h. After completion of the reaction, mixture was cooled to room temperature, water was added, and extracted with EtOAc. The extract was washed with saturated solution of NaCl, then dried over Na_2SO_4 and concentrated in vacuo. The crude residue was purified by a silica gel column chromatography with PE and EA (8:1) to give intermediate A26 (1.30 g, 92%). The intermediate A26 (1.0 g， 1.71 mmol) was dissolved in THF (5 mL) at room temperature followed by the addition of 5% lithium hydroxide solution (8 mL). The reaction mixture was stirred at room temperature for 2 h. After the completion of the reaction, reaction mixture was neutralized with acetic acid. The mixture was extracted with EA, washed with saturated solution of NaCl, then dried over Na_2SO_4 and concentrated in vacuo. The crude residue was purified by a silica gel column chromatography with PE and EA (1:1) to give intermediate A27 (753 mg, 84%). To a solution of A27 (100 mg) in DMF (3 mL), ammonium chloride (30 mg, 0.57 mmol), 1-ethyl-(3-dimethylaminopropyl) carbodiimide hydrochloride (218 mg, 1.14 mmol) and dimethyl-amino-pyridine (100 mg, 0.19 mmol) were added and refluxed for 12 h. The reaction mixture was extracted with EA. The organic layer was washed with saturated solution of NaCl, then dried over Na_2SO_4 and concentrated in vacuo. The crude residue was purified by a silica gel column chromatography with PE and EA (20:1) to give intermediate A28 (92 mg, 92%).^{1}H NMR (400 MHz, DMSO-d_6, δ): 13.51 (s, 1H, -OH), 7.54 (s, 1H, -NH), 7.49 (br s, 2H, 2×-NH), 7.40 (s, 1H, -NH), 6.97 (s, 1H, Ar-H), 6.52 (s, 1H, Ar-H), 5.18 (m, 2H, 2×-CH=), 4.73 (s, 2H, -CH_2-), 4.63 (s, 2H, -CH_2-), 4.04 (t, 2H, J = 6.3 Hz, -CH_2-), 3.77 (s, 3H, -OCH_3), 3.32 (m, 2H, -CH_2-), 1.79 (s, 3H, CH_3), 1.74 (s, 3H, CH_3), 1.62 (s, 6H, 2×CH_3); ^{13}C NMR (150 MHz, DMSO-d_6, δ): 181.6, 169.0, 168.7, 161.8, 159.0, 156.9, 154.6, 154.5, 143.9, 136.1,

130.9, 130.7, 123.3, 122.3, 111.3, 111.0, 103.2, 100.0, 90.3, 67.3, 67.2, 60.5, 25.6, 25.5, 21.1, 18.0, 17.7.

Compound A29: The title compound was obtained from A27 and ethanolamine (35 mg, 0.57 mmol) as described for A28 in 65% yield after flash-chromatography (20:1, DCM-MeOH) as an amorphous white solid. ^{1}H NMR (400 MHz, DMSO-d_6, δ): 13.52 (s, 1H, -OH), 8.07 (t, 1H, *J* = 5.8 Hz, -NH), 7.93 (t, 1H, *J* = 5.8 Hz, -NH), 6.99 (s, 1H, Ar-H), 6.52 (s, 1H, Ar-H), 5.18 (m, 2H, 2×-CH=), 4.77 (m, 4H, 2×-CH_2-), 4.68 (s, 2H, -OH), 4.04 (t, 2H, *J* = 6.4 Hz, -CH_2-), 3.77 (s, 3H, -OCH_3), 3.45 (m, 4H, 2×-CH_2-), 3.32 (m, 2H, -CH_2-), 3.22 (m, 4H, 2×-CH_2-), 1.79 (s, 3H, CH_3), 1.73 (s, 3H, CH_3), 1.62 (s, 6H, 2×CH_3); ^{13}C NMR (150 MHz, DMSO-d_6, δ): 181.6, 166.9, 166.7, 161.8, 159.0, 156.9, 154.6, 154.5, 143.9, 136.1, 131.0, 130.8, 123.3, 122.1, 111.3, 111.1, 103.3, 100.1, 90.4, 67.6, 67.4, 60.5, 2×59.6, 2×41.3, 2×25.6, 25.5, 21.1, 18.0, 17.7.

Compound A30: The title compound of the intermediate was obtained from A27 (100 mg, 0.19 mmol) and *p*-Fmoc-4-(2-chloroethyl) aniline (147 mg, 0.57 mmol) as described for A28 in 88% yield after flash-chromatography (5:1, PE-EA) as an amorphous solid. The intermediate was dissolved in THF (5 mL) followed by addition of piperidine (15 mg， 0.32 mmol). The reaction mixture was stirred for 2 h at room temperature. The mixture was extracted with EtOAc, washed with saturated solution of NaCl, then dried over Na_2SO_4 and concentrated in vacuo. Crude residue was purified by silica gel column chromatography (2:1, PE-EA) and provided the desired product A30 (52 mg, 80%). ^{1}H NMR (400 MHz, DMSO-d_6, δ): 13.53 (s, 1H, -OH), 7.05 (s, 1H, Ar-H), 6.87 (m, 4H, 4×Ar-H), 6.53 (s, 1H, Ar-H), 6.47 (m, 4H, 4×Ar-H), 5.22 (t, 1H, *J* = 7.5 Hz, -CH=), 5.17 (t, 1H, *J* = 6.5 Hz, -CH=), 5.07 (s, 2H, -CH_2-), 4.98 (s, 2H, -CH_2-), 4.89 (s, 4H, 4×-NH), 4.25 (m, 4H, 2×-CH_2-), 4.05 (d, 2H, *J* = 6.2 Hz, -CH_2-), 3.75 (s, 3H, -OCH_3), 3.30 (d, 2H, *J* = 6.7 Hz, -CH_2-), 2.73 (m, 4H, 2×-CH_2-), 1.79 (s, 3H, CH_3), 1.74 (s, 3H, CH_3), 1.63 (s, 3H, CH_3), 1.62 (s, 3H, CH_3). ^{13}C NMR (150 MHz, DMSO-d_6, δ): 181.6, 168.1, 167.9, 161.6, 159.1, 156.5, 154.6, 154.5, 147.1, 143.8, 136.2, 130.9, 130.8, 2×129.3, 124.3, 124.2, 123.3, 122.0, 2×114.1, 111.4, 111.1, 103.4, 99.9, 90.3, 65.9, 65.9, 65.3, 65.2, 60.4, 33.6, 25.6, 25.5, 21.1, 18.0, 17.7.

Synthesis of A32 (Scheme 6)

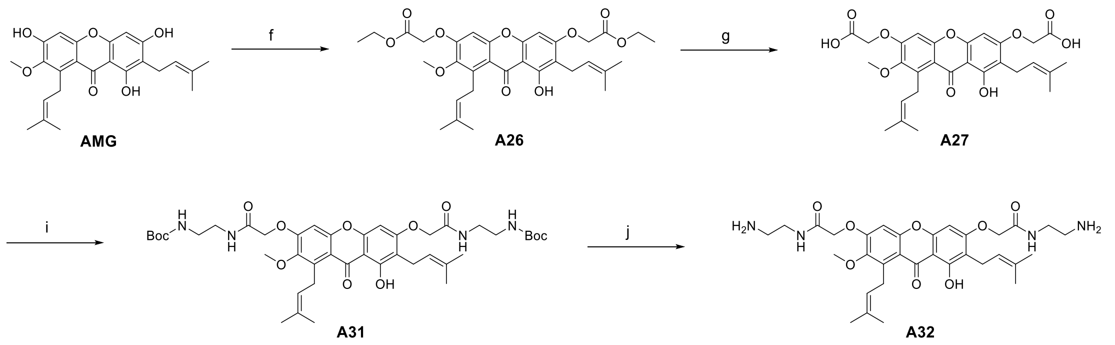

Scheme 6: reagents and conditions: (i) EDCl, DMAP, *N*-Boc-ethylenediamine, THF, r.t., 12 h; (j) sodium tert-butoxide, THF, reflux, 3 h.

Compound A32: A solution of A27 (100 mg, 0.19 mmol) was dissolved in THF (3 mL) followed the addition of *N*-Boc-ethylenediamine (91 mg, 0.57 mmol), N-(3-dimethyllaminopropyl)-N′-ethyl-carbodiimide-hydrochloride (218 mg, 1.14 mmol) and 4-dimethyl-aminopyridine (5 mg, 0.04 mmol). The reaction mixture was stirred at room temperature for 12 h. The resulting solution was diluted with ethyl acetate and washed with brine. The organic phase was dried over anhydrous Na_2SO_4 and concentrated in vacuo. The crude residue was purified by column chromatography on a silica gel with DCM-MeOH (10:1) to afford A31 (40 mg, 54%). To a solution of A31 (100 mg, 0.12 mmol) in THF (3 mL), sodium tert-butoxide (71 mg, 0.73 mmol) was added and refluxed for 3 h. The reaction mixture was cooled to room temperature and neutralized with acetic acid. After stirring for 30 min, 6 M NaOH solution was added to the reaction until the pH = 10. The resulting mixture was diluted with ethyl acetate and washed with brine. The organic phase was dried over anhydrous Na_2SO_4 and concentrated under reduced pressure. The crude residue was purified by silica gel column chromatography (10:1, DCM-MeOH) to provide an amorphous powder A32 (40 mg, 54%). ^{1}H NMR (400 MHz, DMSO-d_6, δ): 8.21 (m, 1H, -NH), 8.07 (m, 1H, -NH), 6.99 (s, 1H, Ar-H), 6.51 (s, 1H, Ar-H), 5.22 (t, 1H, J = 7.5 Hz, -CH=), 5.18 (m, 2H, 2×-CH=), 4.77 (s, 2H, -CH_2-), 4.67 (s, 2H, -CH_2-), 4.03 (d, 2H, J = 6.0 Hz, -CH_2-), 3.77 (s, 3H, -OCH_3), 3.37 (m, 2H, -CH_2-), 3.33 (m, 2H, -CH_2-), 3.18 (m, 4H, 2×-CH_2-), 2.64 (m, 2H, -CH_2-), 1.78 (s, 3H, CH_3), 1.73 (s, 3H, CH_3), 1.62 (s, 6H, 2×CH_3); ^{13}C NMR (150 MHz, DMSO-d_6, δ): 181.6, 167.2, 167.0, 161.8, 159.0, 156.9, 154.7, 154.5, 143.9, 136.1, 131.0, 130.8, 123.3, 122.2, 111.3, 111.1, 103.3, 100.1, 90.4, 67.6, 67.4, 62.6, 61.5, 60.6, 2×52.1, 25.7, 25.6, 25.6, 21.1, 18.1, 17.8.

(A)

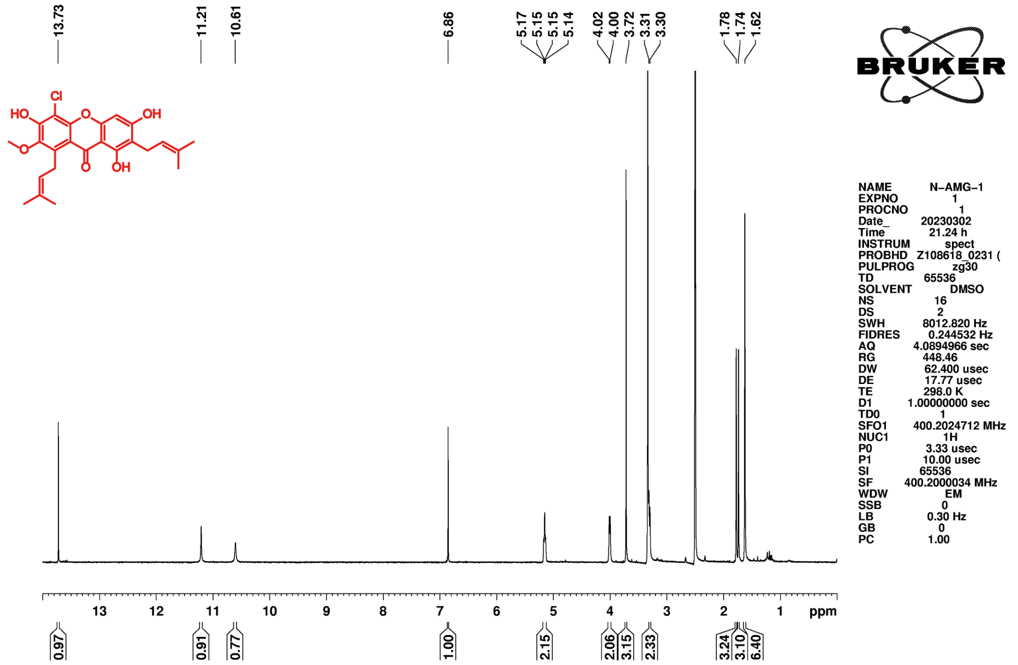

(B)

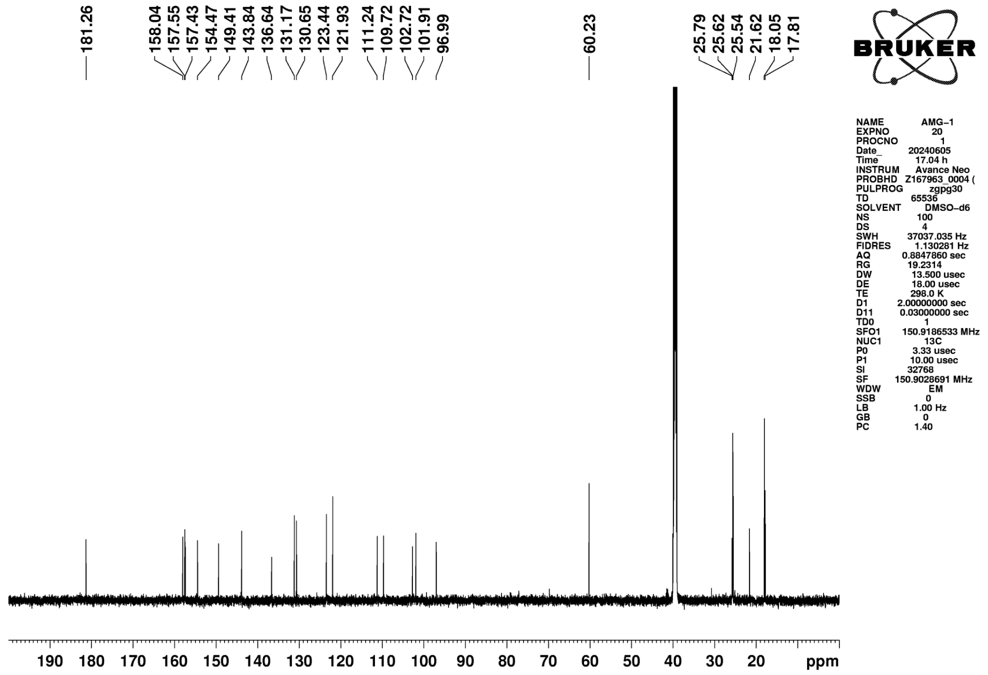

Figure S1 ^{1}H NMR (400 MHz, DMSO$_{d6}$) (A) and ^{13}C NMR spectrum (150 MHz, DMSO$_{d6}$) (B) of A1.

(A)

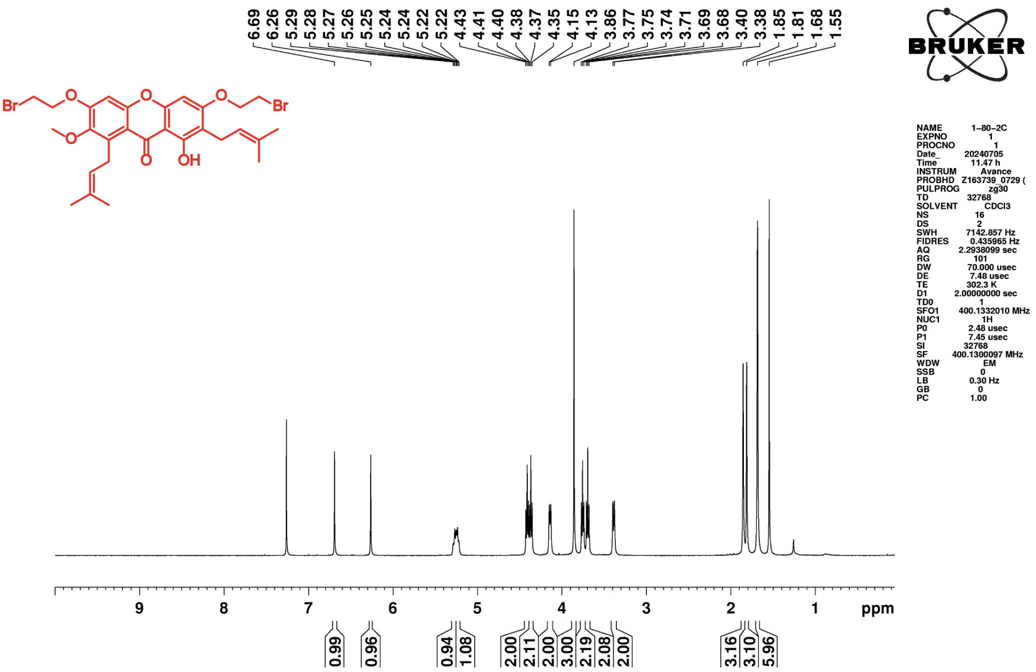

(B)

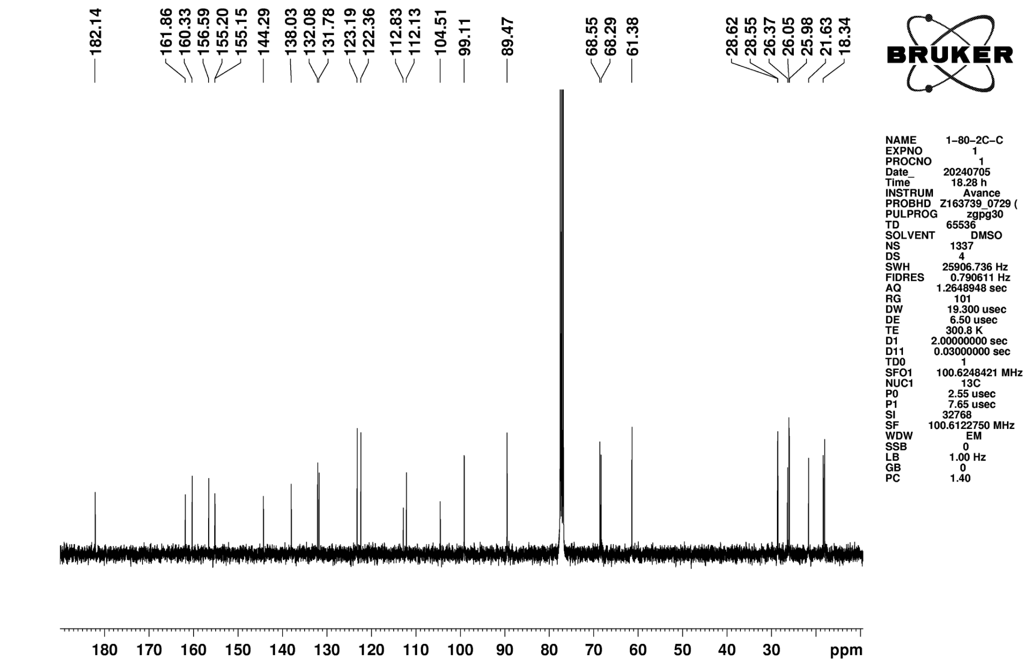

Figure S2 ^{1}H NMR (400 MHz, $CDCl_3$) (A) and ^{13}C NMR (100 MHz, $CDCl_3$) (B) spectrum of A3.

(A)

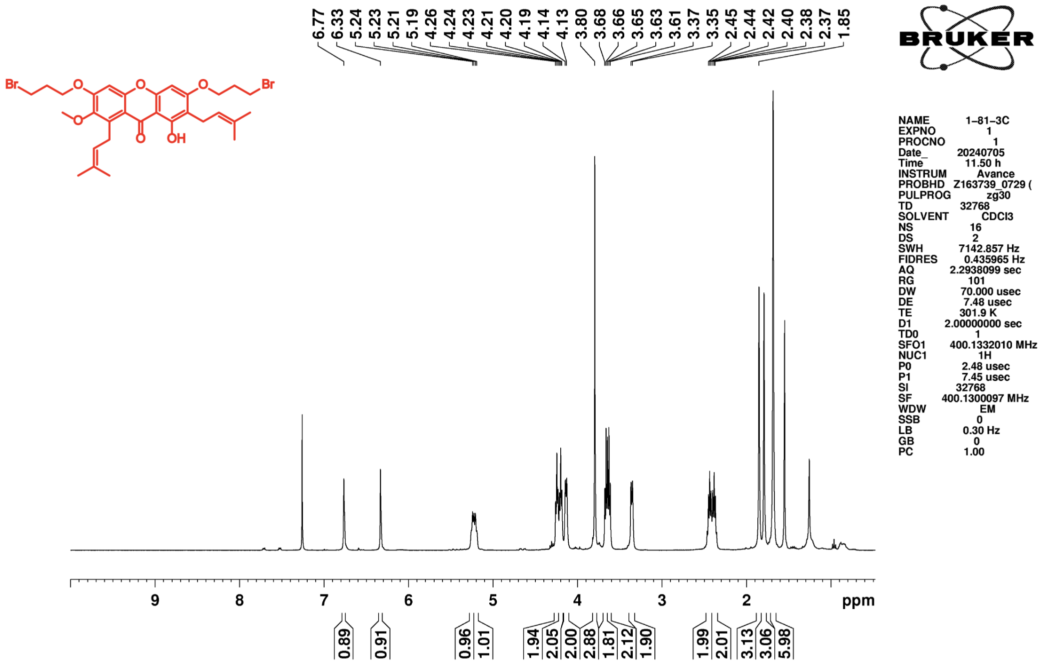

(B)

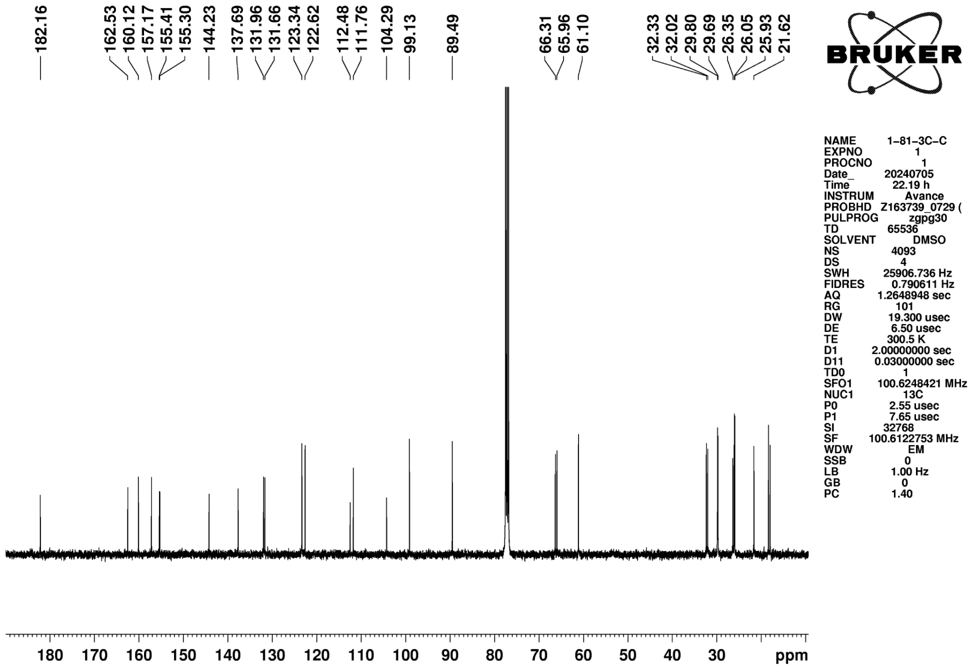

Figure S3 ^{1}H NMR (400 MHz, $CDCl_3$) (A) and ^{13}C NMR (100 MHz, $CDCl_3$) (B) spectrum of A4.

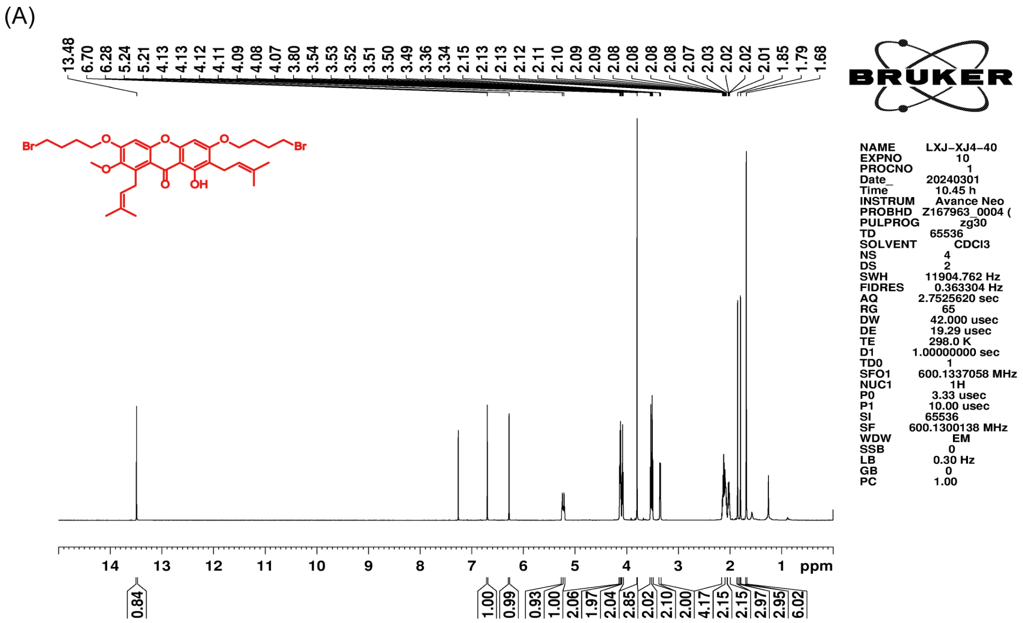

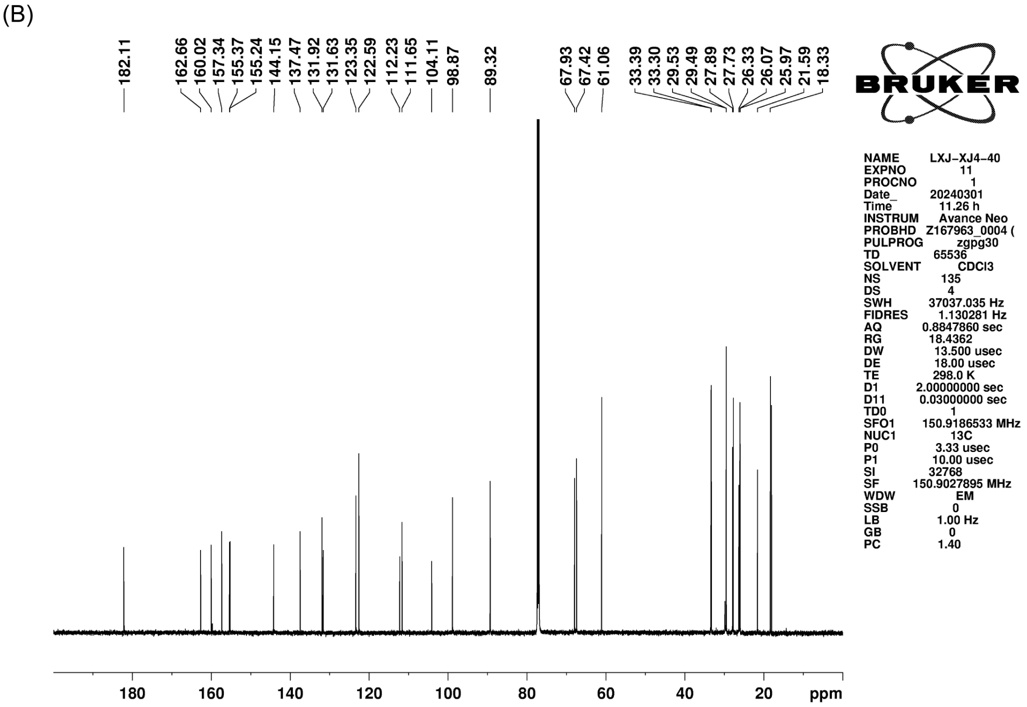

Figure S4 ^{1}H NMR (400 MHz, $CDCl_3$) (A) and ^{13}C NMR (150 MHz, $CDCl_3$) (B) spectrum of A5.

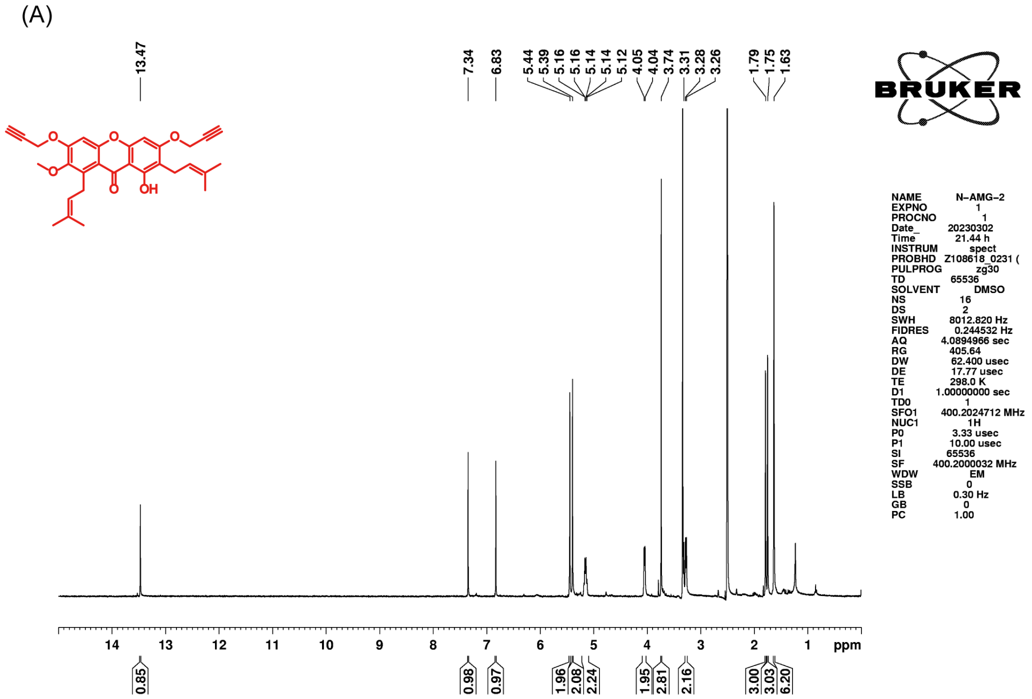

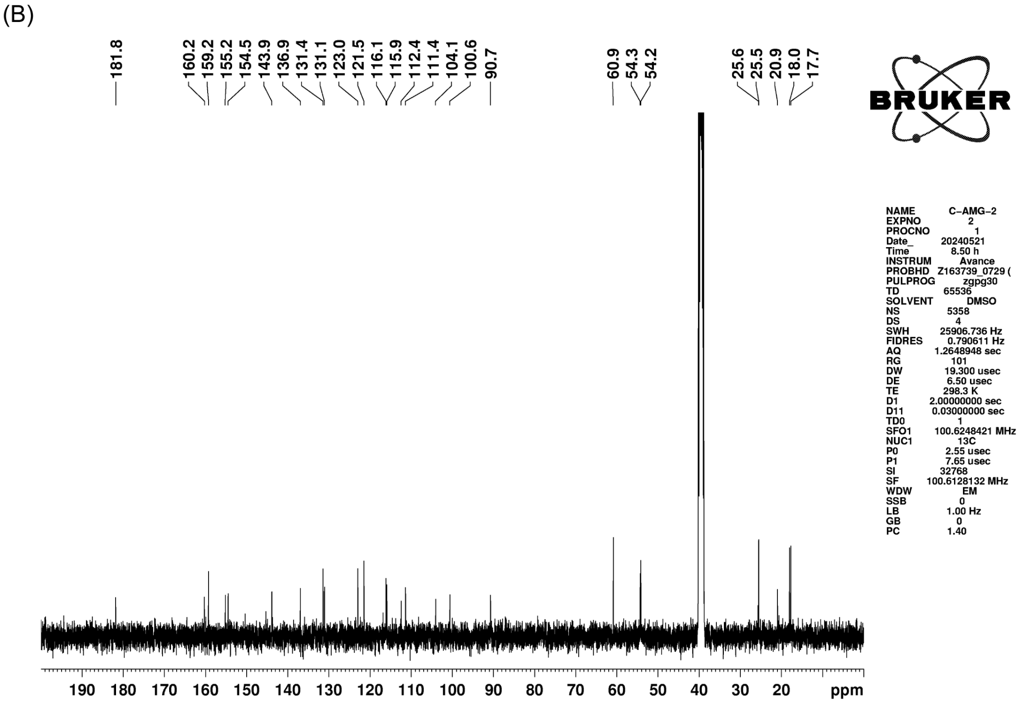

Figure S5 ^{1}H NMR (400 MHz, DMSO-d_6) (A) and ^{13}C NMR (150 MHz, DMSO-d_6) (B) spectrum of A6.

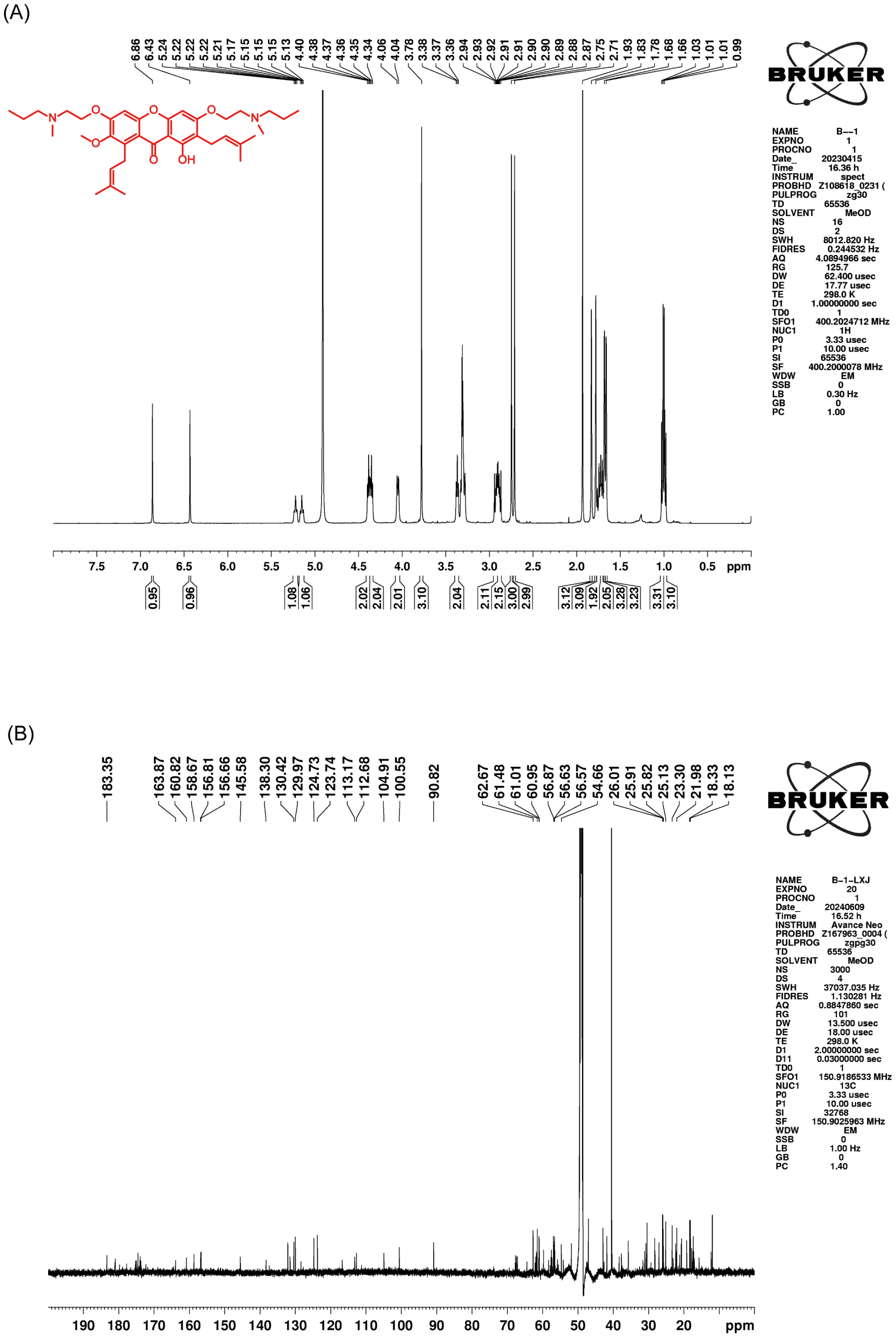

Figure S6 ^{1}H NMR (400 MHz, CD_3OD) (A) and ^{13}C NMR (150 MHz, CD_3OD) (B) spectrum of A7.

(A)

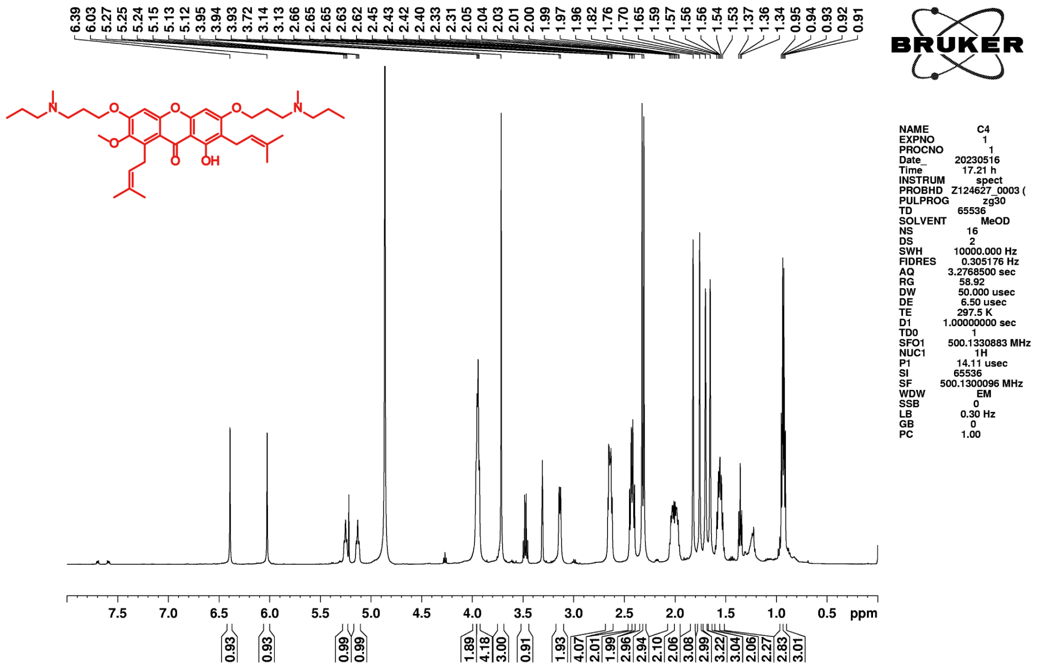

(B)

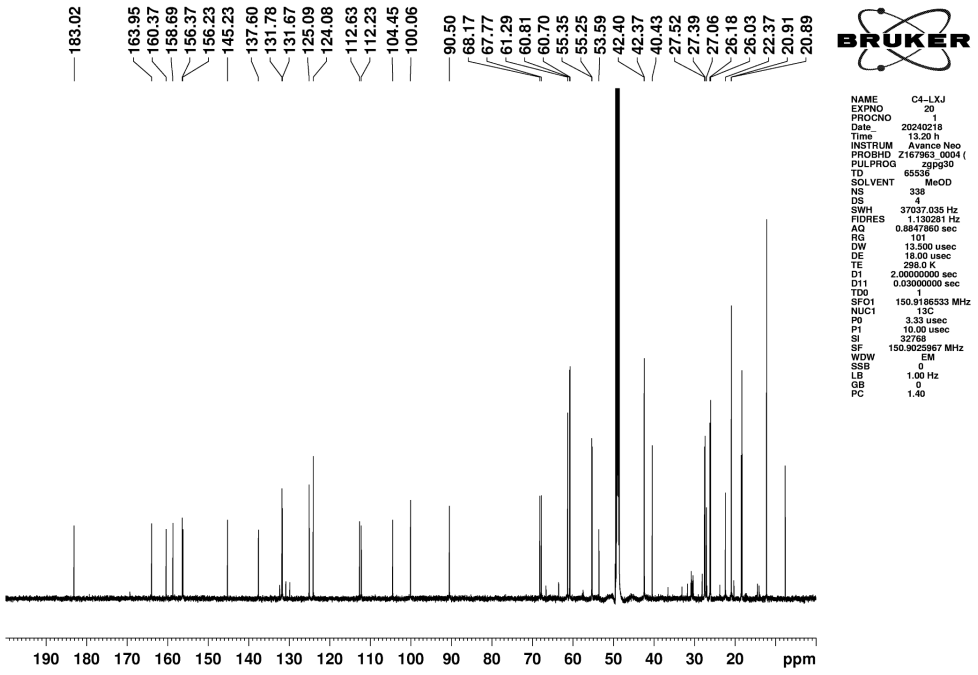

Figure S7 ^{1}H NMR (500 MHz, CD_3OD) (A) and ^{13}C NMR (150 MHz, CD_3OD) (B) spectrum of A8.

(A)

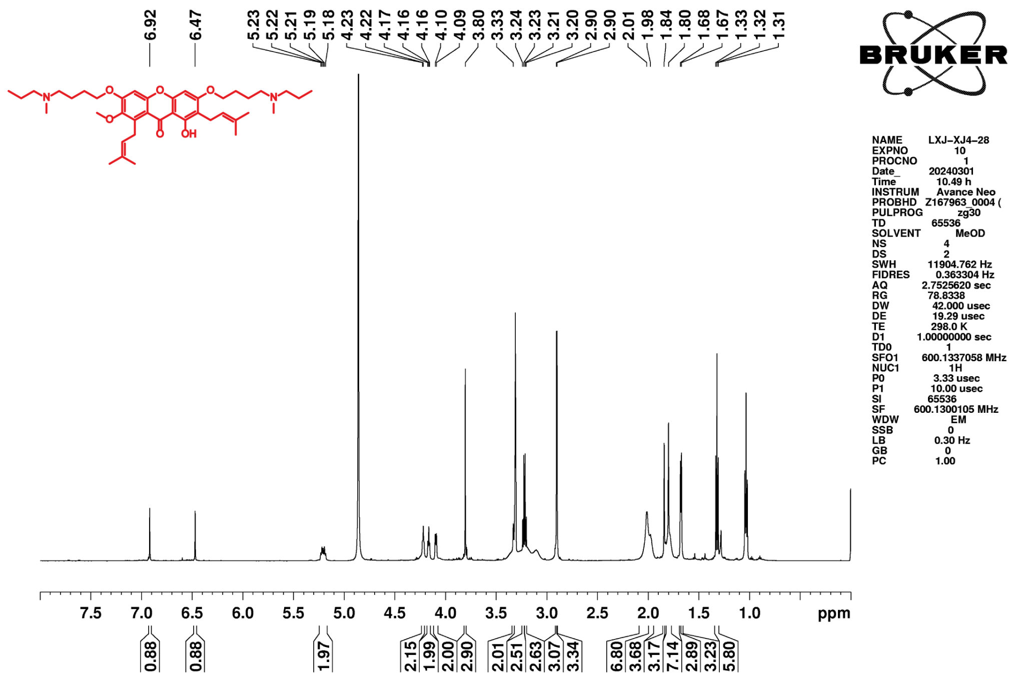

(B)

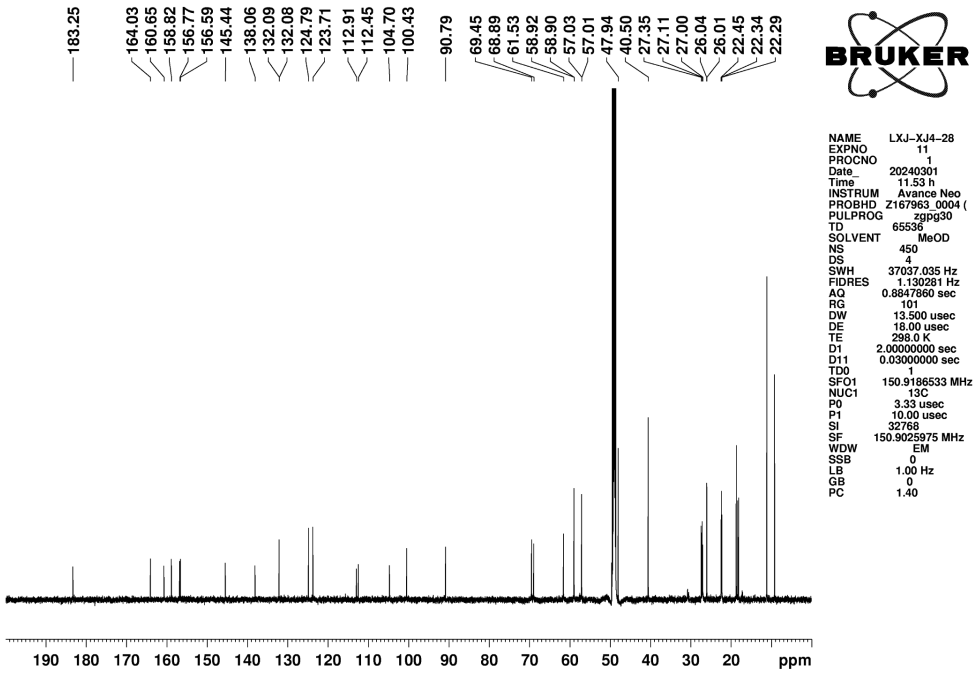

Figure S8 ^{1}H NMR (600 MHz, CD_3OD) (A) and ^{13}C NMR (150 MHz, CD_3OD) (B) spectrum of A9.

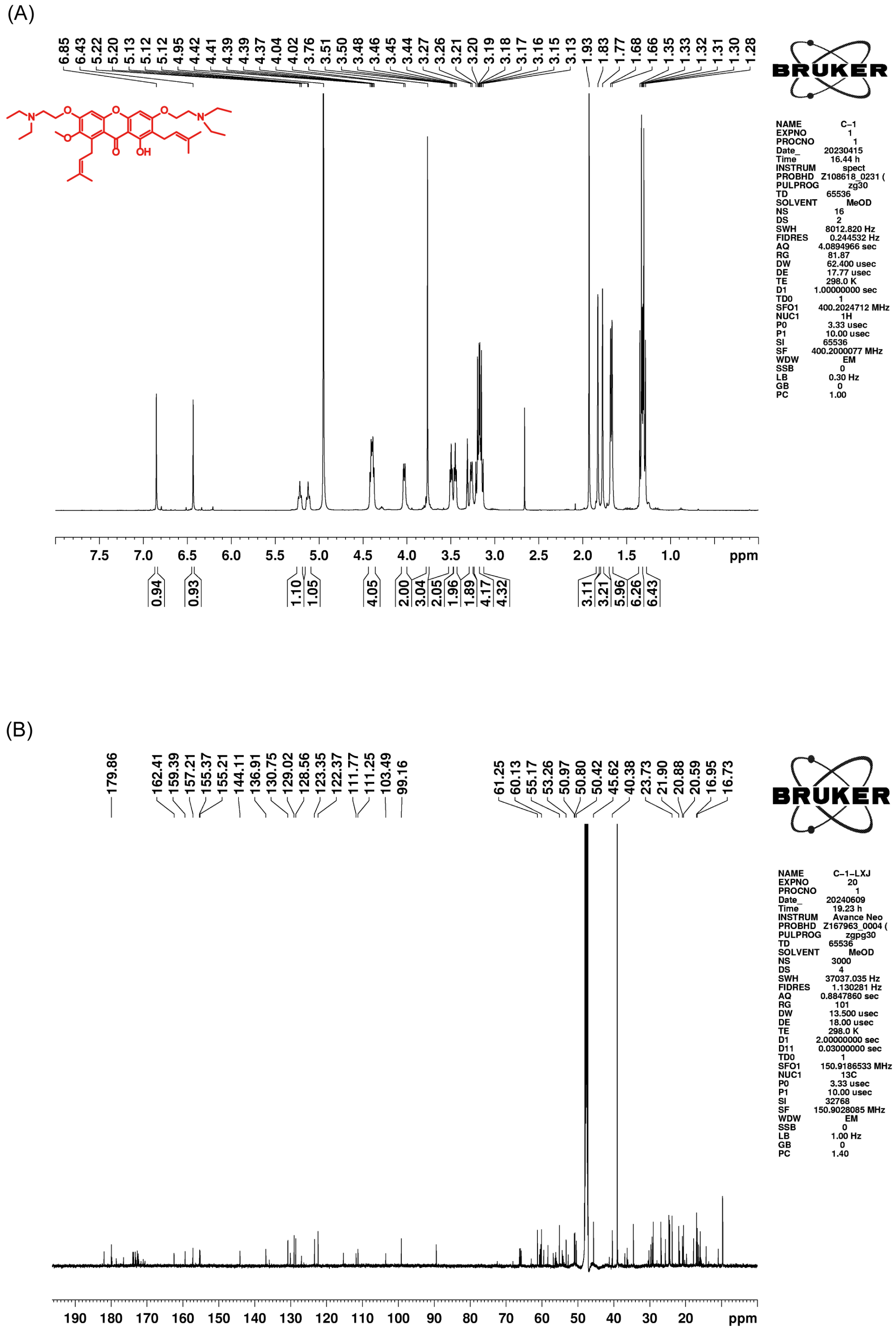

Figure S9 ^{1}H NMR (400 MHz, CD_3OD) (A) and ^{13}C NMR (150 MHz, CD_3OD) (B) spectrum of A10.

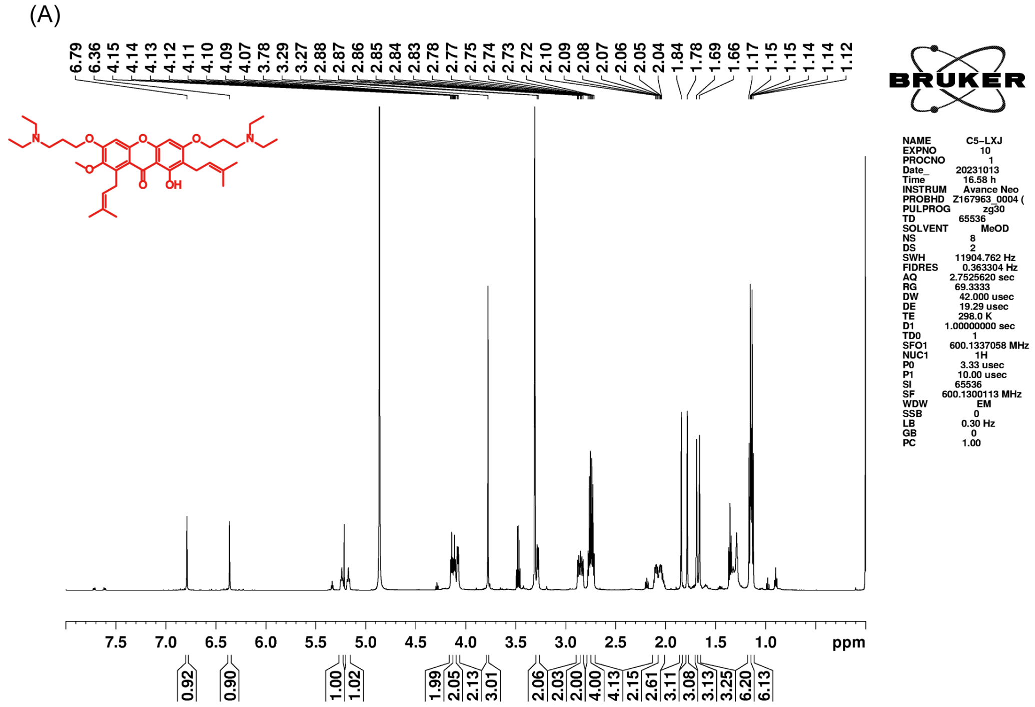

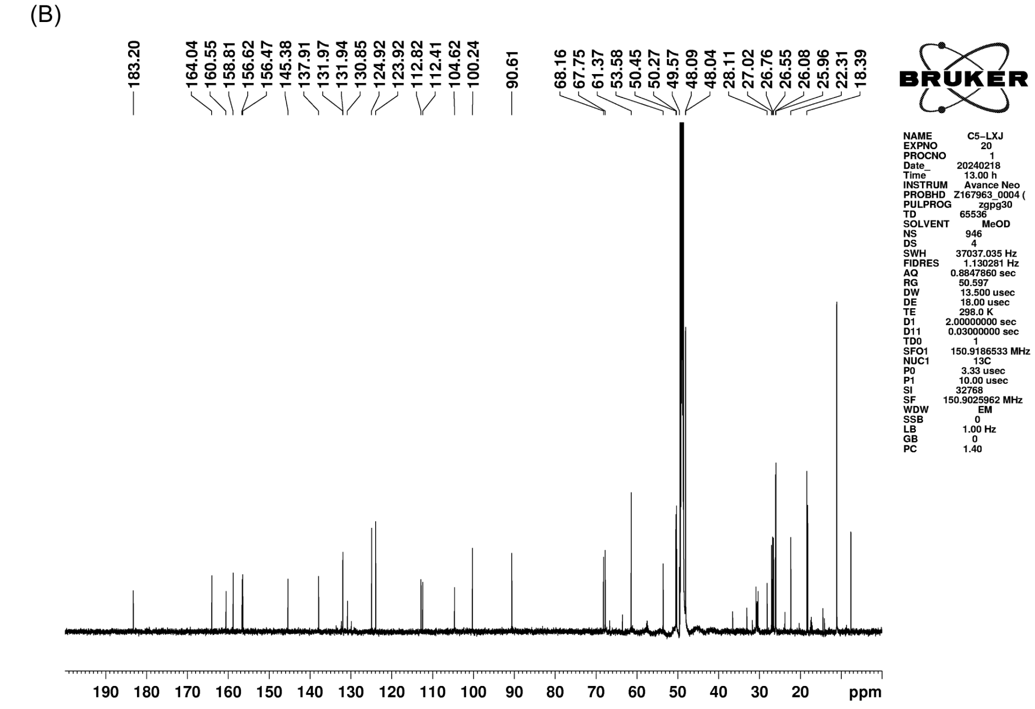

Figure S10 ^{1}H NMR (600 MHz, CD_3OD) (A) and ^{13}C NMR (150 MHz, CD_3OD) (B) spectrum of A11.

(A)

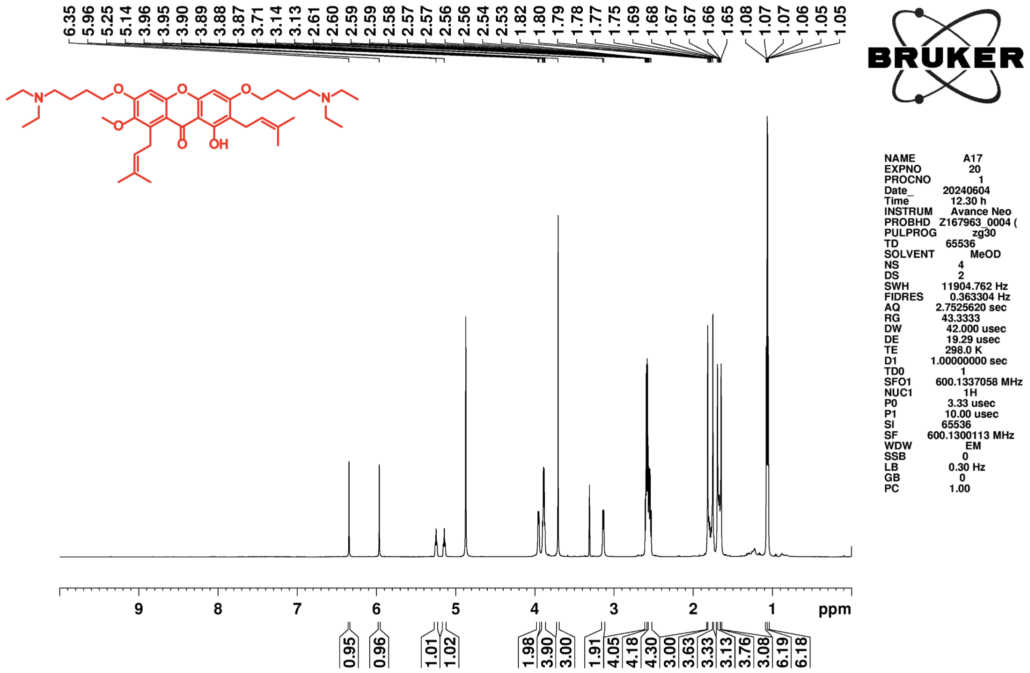

564

(B)

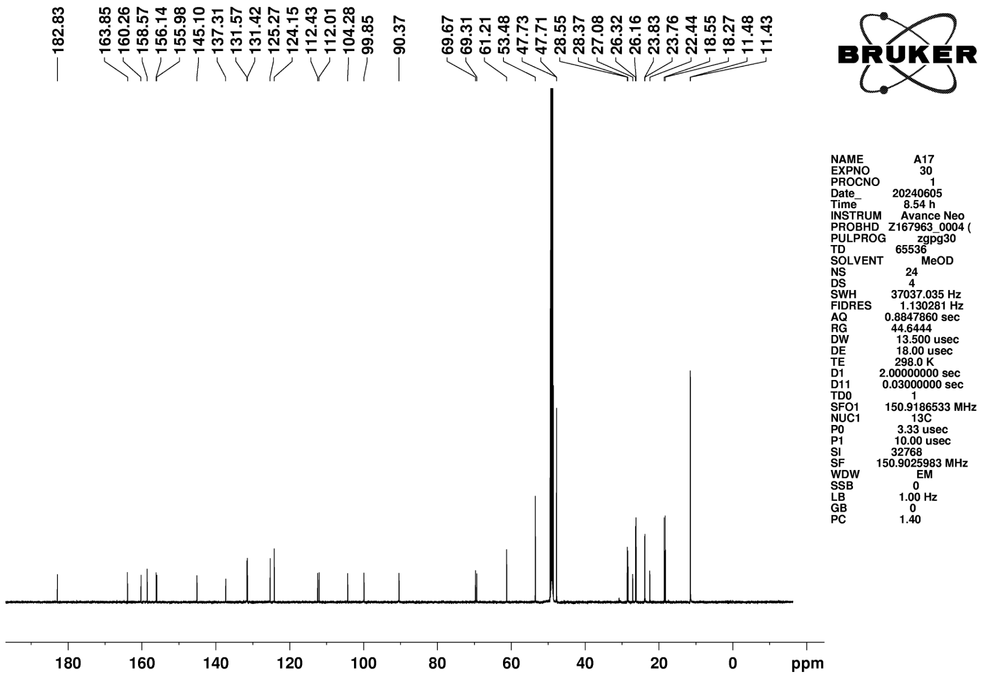

565

566 **Figure S11 ^{1}H NMR (600 MHz, CD_3OD) (A) and ^{13}C NMR (150 MHz, CD_3OD) (B) spectrum of**
567 **A12.**

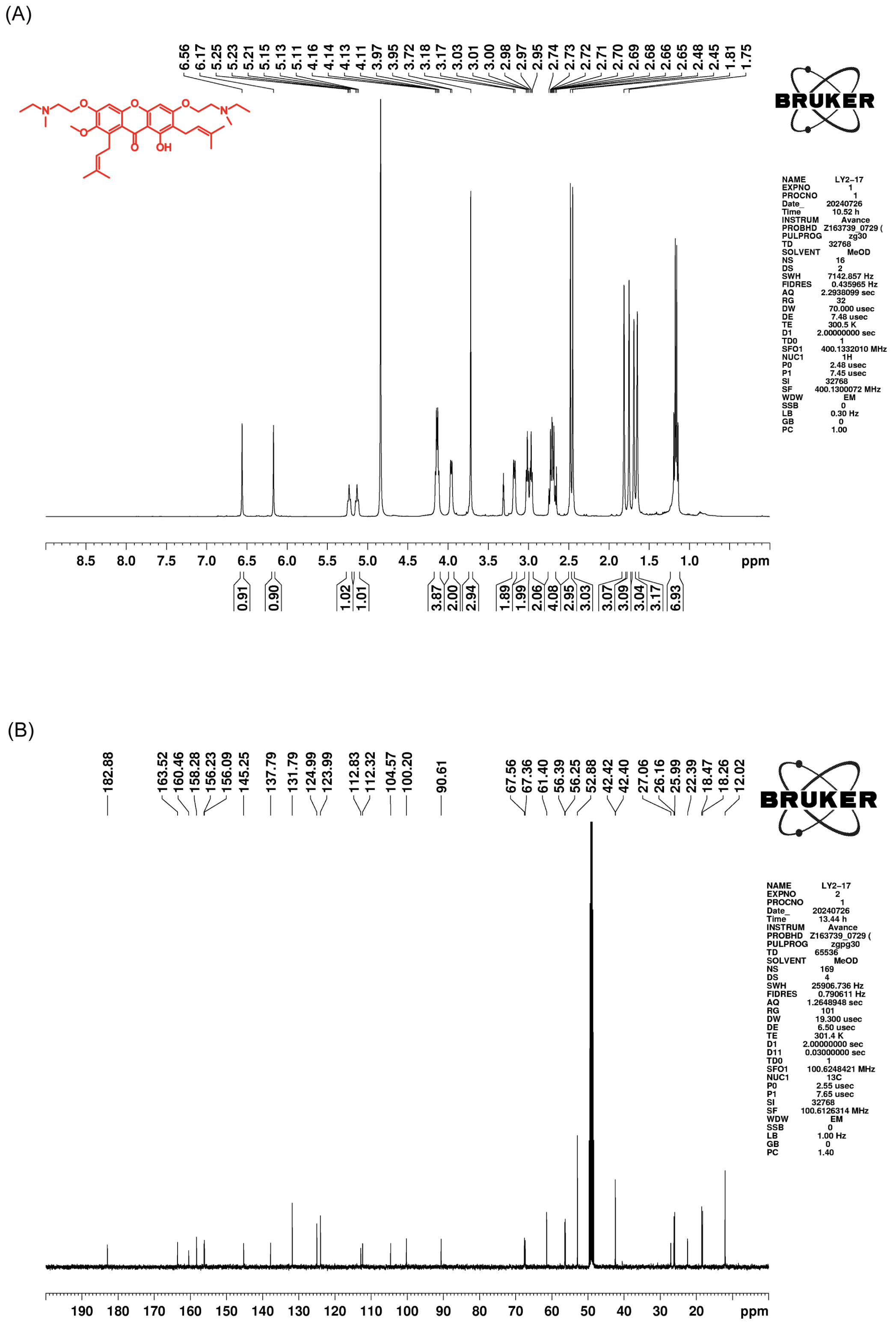

Figure S12 ^{1}H **NMR (400 MHz, CD_3OD) (A) and** ^{13}C **NMR (100 MHz, CD_3OD) (B) spectrum of A13.**

(A)

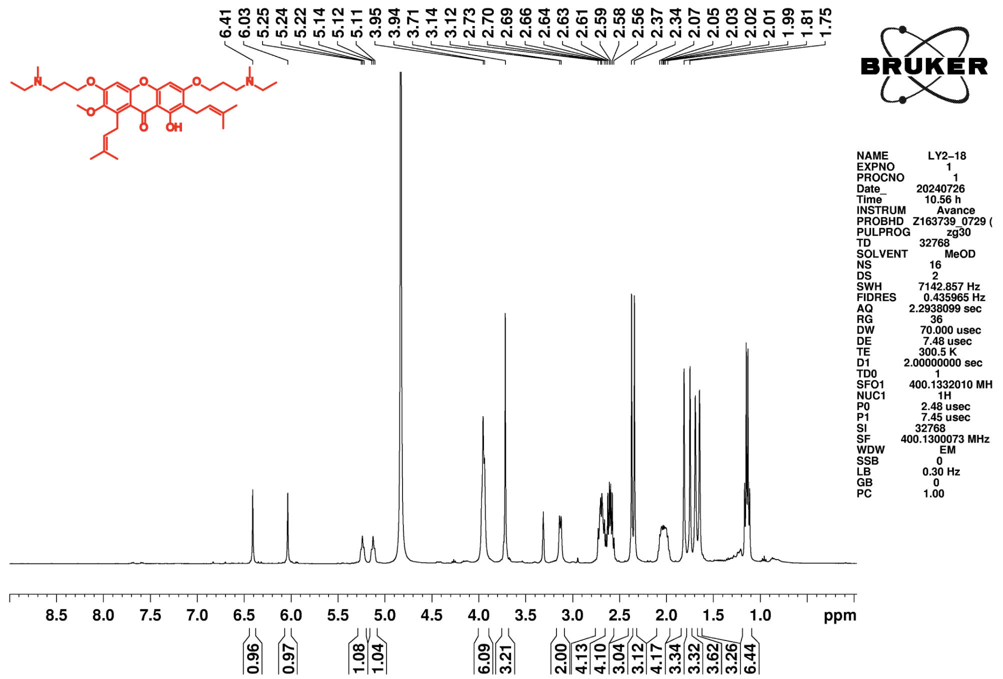

(B)

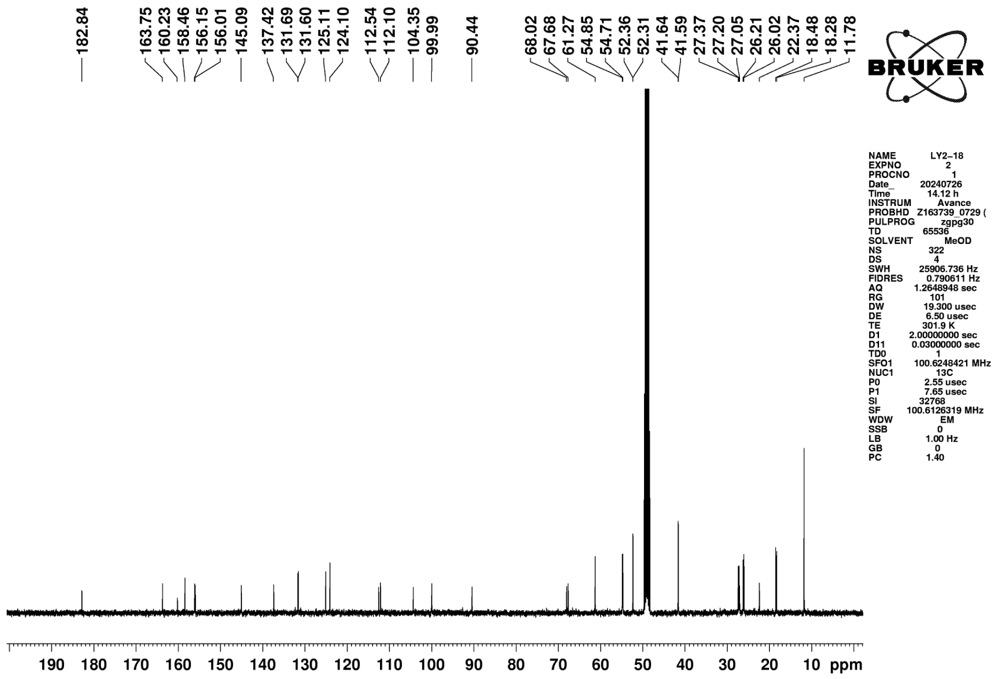

Figure S13 ^{1}H NMR (400 MHz, CD_3OD) (A) and ^{13}C NMR (100 MHz, CD_3OD) (B) spectrum of A14.

(A)

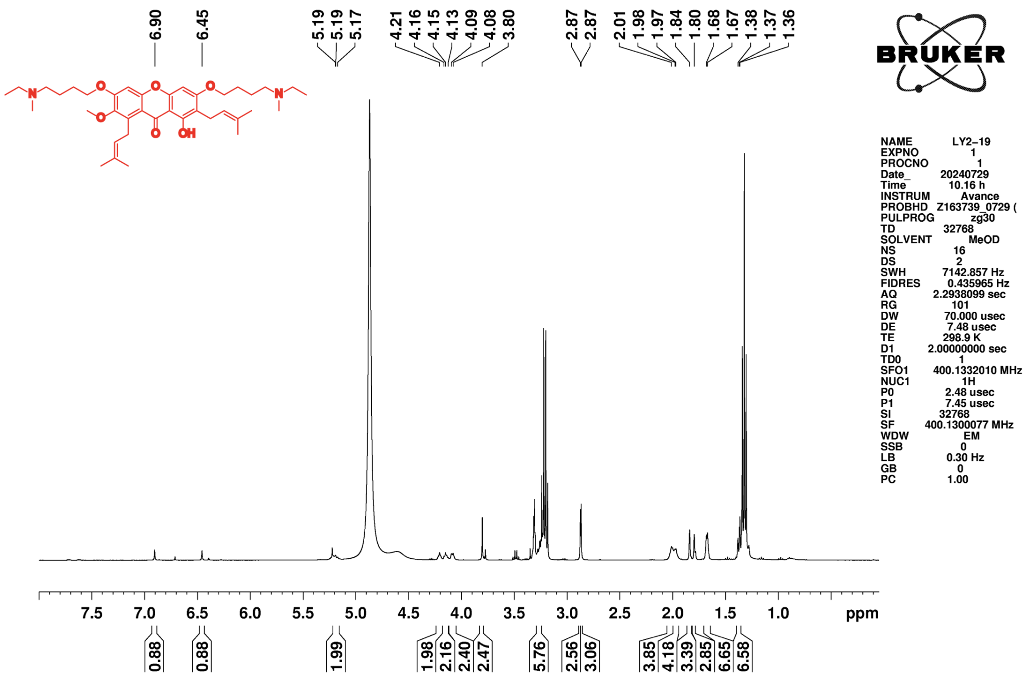

(B)

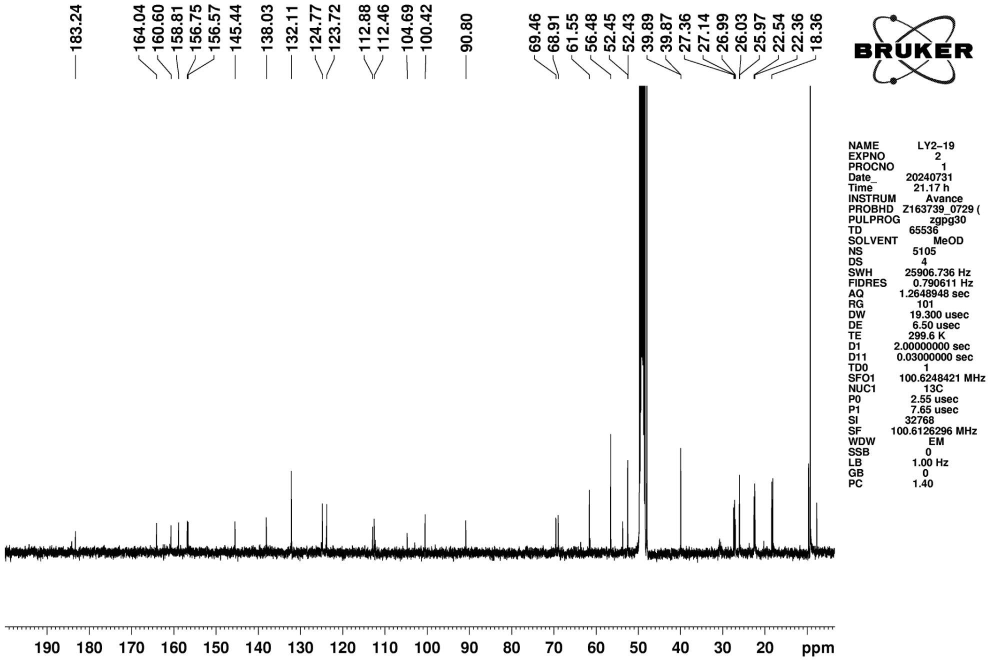

Figure S14 ^{1}H NMR (400 MHz, CD_3OD) (A) and ^{13}C NMR (100 MHz, CD_3OD) (B) spectrum of A15.

(A)

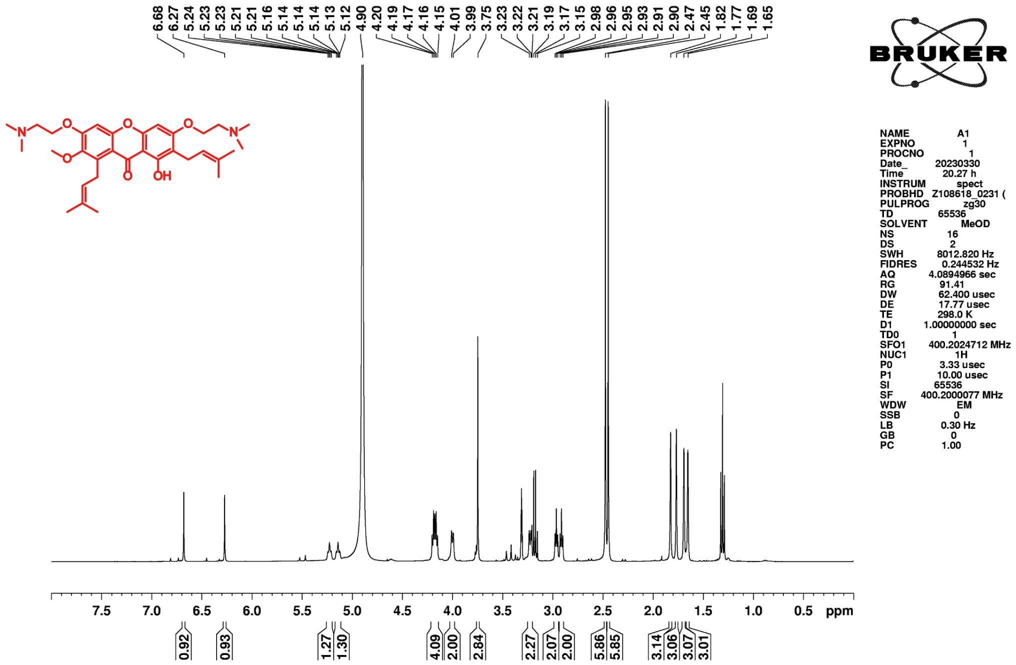

Figure S15 ^{1}H NMR spectrum (400 MHz, CD_3OD) of A16.

(A)

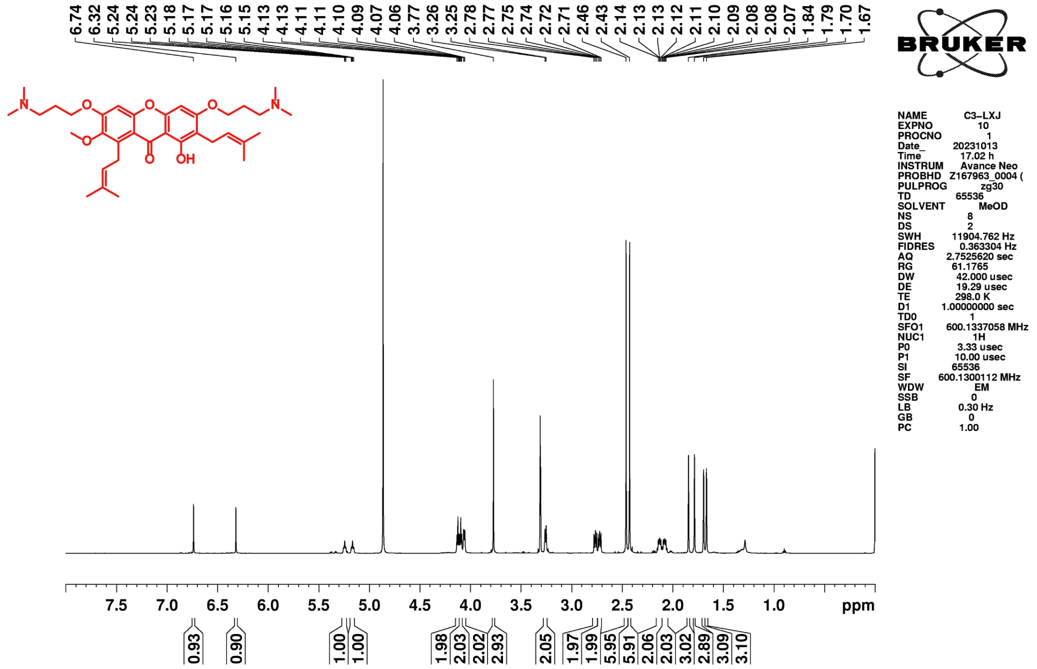

(B)

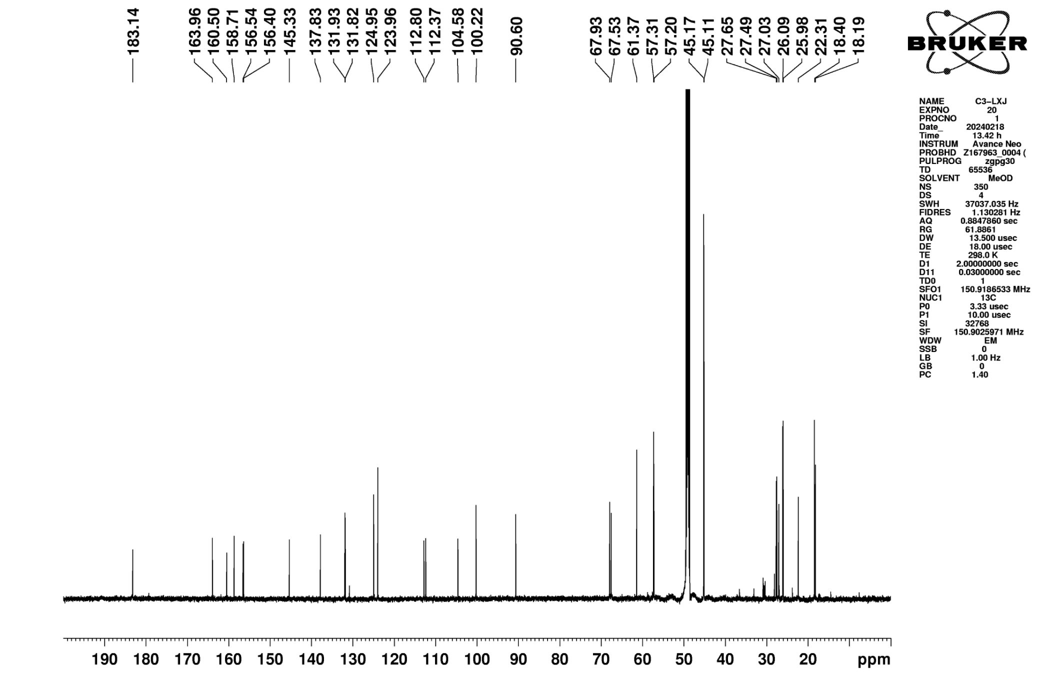

Figure S16 ^{1}H NMR (600 MHz, CD_3OD) (A) and ^{13}C NMR (150 MHz, CD_3OD) (B) spectrum of A17.

(A)

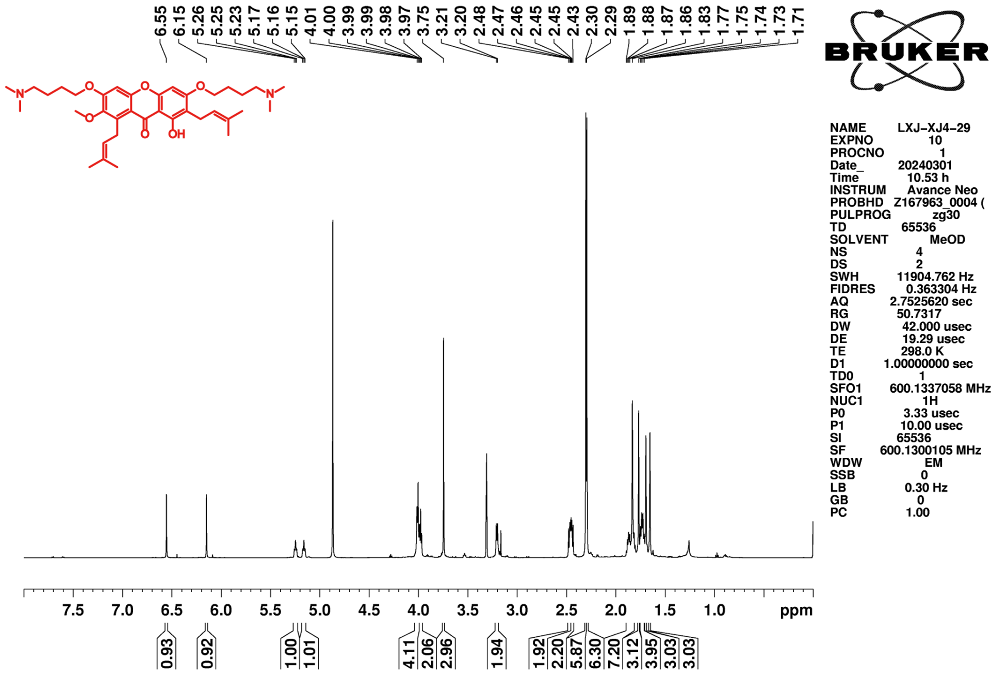

(B)

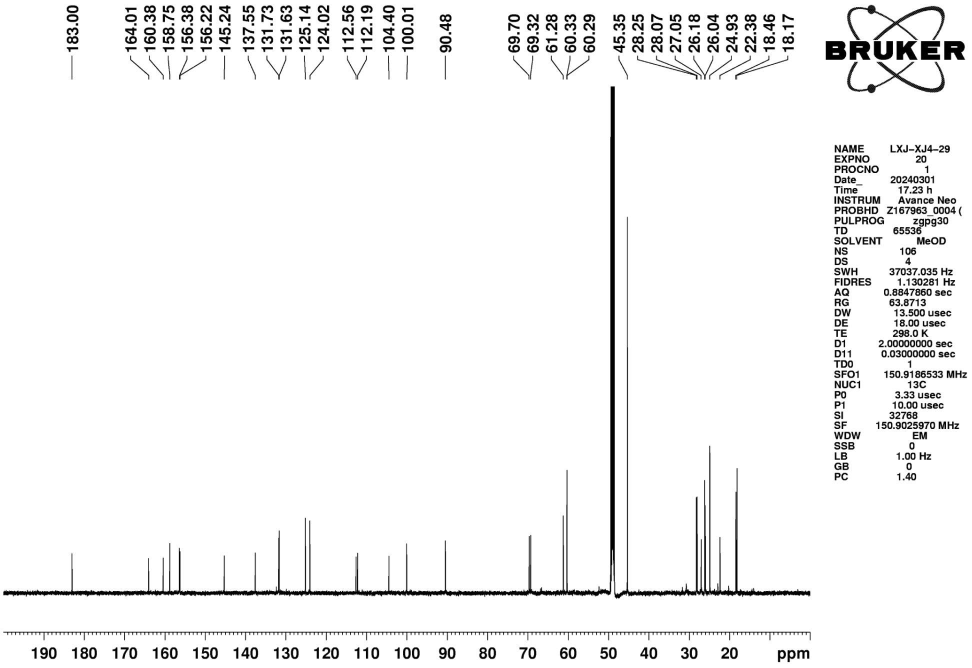

Figure S17 ^{1}H NMR (600 MHz, CD_3OD) (A) and ^{13}C NMR (150 MHz, CD_3OD) (B) spectrum of A18.

(A)

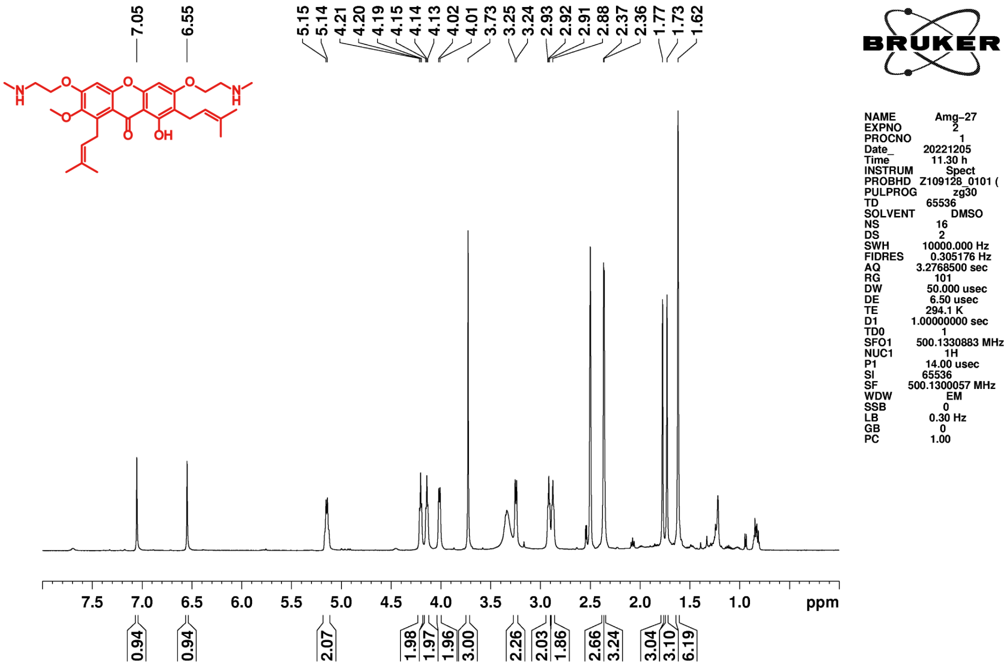

591

592 **Figure S18 ^{1}H NMR spectrum (500 MHz, DMSO-d_6) of A19.**

593

(A)

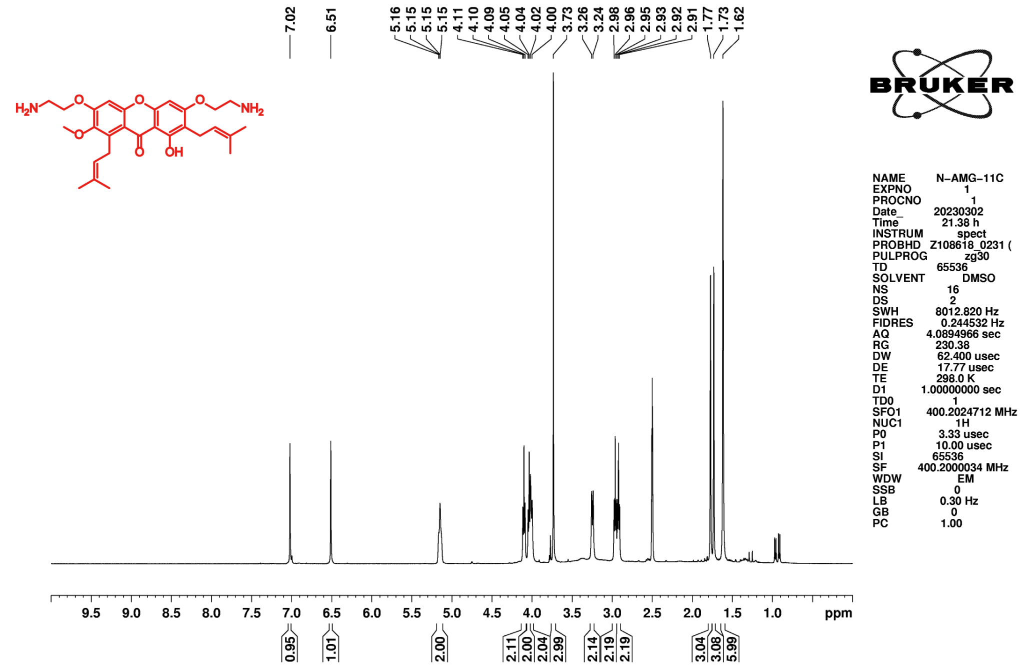

(B)

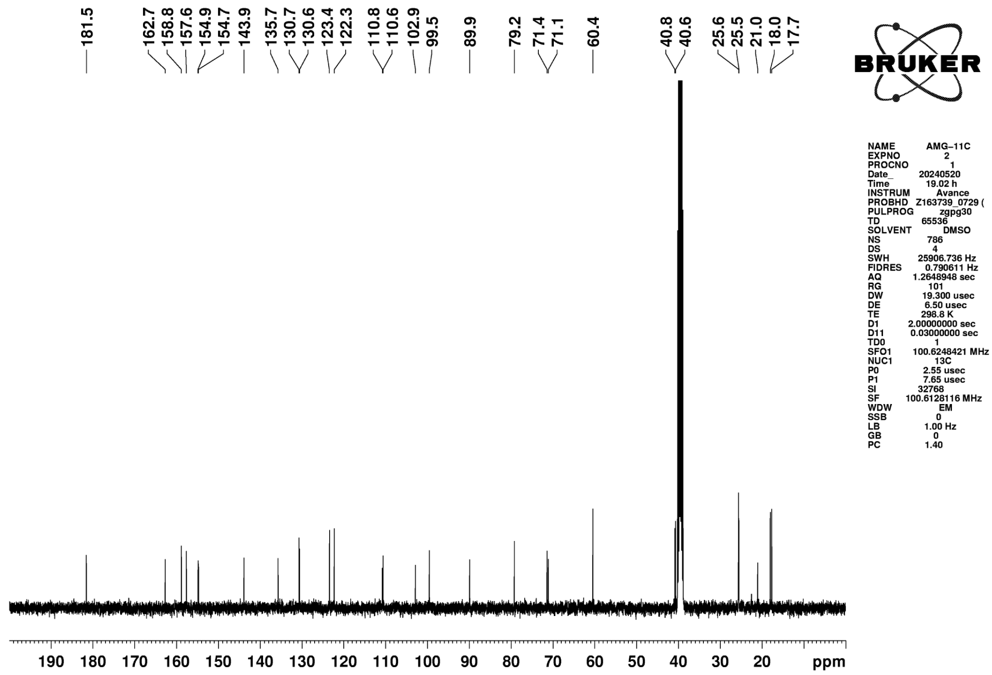

Figure S19 ^{1}H NMR (400 MHz, DMSO$_{d6}$) (A) and ^{13}C NMR (150 MHz, DMSO-$_{d6}$) (B) spectrum of A20.

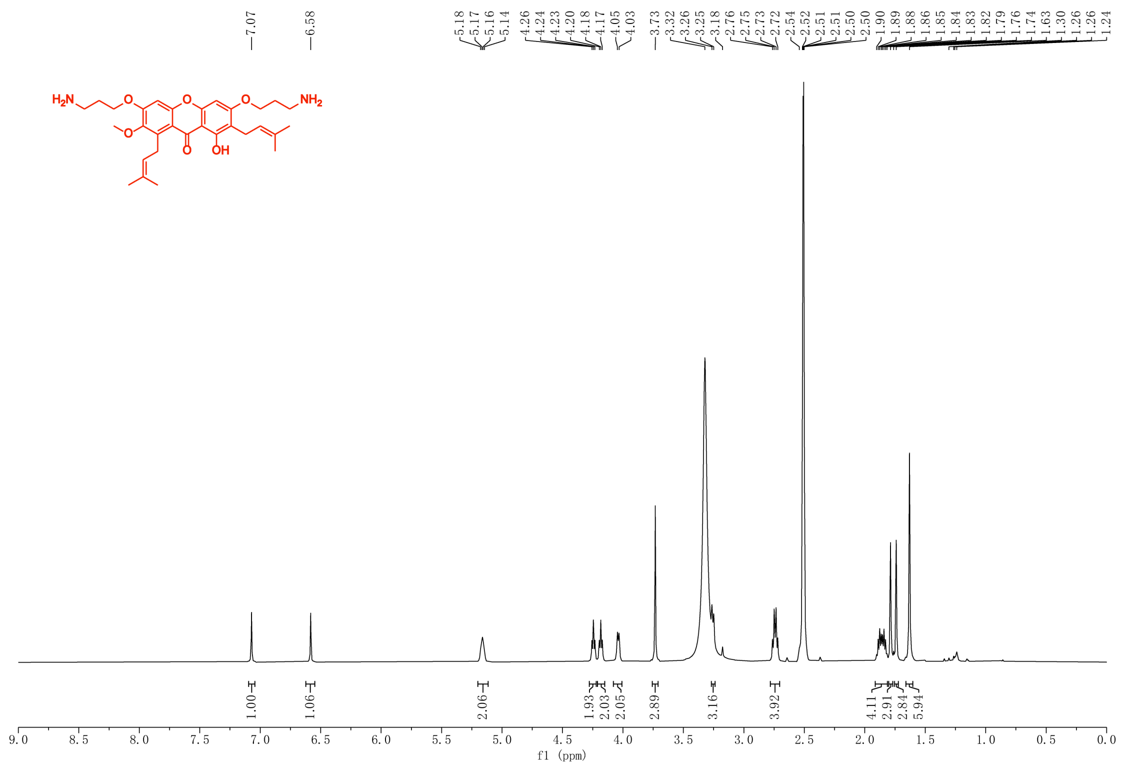

Figure S20 ^{1}H NMR spectrum (400 MHz, DMSO$_{d6}$) of A21.

(A)

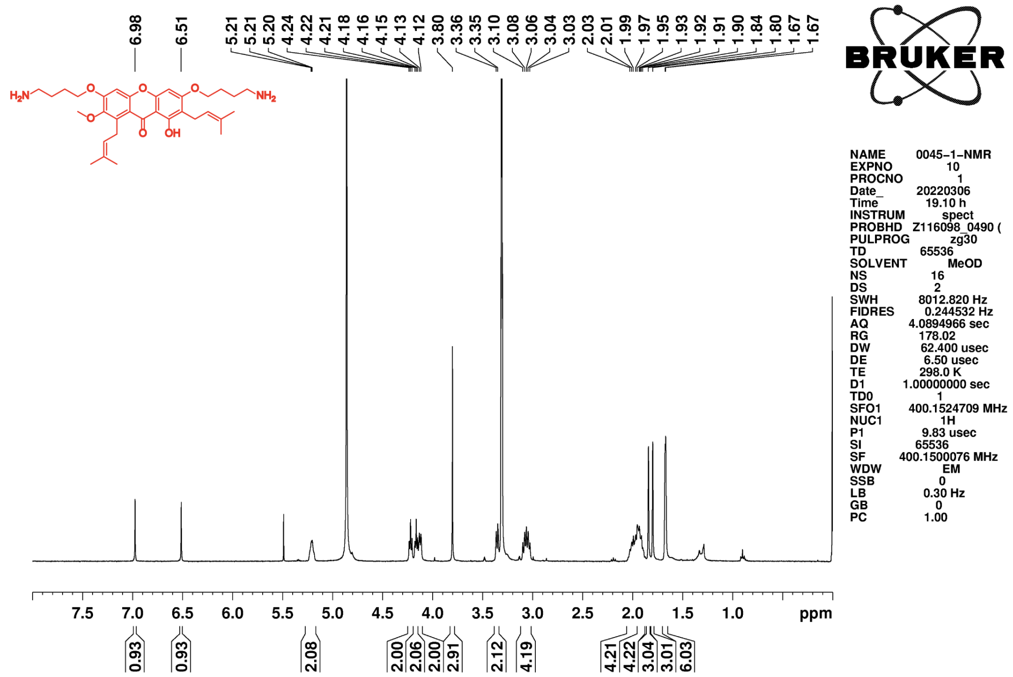

Figure S21 ^{1}H NMR spectrum (400 MHz, CD_3OD) of A22.

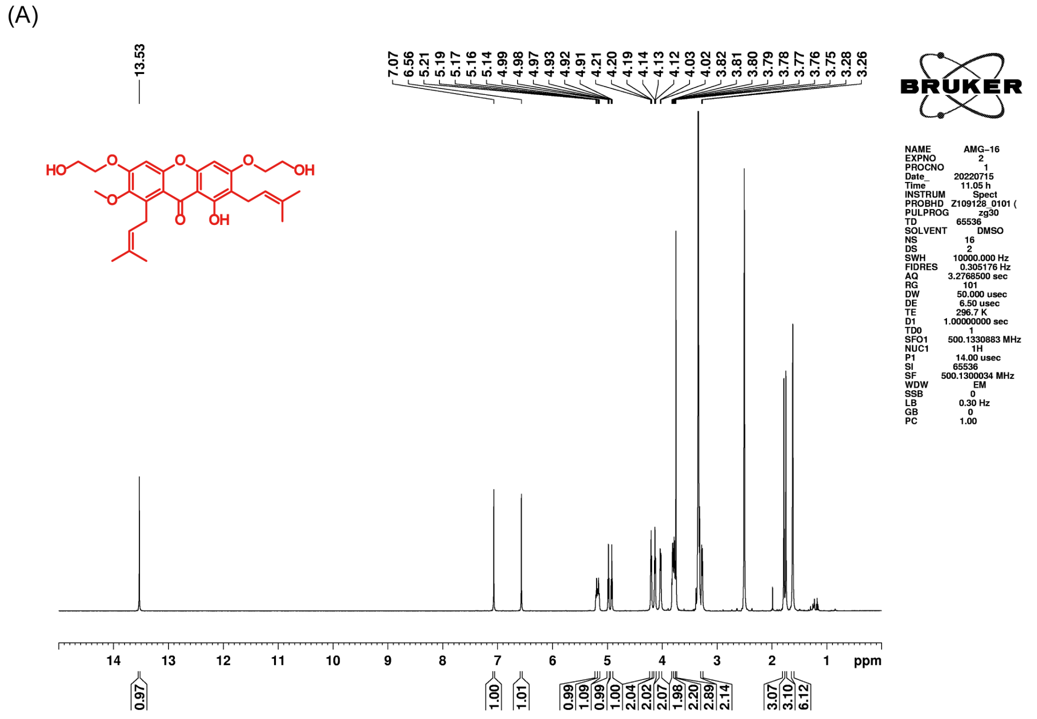

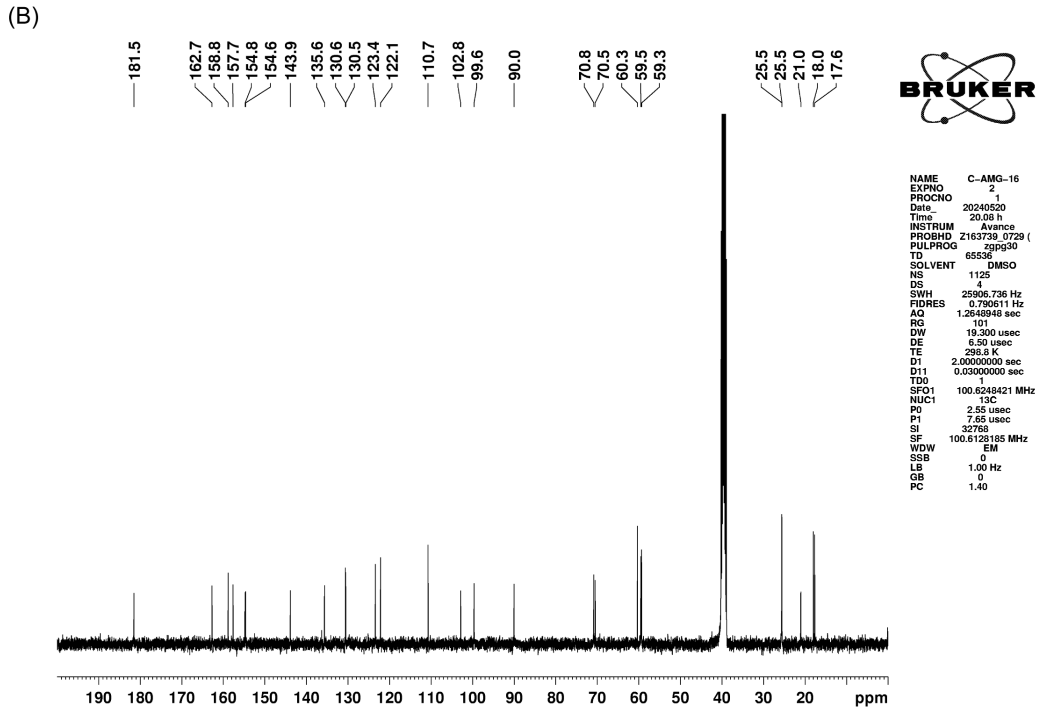

Figure S22 ^{1}H NMR (500 MHz, DMSO-*d6*) (A) and ^{13}C NMR (100 MHz, DMSO-*d6*) (B) spectrum of A23.

(A)

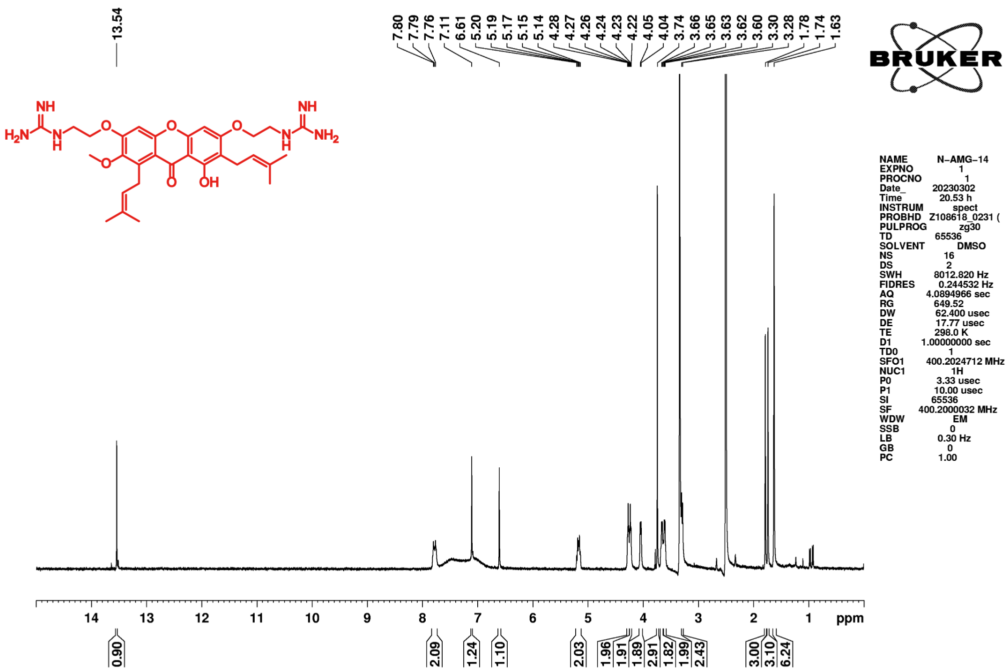

(B)

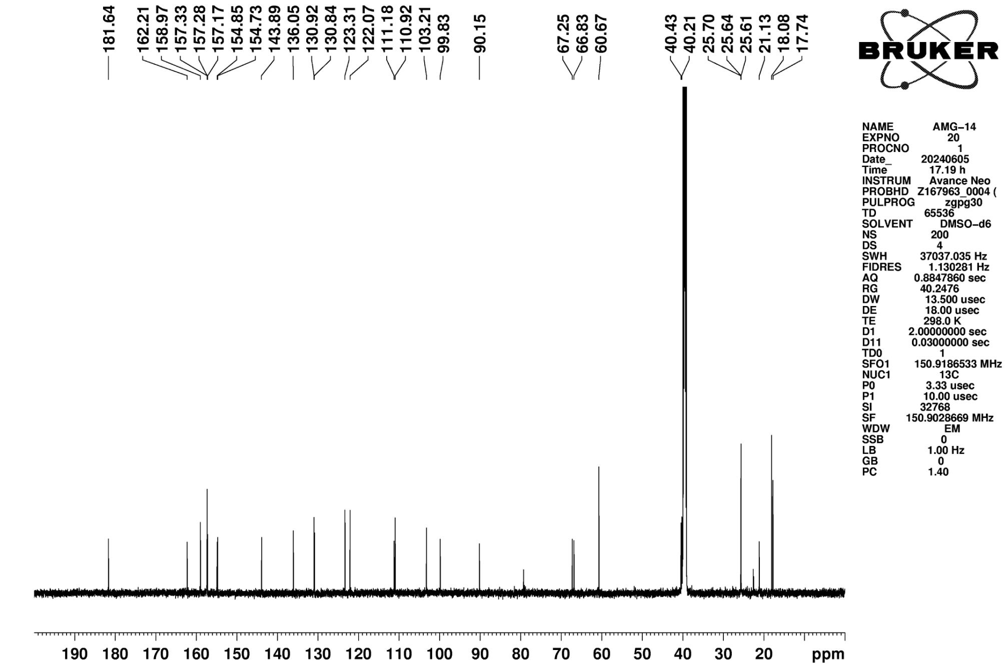

Figure S23 ^{1}H NMR (400 MHz, DMSO-*d6*) (A) and ^{13}C NMR spectrum (150 MHz, DMSO-*d6*) (B) spectrum of A24.

(A)

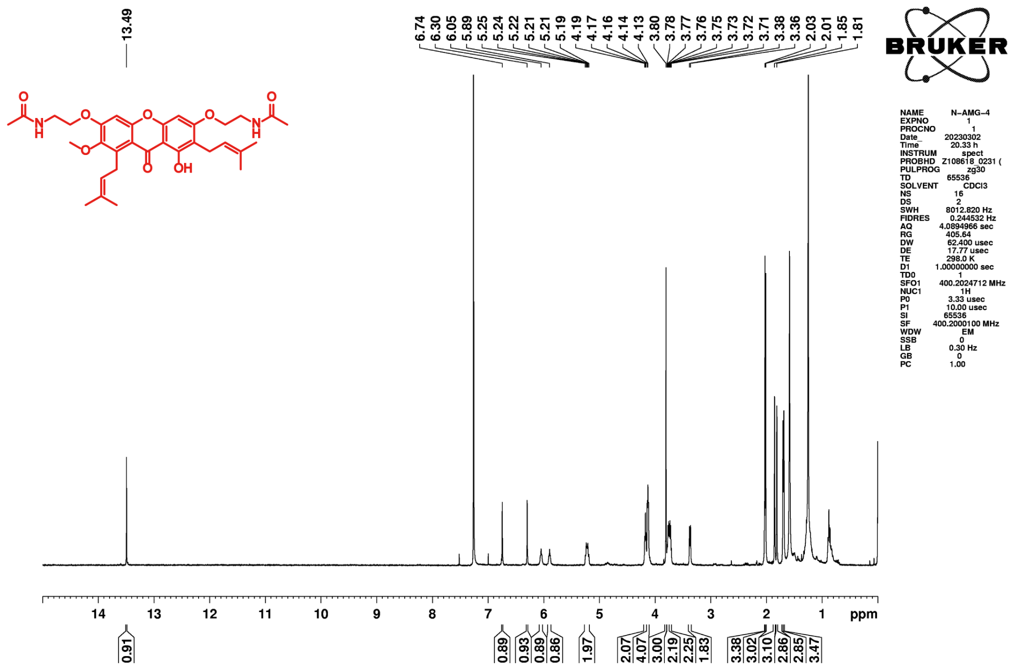

(B)

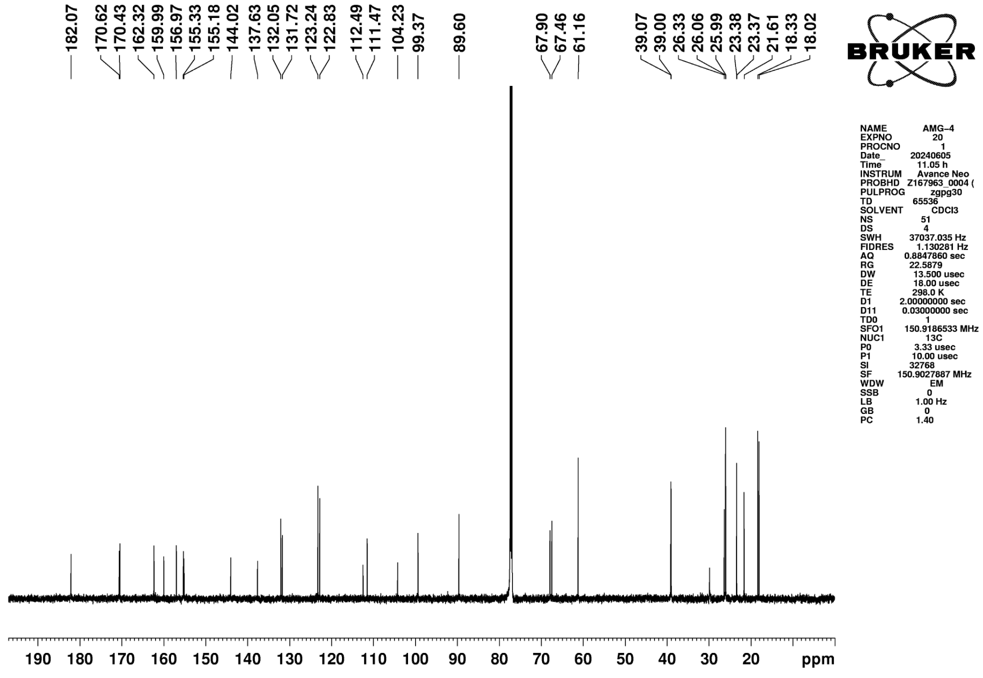

Figure S24 ^{1}H NMR (400 MHz, CDCl$_3$) (A) and ^{13}C NMR spectrum (150 MHz, CDCl$_3$) (B) spectrum of A25.

(A)

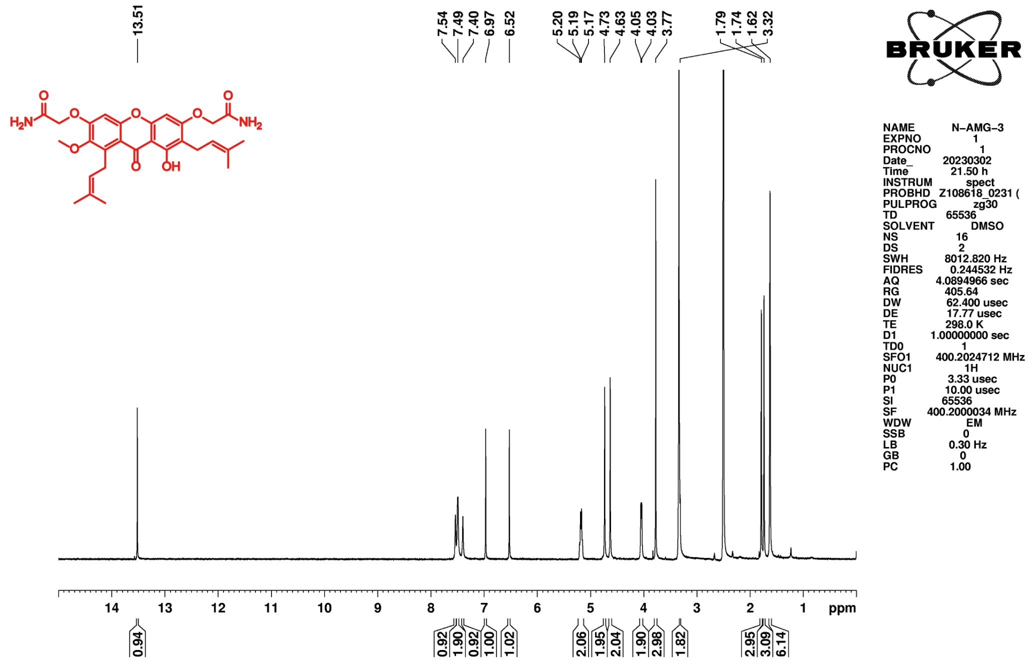

(B)

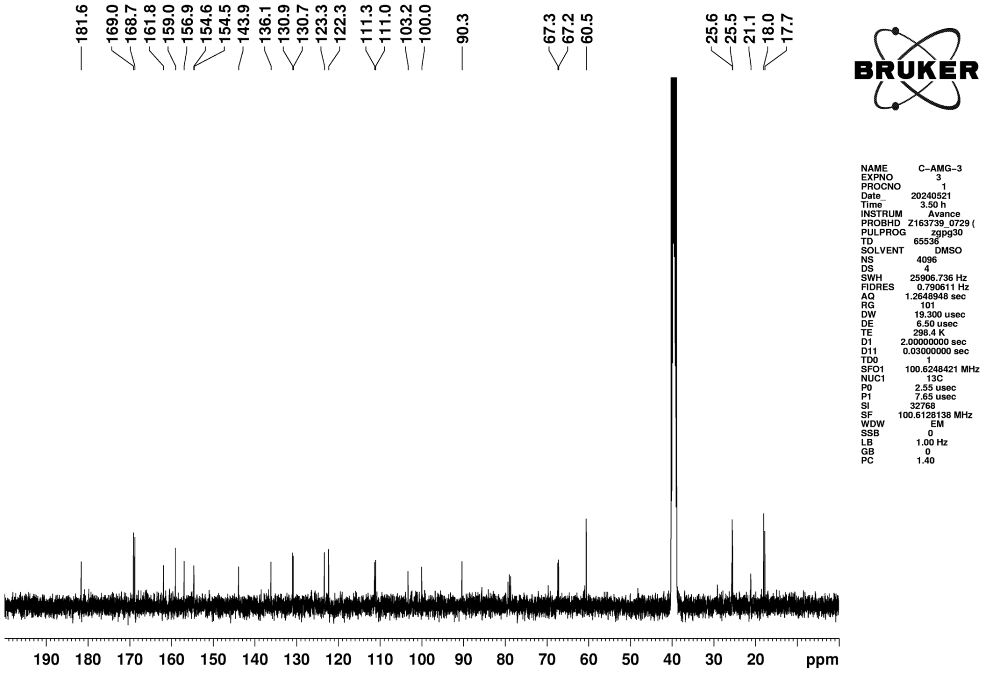

Figure S25 ^{1}H NMR (400 MHz, DMSO-*d6*) (A) and ^{13}C NMR (100 MHz, DMSO-*d6*) (B) spectrum of A28.

(A)

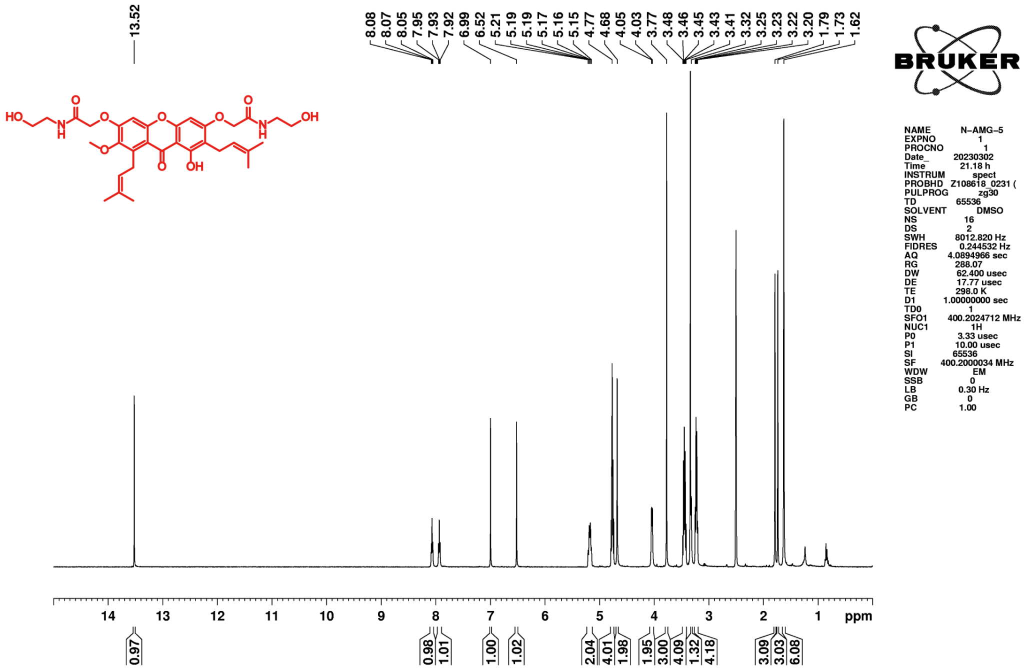

(B)

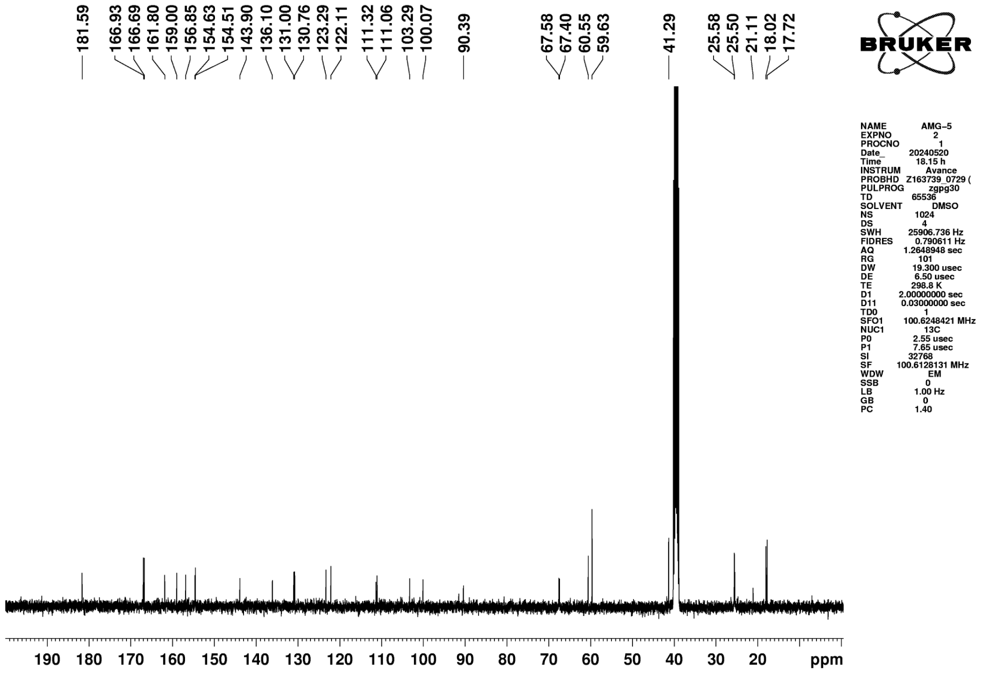

Figure S26 ^{1}H NMR (400 MHz, DMSO-d_6) (A) and ^{13}C NMR (100 MHz, DMSO-d_6) (B) spectrum of A29.

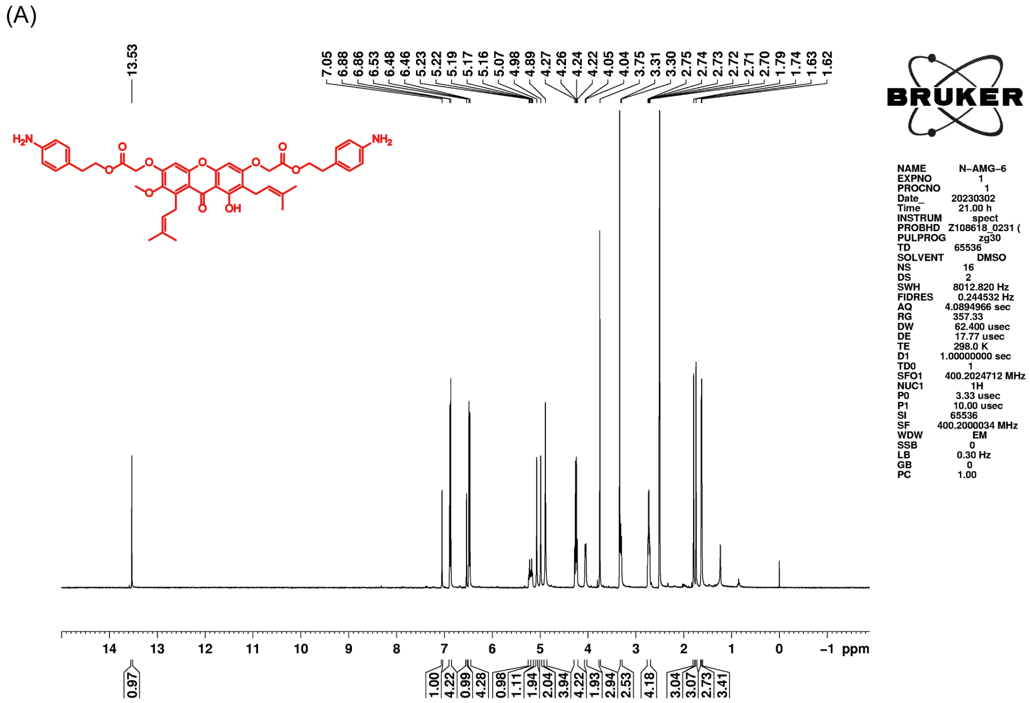

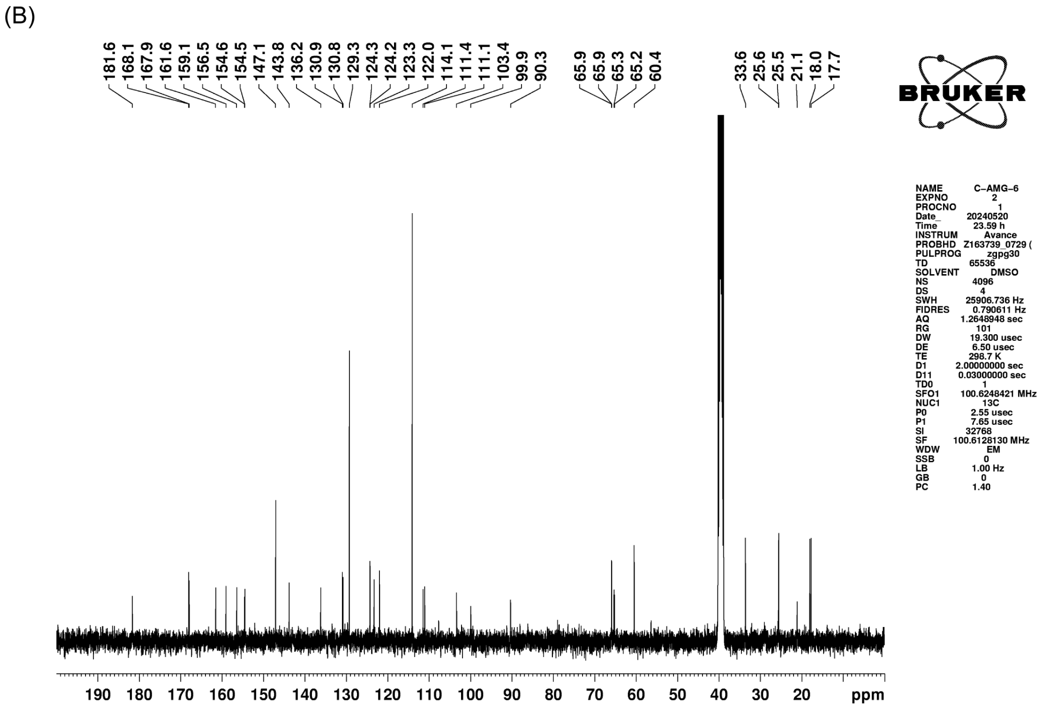

Figure S27 ^{1}H NMR (400 MHz, DMSO-*d6*) (A) and ^{13}C NMR (100 MHz, DMSO-*d6*) (B) spectrum of A30.

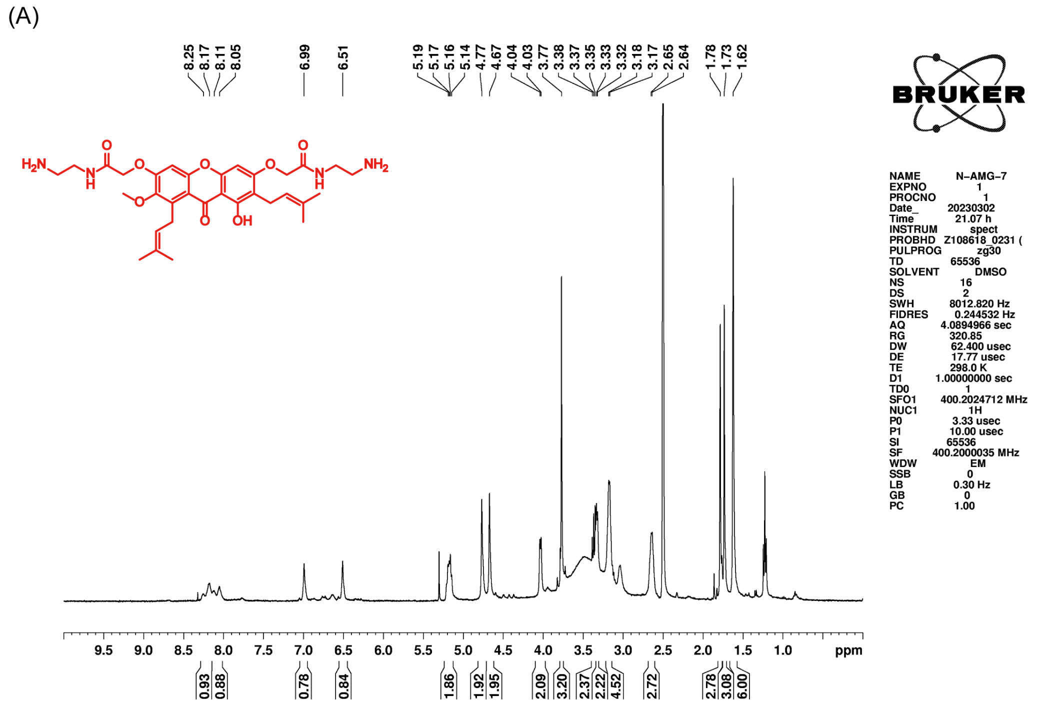

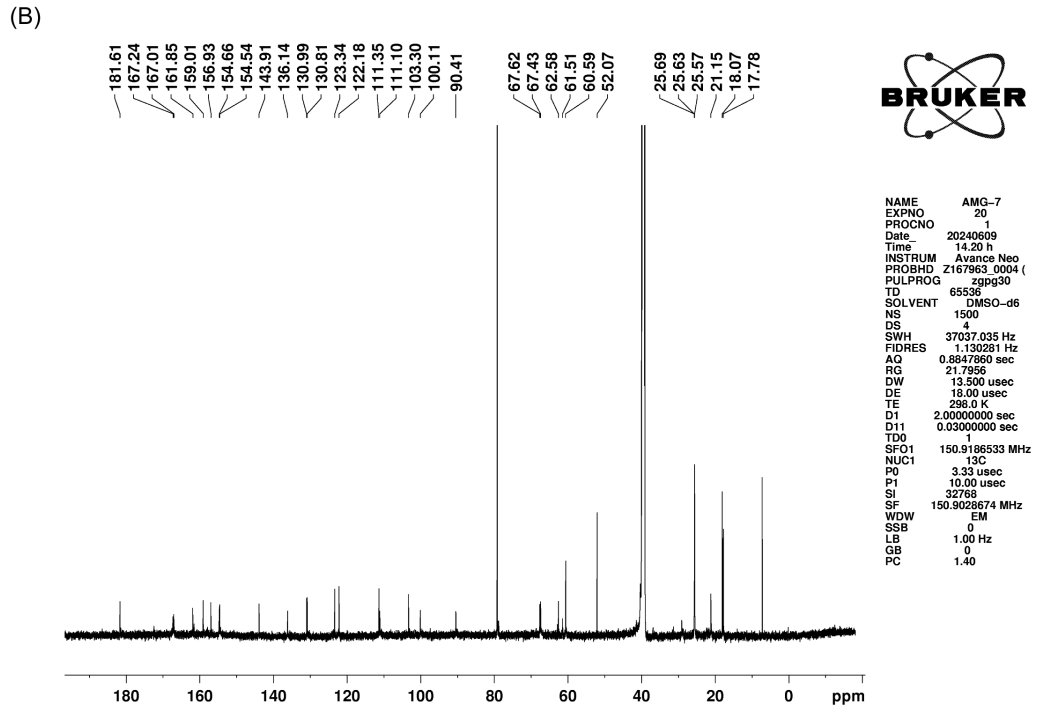

Figure S28 ^{1}H NMR (400 MHz, DMSO-*d6*) (A) and ^{13}C NMR (150 MHz, DMSO-*d6*) (B) spectrum of A32.

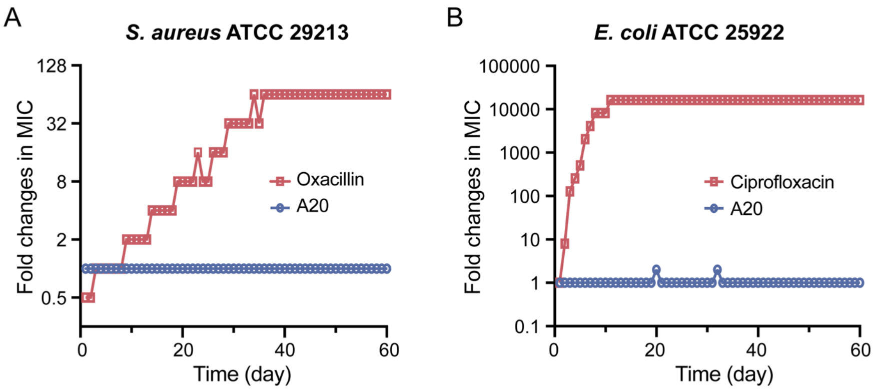

Figure S29 Resistance development of A20.

S. aureus ATCC 29213 (A) and *E. coli* ATCC 25922 (B) were passaged daily in sub-inhibitory concentrations of A20, oxacillin or ciprofloxacin.

Experiments were performed as three biologically independent experiments. Data are presented as mean ± SD (n = 3).

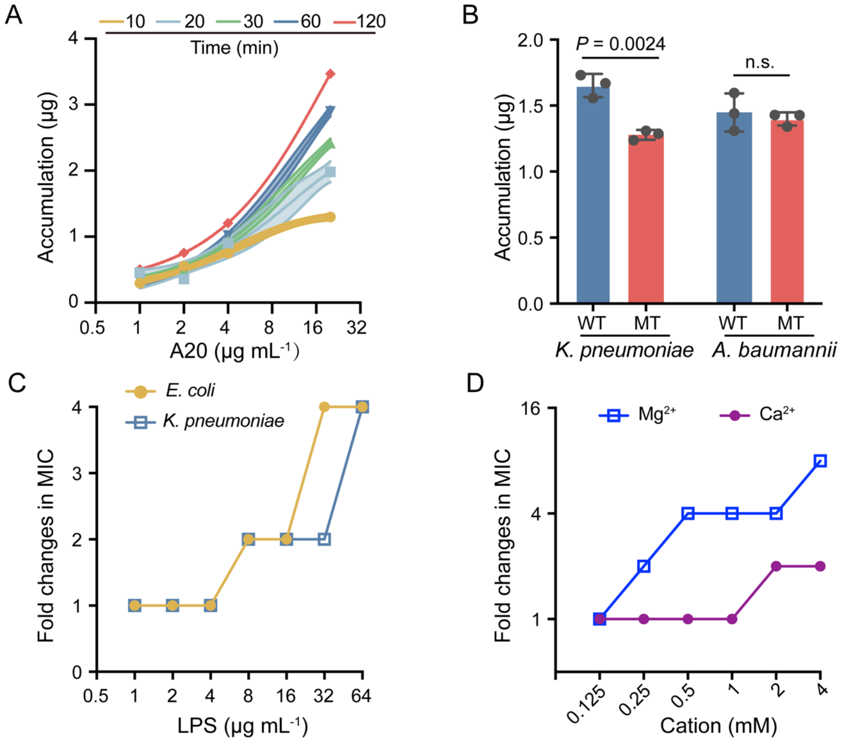

Figure S30 Self-promoted uptake pathway of A20 for transmembrane transportation.

(A) Intracellular accumulation kinetics of A20 in *E. coli*.

(B) Intracellular accumulation of A20 in the wild-type and LPS-deficient strains. *K. pneumoniae* WT, *K. pneumoniae* ATCC 43816; *K. pneumoniae* MT, *K. pneumoniae* ATCC 43816 *Δwaac*; *A. baumannii* WT, *A. baumannii* 7-2; *A. baumannii* MT, *A. baumannii* 7-2 ΔLPS.

(C) Fold changes in MICs of A20 with exogenetic LPS.

(D) Fold changes in MICs of A20 in the presence of cations.

Experiments were performed as three biologically independent experiments. Data are presented as mean ± SD (n = 3). Statistical analysis was conducted using One-way ANOVA test, and $P < 0.05$ was considered statistically significant.

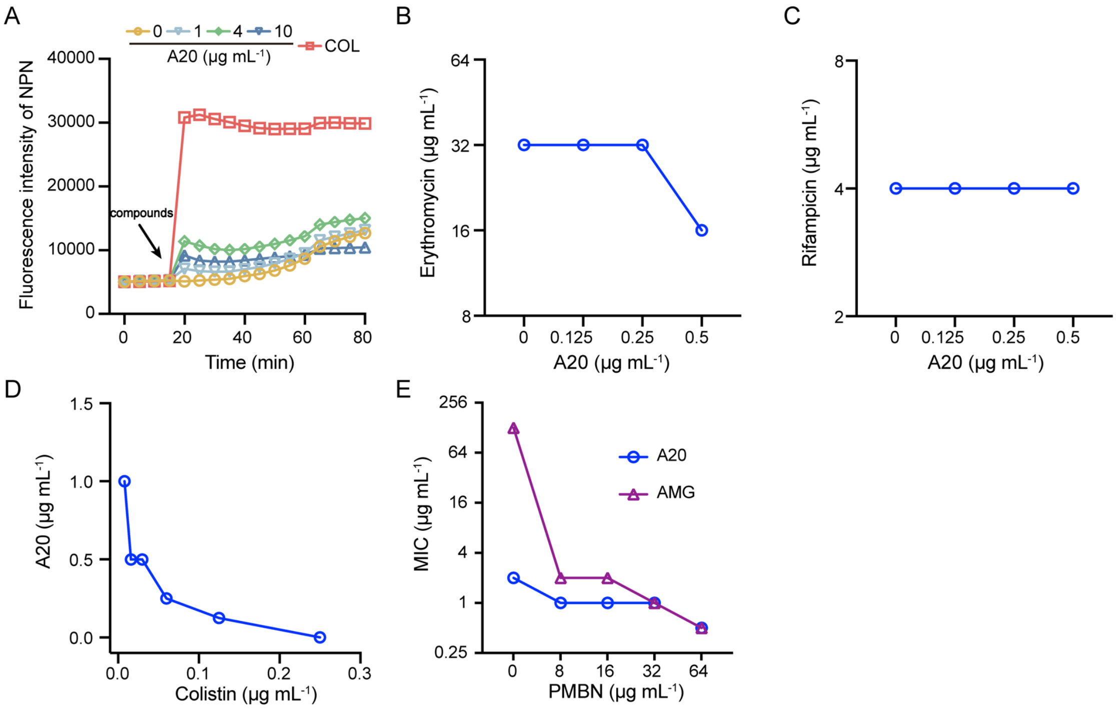

Figure S31 Bacterial OM permeability under the treatment of A20.

(A) OM permeability of *E. coli* under the treatment of A20, probed with 1-N-phenylnaphtylamine. The excitation wavelength is 350 nm and the emission wavelength is 420 nm.

(B-C) Antibacterial activity of A20 with erythromycin (B) or rifampicin (C) against *E. coli* ATCC 25922.

(D) Synergy of A20 and colistin against *E. coli* ATCC 25922.

(E) Synergy of A20 and PMBN against *E. coli* ATCC 25922.

Experiments were performed as three biologically independent experiments. Data are presented as mean ± SD (n = 3).

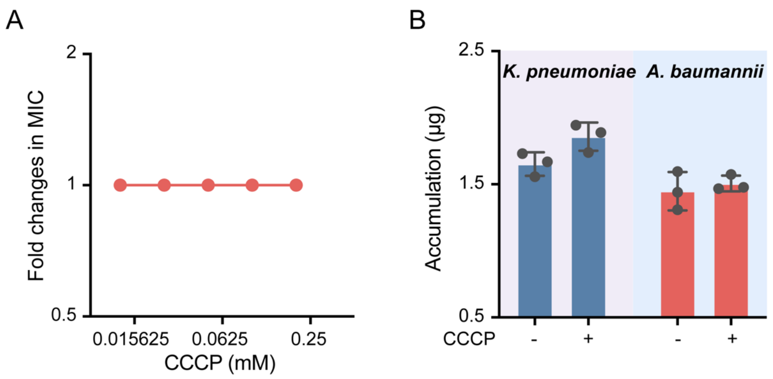

Figure S32 Antibacterial activity and intracellular accumulation of A20 under the treatment of PMF disruptor.

(A) Fold changes in MICs of A20 in the presence of CCCP against *E. coli* ATCC 25922.

(B) Intracellular accumulation of A20 under the treatment of CCCP in Gram-negative bacteria including *K. pneumoniae* ATCC 43816 and *A. baumannii* 7-2.

Experiments were performed as three biologically independent experiments. Data are presented as mean ± SD (n = 3).

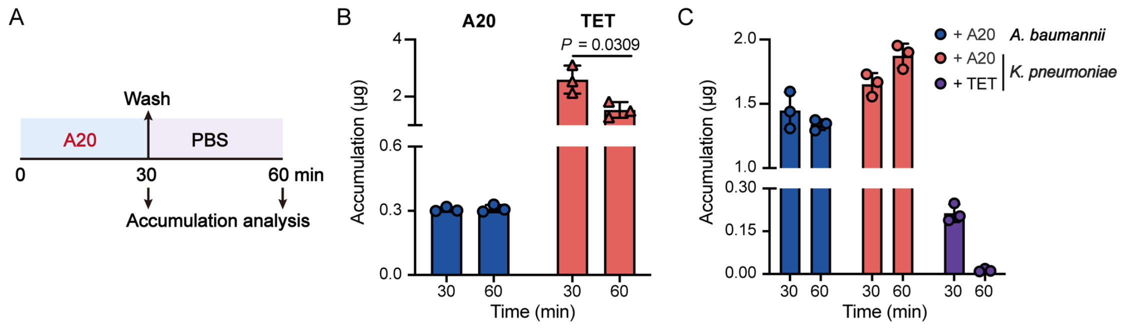

Figure S33 Efflux of A20 in Gram-negative bacteria.

(A) Scheme of efflux determination of A20.

(B) Residual A20 in *E. coli* ATCC 25922 after incubation in PBS at one hour.

(C) Residual A20 in *K. pneumoniae* ATCC 43816 and *A. baumannii* 7-2 after incubation in PBS at one hour.

Experiments were performed as three biologically independent experiments. Data are presented as mean ± SD (n = 3). Statistical analysis was conducted using the unpaired Student's t-test, and $P < 0.05$ was considered statistically significant.

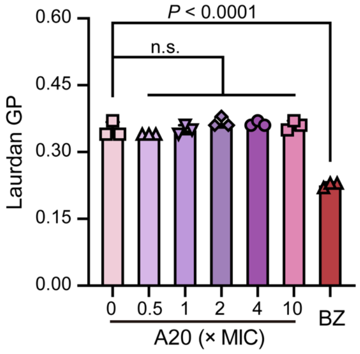

Figure S34 Membrane fluidity of *E. coli* ATCC 25922 after treatment with A20.
After treatment with A20 for one hour, the membrane fluidity of *E. coli* ATCC 25922 was probed by Laurdan. Benzyl alcohol (BZ) was used as positive control. Experiments were performed as three biologically independent experiments. Data are presented as mean ± SD (n = 3). Statistical analysis was conducted using One-way ANOVA test, and P < 0.05 was considered statistically significant.

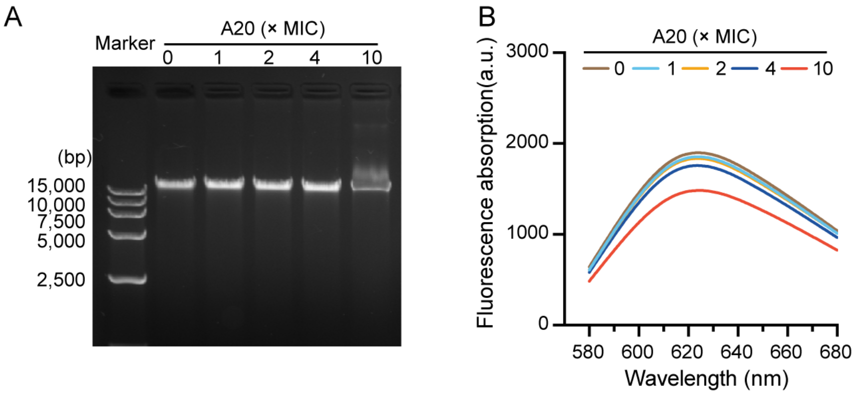

Figure S35 DNA binding evaluation of A20.

(A) Gel retardation assay of A20 and DNA.

(B) Propidium-iodide (PI) displacement assay after incubation with A20 and DNA for one hour.

Experiments were performed as three biologically independent experiments. Data are presented as mean ± SD (n = 3).

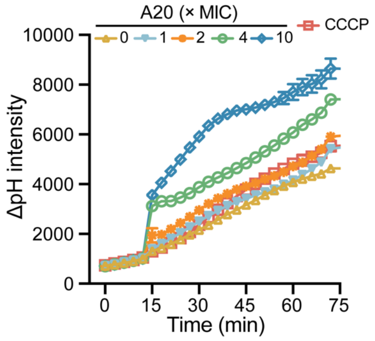

Figure S36 ΔpH kinetics in *E. coli* ATCC 25922 in the presence of A20.

The intracellular pH was determined by measuring the fluorescence intensity of BCECF with the excitation/emission wavelength at 488 nm/535 nm.

Experiments were performed as three biologically independent experiments. Data are presented as mean ± SD (n = 3).

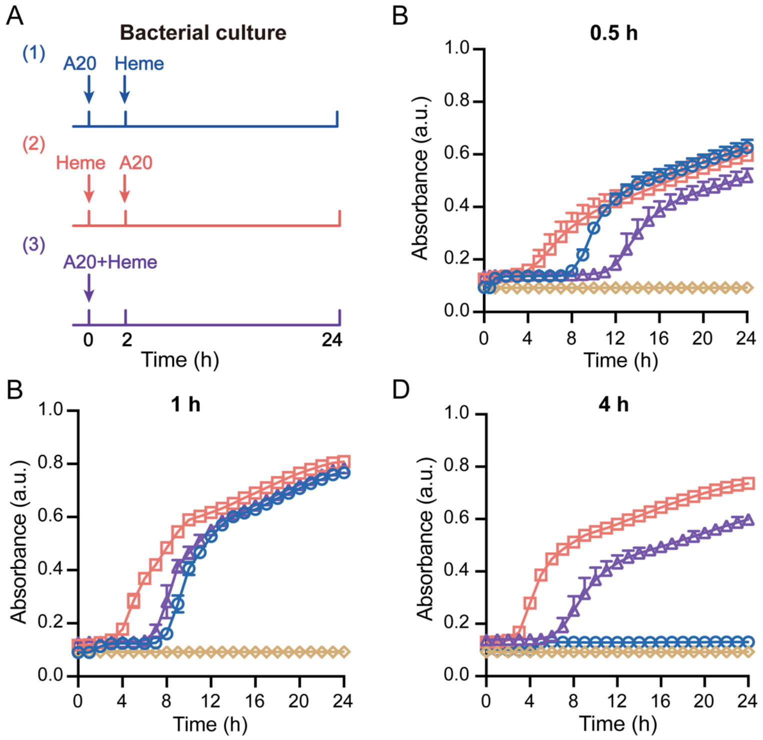

Figure S37 Effects of hemin on the antibacterial activity of A20.

(A) Scheme of experimental design.

(B-D) Growth curves of *E. coli* ATCC 25922 under the treatment of A20, hemin, and both thereof at half of an hour (B), one hour (C), and four hours (D).

Experiments were performed as three biologically independent experiments. Data are presented as mean ± SD (n = 3).

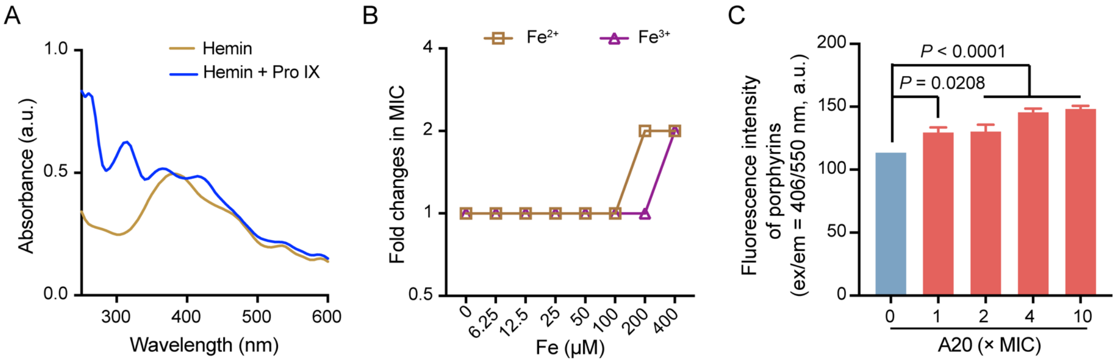

Figure S38 A20 causes the disruption of bacterial hemin.

(A) UV−vis spectra of protoporphyrin IX (20 μM) with A20 in 200 mM HEPES (pH 7.0).

(B) Fold changes in MICs of A20 in the presence of Fe^{2+}/Fe^{3+}.

(C) Intracellular porphyrins in *E. coli* after treatment with A20 for one hour. *E. coli* ATCC 25922 was used as the model strain.

Experiments were performed as three biologically independent experiments. Data are presented as mean ± SD (n = 3). Statistical analysis was conducted using One-way ANOVA test, and $P < 0.05$ was considered statistically significant.

Table S1 Strains used in this study.

Strains	Description	Re.
**Gram-positive bacteria**		
Staphylococcus aureus ATCC 29213	MSSA	
S. aureus T144	MRSA [(*mecA*, *tet* (A)]	(*1*)
Enterococcus faecium ATCC 29212		
E. faecium VRE 10	VRE	(*2*)
**Gram-negative bacteria**		
Acinetobacter baumannii 7-2	wildtype	(*3*)
A. baumannii 7-2 ΔLPS	LPS deficient; colistin resistant	(*3*)
A. baumannii 176	wildtype	(*3*)
A. baumannii 176 ΔLPS	LPS deficient; colistin resistant	(*3*)
Escherichia coli ATCC 25922		
E. coli B2	*mcr-1*, $bla_{\text{NDM-5}}$, $bla_{\text{TEM-1B}}$, $bla_{\text{OXA-10}}$, $bla_{\text{CTX-M-14}}$, *tet*(A), *aad*A, *ahp*(4), *oqxAB*, *mdfA*, *fosA3*	*(4)*
E. coli J53		(*5*)
E. coli J53 ΔOmpF	OmpF deficient	(*5*)
Klebsiella pneumoniae ATCC 43816		
K. *pneumoniae* ATCC 43816 ΔwaaC		(*6*)

ATCC, American Type Culture Collection; MRSA, methicillin-resistant *Staphylococcus aureus*; *mcr*-1, mobile colistin resistance gene; *tet*(A), tetracyclines resistant gene; $bla_{\text{NDM-5}}$, carbapenems resistant gene; $bla_{\text{TEM-1B}}$, penicillins resistant gene; $bla_{\text{OXA-10}}$, carbapenems resistant gene; $bla_{\text{CTX-M-14}}$, β-lactams especially cefotaxime resistant gene; *aadA*, aminoglycosides resistant gene; *ahp(4)*, hygromycin B resistant gene; *oqxAB*, quinolones resistant gene; *mdfA*, cationic or zwitterionic lipophilic compounds resistant gene; *fosA3*, fosfomycin resistant gene.

Table S2 Representative biochemical and chemical reagents used in this study.

Substance	CAS No.	Supplier
α-Mangostin	6147-11-1	Chengdu Biopurity Phytochemicals Co., Ltd.
Dichloromethane	75-09-2	Shanghai Titan
N, N-dimethyldoemamide	68-12-2	Shanghai Titan
Dimethyl sulfoxide	67-68-5	Shanghai Titan
Petroleum ether	8032-32-4	Shanghai Titan
Ethyl acetate	141-78-6	Shanghai Titan
Tetrahydrofuran	109-99-9	Shanghai Titan
Potassium bicarbonate	298-14-6	Shanghai Titan
1,2-dibromoethane	106-93-4	Shanghai Titan
1,3-dibromopropane	109-64-8	Shanghai Titan
1,4-dibromobutane	110-52-1	Shanghai Titan
ethylamine	75-04-7	Shanghai Titan
N-methylpropylamine	627-35-0	Shanghai Titan
Dimethylamine	124-40-3	Shanghai Titan
triethanolamine	102-71-6	Shanghai Titan
Piperidine	110-89-4	Shanghai Titan
1-(3-dimethylaminopropyl)-3-ethylcarbodiimide hydrochloride	25952-53-8	Shanghai Titan
4-dimethylaminopyridine	1122-58-3	Shanghai Titan
N-Boc-ethylenediamine hydrochloride	79513-35-2	Shanghai Titan
Sodium tert-butoxide	865-48-5	Shanghai Titan
Ethyl chloroacetate	105-39-5	Shanghai Titan
Lithium hydroxide	1310-65-2	Shanghai Titan
Ammonium chloride	12125-02-9	Shanghai Titan
2-aminoethanol	141-43-5	Shanghai Titan
Sodium sulfate	7757-82-6	Shanghai Titan
Sodium bicarbonate	144-55-8	Shanghai Titan
Copper cyanide	544-92-3	Shanghai Titan
1*H*-pyrazole-1-carboxamidine Hydrochloride	4023-02-3	Shanghai Titan
diisopropylethylamine trihydrofluoride	131600-43-6	Shanghai Titan
Diethylamine	109-89-7	Shanghai Titan

Table S3 Antibacterial activity (MIC, μg mL^{-1}) of natural products for hit screening.

No.	Com.	Gram-positive bacteria	Gram-negative bacteria			
		S. aureus ATCC 29213	*A. baumannii* 176	*A. baumannii* 176 ΔLPS	*A. baumannii* 7-2	*A. baumannii* 7-2 ΔLPS
1	α-Mangostin	1	> 128	0.125	> 128	0.125
2	Rhein	32	> 128	64	> 128	64
3	Protocatechuic acid	64	32	32	32	32
4	Genkwanin	64	16	16	16	16
5	Emodin anthrone	16	> 128	4	> 128	4
6	Isobavachalcone	4	> 128	1	> 128	1
7	Apigenin	32	> 128	16	> 128	16
8	Phloretin	128	> 128	64	> 128	64
9	Shikonin	64	> 128	16	> 128	16
10	6-Gingerol	64	> 128	128	> 128	128
11	Rubimaillin	32	> 128	32	> 128	32
12	Xanthohumol	16	> 128	1	> 128	1
13	Purpurin	16	64	16	64	16
14	2-Hydroxychavicol	128	> 128	64	> 128	64
15	Licochalcone A	4	> 128	1	> 128	1
16	6-Shogaol	32	> 128	32	> 128	32
17	Leonurine	128	> 128	128	> 128	128
18	Gramine	128	> 128	128	> 128	128
19	Syringic acid	128	> 128	128	> 128	128
20	Zingerone	128	> 128	128	> 128	128

21	Fraxetin	16	> 128	128	> 128	128
22	Danshensu	128	> 128	128	> 128	128
23	Oxysanguinarine	16	32	2	32	2
24	Sanguinarium chloride	128	> 128	128	> 128	128
25	Dihydrosanguinarine	64	> 128	64	> 128	64
26	Sanguinarine	4	16	2	16	2
27	Allocryptopine	128	> 128	128	> 128	128
28	6-Methoxyl-dihydro-chelerythrine chloride	16	32	2	32	2
29	Chelidonine	4	32	2	32	2
30	Bacopaside I	128	> 128	128	> 128	128
31	Bacoside A3	128	> 128	128	> 128	128
32	Baicalein	64	128	32	128	32
33	6-Methoxy-dihydrosanguinarine	16	16	2	16	2
34	Wogonin	64	> 128	64	> 128	64
35	Dihydrochelerythrine	32	> 128	8	> 128	8
36	Colistin	/	0.0625	64	0.0625	64
37	Kanamycin	32	1	0.25	1	0.25

Table S4 The physicochemical parameters of AMG derivatives.

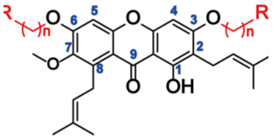

Com.	n	R	$clogD_{7.4}$	TPSA	pKa	MW
**AMG**	0	–H	3.30	96.22	6.52	410.47
**A6**	1		3.67	74.22	6.90	486.56
**A7**	2		4.73	63.63	9.55	610.88
**A8**	3	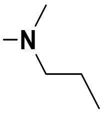	4.46	80.70	9.89	636.87
**A9**	4		4.63	80.70	10.30	664.93
**A10**	2		4.32	80.70	9.24	608.82
**A11**	3	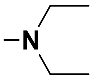	4.37	80.70	9.88	636.87
**A12**	4		4.55	80.70	10.30	664.93
**A13**	2		3.55	98.28	9.21	552.71
**A14**	3	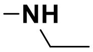	3.71	98.28	9.91	580.77
**A15**	4		3.93	98.28	10.34	608.82
**A16**	2		3.47	80.70	9.19	552.71
**A17**	3	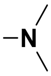	3.64	80.70	9.84	580.77
**A18**	4		3.93	80.70	10.25	608.82
**A19**	2	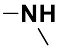	3.20	98.28	9.20	524.66
**A20**	2		2.79	126.26	9.06	496.60
**A21**	3	$-NH_2$	3.08	126.26	9.82	524.66
**A22**	4		3.44	126.26	10.27	552.71
**A23**	2	-OH	3.07	114.68	6.93	498.57
**A24**	2	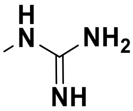	1.25	198.02	9.18	580.69

**A32**	1	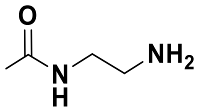	1.45	184.46	9.03	610.71

The physicochemical parameters were calculated using ADMETlab 2.0 (https://admetmesh.scbdd.com/).

Table S5 Antibacterial activity (MIC, μg mL^{-1}) and physicochemical parameters of PCA derivatives.

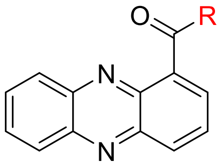

Com.	R	$clogD_{7.4}$	TPSA	pKa	MW	Gram-positive bacteria		Gram-negative bacteria
						S. aureus ATCC 29213	*E. faecalis* ATCC 29212	*E. coli* ATCC 25922
**PCA**	OH	1.47	63.08	4.80	224.22	64	128	> 128
**P1**	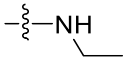	2.50	54.88	14.00	251.29	64	> 128	> 128
**P2**	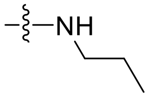	2.95	54.88	14.00	265.32	> 128	> 128	> 128
**P3**	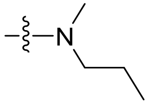	2.78	46.09	14.00	279.34	> 128	> 128	> 128

**P4**	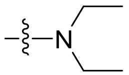	2.69	46.09	14.00	279.34	> 128	> 128	> 128
**P5**	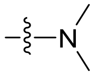	2.21	46.09	14.00	251.29	> 128	> 128	> 128
**P6**	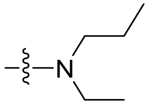	3.02	46.09	14.00	293.37	> 128	> 128	> 128
**P7**	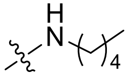	3.55	54.88	14.00	293.37	> 128	> 128	> 128
**P8**	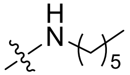	3.72	54.88	14.00	307.40	64	64	> 128
**P9**	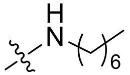	3.88	54.88	14.00	321.42	> 128	> 128	> 128
**P10**	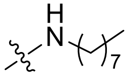	/	54.88	14.00	335.45	> 128	> 128	> 128
**P11**	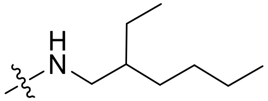	/	54.88	14.00	335.45	> 128	> 128	> 128
**P12**	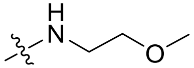	2.28	64.11	14.00	281.32	> 128	> 128	> 128
**P13**	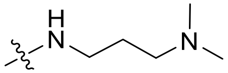	2.17	58.12	14.00	308.39	> 128	> 128	> 128
**P14**	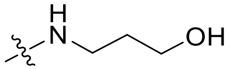	1.91	75.11	14.00	281.32	> 128	> 128	> 128

**P15**	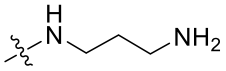	1.49	80.9	14.00	280.33	64	32	> 128
**P16**	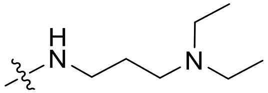	2.64	58.12	14.00	336.44	> 128	> 128	> 128
**P17**	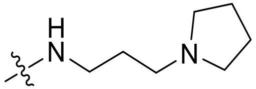	2.46	58.12	14.00	334.42	> 128	> 128	> 128
**P18**	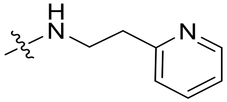	2.62	67.77	14.00	328.38	> 128	> 128	> 128
**P19**	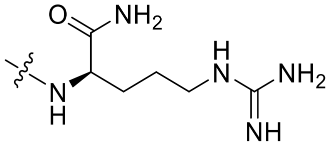	0.39	159.87	8.35	379.42	> 128	> 128	> 128
**Gentamicin**		/	/	/	/	0.5	1	1
**Doxycycline**		/	/	/	/	0.25	1	2

Table S6 Antibacterial activity (MIC, μg mL^{-1}) and physicochemical parameters of RHE derivatives.

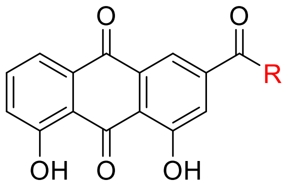

Com.	R	clogD$_{7.4}$	TPSA	pKa	MW	Gram-positive bacteria		Gram-negative bacteria
						S. aureus ATCC 29213	*E. faecalis* ATCC 29212	*E. coli* ATCC 25922
**Rhein**	OH	1.88	111.90	4.30	284.22	32	64	> 128
**R1**	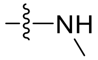	/	103.70	6.15	297.27	64	> 128	> 128
**R2**	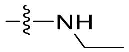	2.39	103.70	6.15	311.29	64	> 128	> 128
**R3**	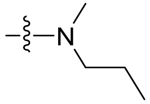	2.55	94.91	6.15	339.35	64	> 128	> 128
**R4**	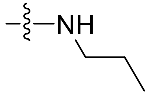	2.65	103.7	6.15	325.32	64	> 128	> 128

**R5**	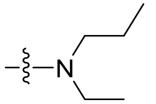	2.73	94.91	6.15	353.37	64	> 128	> 128
**R6**	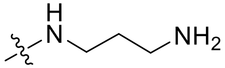	0.76	129.72	7.36	340.34	32	32	> 128
**R7**	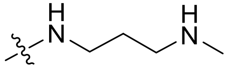	1.41	115.73	7.36	354.36	**16**	**16**	**> 128**
**R8**	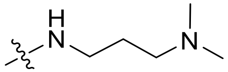	2.02	106.94	7.36	368.39	32	16	> 128
**R9**	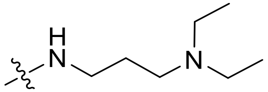	2.39	106.94	7.36	396.44	32	64	> 128
**R10**	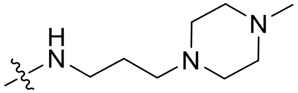	2.73	110.18	7.36	423.47	32	128	> 128
**R11**	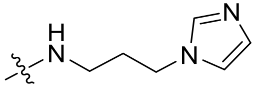	1.91	121.52	6.80	391.38	64	128	> 128
**R12**	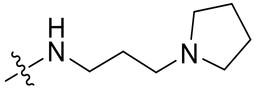	2.32	106.94	7.36	394.42	**16**	**32**	**> 128**
**R13**	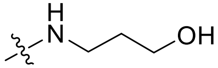	1.91	123.93	6.14	341.32	64	128	> 128
**R14**	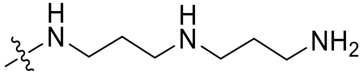	0.09	141.75	8.80	397.43	128	> 128	> 128

**R15**	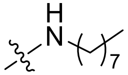	3.49	103.70	6.15	395.46	64	> 128	> 128
**R16**	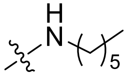	3.08	103.70	6.15	367.40	> 128	> 128	> 128
**R17**	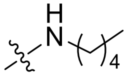	2.85	103.70	6.15	353.37	32	> 128	> 128
**Gentamicin**		/	/	/	/	0.5	1	1
**Doxycycline**		/	/	/	/	0.25	1	2

Table S7 MIC (μg mL^{-1}) of six AMG analogs with potent broad-spectrum activity.

Strains	Types	**Compounds** (μg mL^{-1})					
		**A16**	**A17**	**A19**	**A20**	**A24**	**A32**
**Gram-positive bacteria**							
Staphylococcus aureus ATCC 29213		1	0.5	0.5	0.5	0.5	2
S. aureus 231		1	1	1	1	1	2
S. aureus 232		1	1	2	2	1	2
S. aureus 224		2	2	1	2	1	2
S. aureus 233-2		2	1	2	2	2	2
S. aureus 223-1		2	2	1	1	2	4
MRSA DL44	MRSA	2	2	1	1	2	4
MRSA DY82	MRSA	4	1	0.5	0.5	2	2
MRSA SF30	MRSA	2	0.5	0.5	0.5	1	2
MRSA 74	MRSA	4	1	1	1	1	4
MRSA 155	MRSA	4	1	1	1	2	2

Enterococcus faecium 20HB9RX11	2	2	2	2	1	2
E. faecium 20HB9RX12	2	2	2	2	1	2
E. faecium 20HB9RX15	2	2	1	1	2	2
E. faecium 20HB9RX27	2	2	1	1	2	2
E. faecium 20HB9RX36	2	4	1	1	1	4
E. faecalis 20HB9RX13	2	4	1	1	1	4
E. faecalis 20HB9RX19	4	2	1	1	1	2
E. faecalis 20HB9RX24	2	2	1	1	2	2
E. faecalis 20HB9RX2	2	4	2	2	4	2
E. faecalis 20HB9RX2	2	4	2	2	4	4
Streptococcus suis ATCC 43765	2	1	1	1	1	4
S. suis T73	1	1	1	1	2	2
S. suis B73-1	1	1	1	1	1	2
S. suis B40	1	2	1	1	2	2

S. suis B41	1	1	1	1	1	2
S. suis B58	2	1	1	1	1	2
S. suis B57	1	2	2	2	2	4
S. suis B56	1	1	2	2	1	4
S. suis B43	1	1	1	0.5	1	2
Clostridium perfringens 2020sj5rx165	2	2	2	2	2	4
C. perfringens 21sx4pky10	4	4	2	2	2	4
C. perfringens 2020sj5rx13	2	4	2	2	1	4
C. perfringens 20HB8PK32	4	4	2	2	2	4
C. perfringens 20HB9RX14	2	2	2	2	2	4
C. perfringens 19SX3RX70	4	4	2	2	2	4
C. perfringens 19NM2CM20	2	2	2	2	2	4
C. perfringens 19NM1CM25	2	4	1	1	1	4
C. perfringens 19SX3FX100	2	2	2	2	2	4
C. perfringens 19NM1CM9	2	2	2	2	2	4

**MIC$_{50}$**	**2**	**2**	**1**	**1**	**2**	**2**
**Gram-negative bacteria**						
Acinetobacter baumannii 119	8	16	16	2	1	4
A. baumannii 108	8	16	16	2	2	4
A. baumannii 176	8	16	16	1	2	4
A. baumannii 34	8	16	8	1	1	2
A. baumannii 12-1	4	8	8	1	1	2
A. baumannii ATCC 17978	8	16	8	1	1	4
A. baumannii ATCC 19606	4	8	8	1	1	4
Aeromonas species 17QDFSK8BW	8	8	4	4	8	8
Aeromonas species 17QDFSK7BG	4	8	4	2	4	4
Aeromonas species 17QDFSK3BG	8	8	4	4	8	4

Aeromonas species 17QDFSK2BG	4	8	4	4	4	8
Aeromonas species 17QDFSK5BW	4	8	4	4	4	4
Aeromonas species 17QDFSK5BG	8	8	4	4	8	8
Aeromonas species 17QDFSK1BW	8	8	4	4	8	8
Aeromonas species 17QDFSK5BB	4	8	4	4	4	8
Escherichia coli ATCC 25922	2	4	1	1	2	2
E. coli 1DM25	4	4	4	4	4	4
E. coli 1DM27	4	8	4	4	4	4
E. coli 1DM31	4	8	2	2	2	2
E. coli 1DM33	4	8	4	4	2	4

E. coli 1DM30		4	8	4	4	4	4
E. coli 1DM4		4	8	4	4	2	4
E. coli 1DM47		4	4	4	4	4	4
E. coli 1DM50		2	4	4	4	2	4
E. coli 1DM44		4	4	4	4	4	4
E. coli B2	bla_{NDM5} +*mcr-1*	4	4	2	1	4	2
E. coli 16DQZXRF1SBC	*mcr*	4	4	4	2	4	4
E. coli 16QD8DZ68BC	*mcr*	4	8	4	4	4	4
E. coli 16QD1AE8RC	*mcr*	4	4	4	4	4	4
E. coli 16QD1AZ6RC	*mcr*	4	8	4	2	4	4
E. coli 17QD3AZ38RC	*mcr*	4	8	4	2	4	4
E. coli 16QDZAE6RC	*mcr*	4	8	4	4	4	4
E. coli 16QD21SDZ87BC	*mcr*	4	8	4	2	4	4
E. coli 16QD1AZ1RC	*mcr*	4	4	4	2	4	4
E. coli 15QDHSDZ80BC	*mcr*	2	4	2	2	2	2
E. coli 16QD2AZ1RC	*mcr*	2	4	2	2	4	2

E. coli SH130	*mcr*	2	4	2	2	2	4
E. coli 32-2	*tet*(X4)	4	4	2	2	8	4
E. coli 10R1-1	*tet*(X4)	2	4	2	2	2	4
E. coli 19QD1DZ21R	*tet*(X4)	4	8	4	2	4	4
E. coli 30-1R	*tet*(X4)	4	8	4	2	8	4
E. coli 14-3R	*tet*(X4)	4	8	4	2	4	4
E. coli 26-1	*tet*(X4)	4	8	4	2	4	4
E. coli 6-1	*tet*(X4)	2	2	2	2	2	2
E. coli 18QD2DZ22WR	*tet*(X4)	2	4	2	1	4	2
E. coli 18QD11MM2-1R	*tet*(X4)	2	4	2	2	2	4
E. coli 18QD2NN7WR	*tet*(X4)	2	4	2	2	4	2
E. coli 18QD2DZ56-3-5R	*tet*(X4)	2	4	2	1	2	2
E. coli 18QD3DZ3RR	*tet*(X4)	2	4	1	1	2	2
E. coli 50-2R	*tet*(X4)	2	4	1	1	2	2
E. coli 45-1R	*tet*(X4)	2	4	2	2	2	4
E. coli 18QD11MM2-35R	*tet*(X4)	2	4	2	1	2	4

E. coli 18QD4NM2RR	*tet*(X4)	4	4	2	2	4	2
E. coli 17QD5RH11RK	*bla*$_{NDM}$	4	8	4	4	8	4
E. coli 17QD5RZ8RK	*bla*$_{NDM}$	4	4	2	2	4	4
E. coli 17QD5KZ18RK	*bla*$_{NDM}$	4	4	2	2	4	4
E. coli 17QD3RP3RK	*bla*$_{NDM}$	2	4	2	2	2	2
E. coli 17QD3RP2RK	*bla*$_{NDM}$	2	4	2	2	2	2
E. coli 17QD3RP15RK	*bla*$_{NDM}$	2	4	2	2	4	4
E. coli 17QD5RH5RK	*bla*$_{NDM}$	4	8	2	2	4	4
E. coli 17QD3RH5RK	*bla*$_{NDM}$	4	8	2	2	2	2
E. coli 17QD5RZ22RK	*bla*$_{NDM}$	2	4	2	2	4	4
E. coli 17QD5RZ17RK	*bla*$_{NDM}$	4	8	2	2	2	2
E. coli 18QD2RY1RK	*bla*$_{NDM}$	4	4	4	4	4	4
E. coli 17QD5RZ35RK	*bla*$_{NDM}$	2	4	2	2	2	2
E. coli 17QD5RZ28RK	*bla*$_{NDM}$	4	4	2	2	2	4
E. coli 17QD5RZ25RK	*bla*$_{NDM}$	2	4	2	2	4	2
E. coli 17QD5RZ23RK	*bla*$_{NDM}$	2	4	2	2	2	4

E. coli 17QD3RF20RK	*bla*$_{NDM}$	2	4	2	2	2	2
E. coli 17QD3RF14RK	*bla*$_{NDM}$	4	4	4	4	4	4
E. coli 17QD5RH4RK	*bla*$_{NDM}$	2	4	2	1	2	2
E. coli 17QD5RZ2RK	*bla*$_{NDM}$	2	4	2	2	2	2
Salmonella species 90		2	4	4	4	4	4
Salmonella species 89		2	4	4	4	4	4
Salmonella species 181		4	4	4	4	4	4
Salmonella species 236		4	8	8	4	4	4
Salmonella species 206		4	4	4	4	4	4
Salmonella species 92		4	4	4	4	8	4
Salmonella species 61		4	4	4	4	4	4
Salmonella species 190		4	4	4	4	4	8
Salmonella species 88		2	4	4	4	4	4
Haemophilus parasuis 2BY2		2	4	4	4	4	4
H. parasuis BY20-2		4	8	4	4	8	4

H. parasuis BY27	4	16	8	8	16	4
H. parasuis BY59-1	4	16	8	8	8	8
H. parasuis TZ73-2	2	8	4	4	8	4
H. parasuis CY12	4	16	8	8	8	8
H. parasuis SD101-1	4	16	8	8	8	8
H. parasuis BY29-2	8	8	8	8	8	8
H. parasuis BY10-2	8	8	8	4	8	4
H. parasuis BY13	8	8	8	8	8	8
Pasteurella multocida PMSH-2	2	4	2	2	4	2
P. multocida PMSX-20	2	4	2	2	4	2
P. multocida PMSX-4	1	2	1	1	2	1
P. multocida PMSC-5	1	2	1	1	2	2
P. multocida PMSC-3	1	2	1	1	2	1
P. multocida PMSH-1	2	4	2	2	4	2
P. multocida PMSX-24	1	2	1	2	2	1

P. multocida PMSC-1	1	2	1	1	2	2
P. multocida PMSC-2	1	2	1	1	2	2
Pseudomonas aeruginosa PAO1	16	> 32	> 32	4	16	8
P. aeruginosa N18-1	16	> 32	> 32	32	32	16
P. aeruginosa 1015	8	> 32	> 32	8	16	8
P. aeruginosa N16-3	16	> 32	> 32	8	32	16
P. aeruginosa N16-2	16	> 32	> 32	8	8	8
P. aeruginosa G414-1	16	> 32	> 32	4	16	8
P. aeruginosa ZE-6	32	> 32	> 32	8	16	16
P. aeruginosa M6-4	8	> 32	> 32	8	16	8
P. aeruginosa 1204	16	> 32	> 32	8	16	8
P. aeruginosa 1802	16	> 32	> 32	4	16	8
Klebseilla pneumoniae ATCC 43816	4	4	4	2	4	2
K. pneumoniae JQ2707	8	8	4	4	4	4

K. pneumoniae JC4209	8	8	4	2	8	4
K. pneumoniae JC4279	8	8	4	4	4	4
K. pneumoniae JC4057	4	8	4	4	8	4
K. pneumoniae JC4152	2	4	2	2	4	2
K. pneumoniae JC3619	4	8	2	2	4	2
K. pneumoniae JC3853	4	8	4	4	4	4
K. pneumoniae JC3493	4	4	4	2	8	4
K. pneumoniae JQ2989	4	8	4	4	4	4
**MIC_{50}**	**4**	**4**	**4**	**2**	**4**	**4**

Table S8 Antibacterial activity of AMG analogs and commercial antibiotics.

**Strains**	**Description**	**Compounds** (μg mL^{-1})									
		**A16**	**A17**	**A19**	**A20**	**A24**	**A32**	**FFA**	**GEN**	**COL**	**DOX**
**Gram-positive pathogens**											
S. aureus ATCC 29213		1	0.5	0.5	0.5	0.5	2	4	0.5	> 128	0.28
S. aureus T144	MRSA	2	1	1	1	1	2	32	128	64	8
S. aureus DL44	MRSA	2	2	1	1	2	4	> 128	> 128	128	8
S. aureus DY82	MRSA	4	1	0.5	0.5	2	2	32	128	128	8
S. aureus SF30	MRSA	2	0.5	0.5	0.5	1	2	32	128	128	4
S. suis ATCC43765		2	1	1	1	1	4	2	8	> 128	0.25
S. suis B66		2	1	1	1	1	4	2	4	> 128	4
S. suis B6		2	2	1	2	2	4	2	8	> 128	8
S. suis Y26		1	2	1	1	1	2	16	1	> 128	8
S. suis B16		2	2	2	2	2	2	2	8	> 128	8
S. suis Y66		2	4	2	2	2	4	> 128	1	> 128	8
**Gram-negative pathogens**											
E. coli ATCC 25922		2	4	1	1	2	2	16	1	0.25	32

E. coli B2	*mcr-1*+$bla_{\text{NDM-5}}$	4	4	2	1	4	2	> 128	> 128	4	32
*E. coli*16QD1AZ6RC	*mcr*	4	8	4	2	4	4	> 128	128	4	> 128
E. coli 33W1-1	*tet*(X4)	4	8	4	4	4	4	> 128	2	0.25	16
E. coli 45-1R	*tet*(X4)	2	4	2	2	2	4	> 128	2	0.25	16
E. coli 50-2R	*tet*(X4)	2	4	1	1	2	2	> 128	2	0.25	16
E. coli 18QD2NN7WR	*tet*(X4)	2	4	2	2	4	2	> 128	2	0.5	> 128
E. coli 17QD3RP3RK	bla_{NDM}	2	4	2	2	2	2	> 128	2	1	> 128
E. coli 17QD5RH5RK	bla_{NDM}	4	8	2	2	4	4	8	4	0.25	32
E. coli 17QD5RZ8RK	bla_{NDM}	4	4	2	2	4	4	> 128	64	1	> 128
K. pneumoniae BL14	*tmexCD1-toprJ1*	4	4	2	2	4	4	16	> 128	2	> 128
K. pneumoniae BL20	*tmexCD1-toprJ1*	4	8	4	4	8	8	> 128	> 128	2	> 128
K. pneumoniae BL24	*tmexCD1-toprJ1*	4	8	4	1	8	8	> 128	> 128	1	> 128
Salmonella Braenderup DC-3-2JC		4	4	2	2	4	4	4	2	0.25	32
Salmonella Thompson SJS-3-4JC		8	4	4	2	4	8	4	2	1	32

Salmonella Kentuck CY-5-JC(ESBL)	4	8	2	2	4	4	> 128	> 128	0.25	> 128
Salmonella Schwarzengrund SJS-5-JT (ESBL)	4	8	4	4	4	8	> 128	> 128	0.5	> 128

FFA, florfenicol; GEN, gentamicin; COL, colistin; DOX, doxycycline.

Table S9 Half-maximal inhibitory concentrations of six AMG analogs in five non-tumor cell lines.

**Compounds**	**IC_{50}** ($\mu g\ mL^{-1}$)				
	**Vero**	**HaCaT**	**RAW264.7**	**IEC6**	**HepG2**
A16	7.82 ± 1.23	5.01 ± 0.35	8.92 ± 0.83	3.89 ± 1.25	4.67 ± 3.26
A17	6.57 ± 2.15	8.34 ± 1.34	7.58 ± 1.43	4.73 ± 3.24	6.35 ± 2.34
A19	6.98 ± 0.95	3.98 ± 0.92	5.26 ± 1.15	4.28 ± 0.86	6.43 ± 1.05
A20	9.51 ± 1.12	7.90 ± 0.54	13.01 ± 1.86	4.10 ± 1.24	7.20 ± 0.46
A21	7.25 ± 0.86	4.48 ± 1.25	8.13 ± 1.56	2.38 ± 0.45	4.31 ± 1.13
A24	8.75 ± 1.27	5.29 ± 0.74	6.49 ± 0.45	3.95 ± 0.85	5.49 ± 1.88

Table S10 Therapeutic indexes of six AMG analogs in five non-tumor cell lines.

Compounds	Therapeutic indexes				
	**Vero**	**HaCaT**	**RAW264.7**	**IEC6**	**HepG2**
A16	1.95	1.25	2.23	0.97	1.17
A17	1.64	2.08	1.90	1.18	1.59
A19	1.74	1.00	1.32	1.07	1.61
A20	4.75	3.95	6.51	2.05	3.60
A21	1.8	1.12	2.03	0.60	1.08
A24	1.95	1.25	2.23	0.97	1.17

TI, IC_{50} /MIC_{50}. The MIC_{50} of A20 refers to the concentration that inhibits the growth of 50% bacteria including 40 Gram-positive and 120 Gram-negative bacterial isolates.

Table S11 Antibacterial activity of substances against the wild-type and OmpF mutant.

**Substances**	**Channels**	**MIC** (μg mL^{-1})		**Fold Increase**
		J53	J53-ΔOmpF	
AMG	/	> 32	> 32	/
A20	/	4	4	/
colistin	LPS	0.125	0.125	/
ceftiofur	OmpF、OmpC	0.125	0.5	4
ciprofloxacin	OmpF、OmpC	0.0078	0.03125	4

References

1. Y. Liu, S. Ding, R. Dietrich, E. Martlbauer, K. Zhu, A biosurfactant-inspired heptapeptide with improved specificity to kill MRSA. *Angew Chem. Int. Ed.* **56**, 1486-1490 (2017).
2. Q. Li *et al.*, Collateral sensitivity to pleuromutilins in vancomycin-resistant *Enterococcus faecium*. *Nat. Commun.* **13**, 1888 (2022).
3. M. Song *et al.*, Plant natural flavonoids against multidrug resistant pathogens. *Adv. Sci.* **8**, e2100749 (2021).
4. M. R. Song *et al.*, A broad-spectrum antibiotic adjuvant reverses multidrug-resistant Gram-negative pathogens. *Nat. Microbiol.***5**, 1040-1050 (2020).
5. T. Xu, D. Fang, F. Li, Z. Wang, Y. Liu, A dietary source of high level of fluoroquinolone tolerance in *mcr*-carrying Gram-negative bacteria. *Research. (Wash D C)* **6**, 0245 (2023).
6. Z. Yang *et al.*, Synergy of outer membrane disruptor SLAP-S25 with hydrophobic antibiotics against Gram-negative pathogens. *J. Antimicrob. Chemother.* **78**, 263-271 (2022).
